# Bivalent Histone Modifications Orchestrate Temporal Regulation of Glucosinolate Biosynthesis During Wound‐Induced Stress Responses in Arabidopsis

**DOI:** 10.1111/pce.70232

**Published:** 2025-10-08

**Authors:** Dasom Choi, Sang Woo Lee, Dong‐Hwan Kim

**Affiliations:** ^1^ Department of Plant Science and Technology Chung‐Ang University Anseong Republic of Korea

**Keywords:** glucosinolates, H3K27me3, histone modifications, metabolites, transcriptome

## Abstract

Glucosinolates (GSLs) are secondary metabolites central to plant defence in the Brassicaceae family. While the role of histone modifications in developmental gene regulation is well studied, their function in stress‐induced secondary metabolism remains unclear. Here, we show that GSL biosynthetic genes in *Arabidopsis thaliana* are regulated by bivalent chromatin bearing both active (histone acetylation) and repressive (H3K27me3) histone marks. Components of the Polycomb Repressive Complex 2 (PRC2), including CLF, SWN and LHP1, suppress GSL gene expression, and their loss enhances GSL accumulation. Genome‐wide analyses revealed that indolic and aliphatic GSL genes are enriched with H3K27me3, with indolic genes also marked by active histone acetylation. Time‐course transcriptome and metabolite analyses using HPLC following wounding revealed distinct temporal activation patterns, with indolic GSL genes induced during the early phases and aliphatic GSL genes activated at later stages. These findings suggest that bivalent histone modifications orchestrate temporal gene expression of GSL pathways under stress, revealing a previously unrecognised epigenetic mechanism underlying plant metabolic responses to environmental stimuli.

## Summary statement

The distinct histone mark profiles between aliphatic and indolic GSL pathway genes contribute to the different transcriptional response modes of GSL pathway genes, which results in the two distinct peaks observed upon wounding stress in Arabidopsis plant.

## 1 Introduction

As sessile organisms, plants are continuously exposed to various stresses in dynamically changing environments. In addition to abiotic stresses such as heat, drought, and wounding, there is a broad array of biotic stresses including attacks by insects and pathogens. Immobilised plants must overcome these challenges in more restrictive ways, unlike animals that can move away from unfavourable environmental conditions. One of the major plant defence strategies is to produce and harness secondary metabolites which could improve plant survival, adaptation, and resilience against these stresses. For example, the *Brassicaceae* family to which the multicellular model system *Arabidopsis thaliana* (*A. thaliana*) (hereafter Arabidopsis) produces a characteristic secondary metabolite called glucosinolates (GSLs), sulphur‐and nitrogen‐containing secondary compounds.

**Figure 1:**
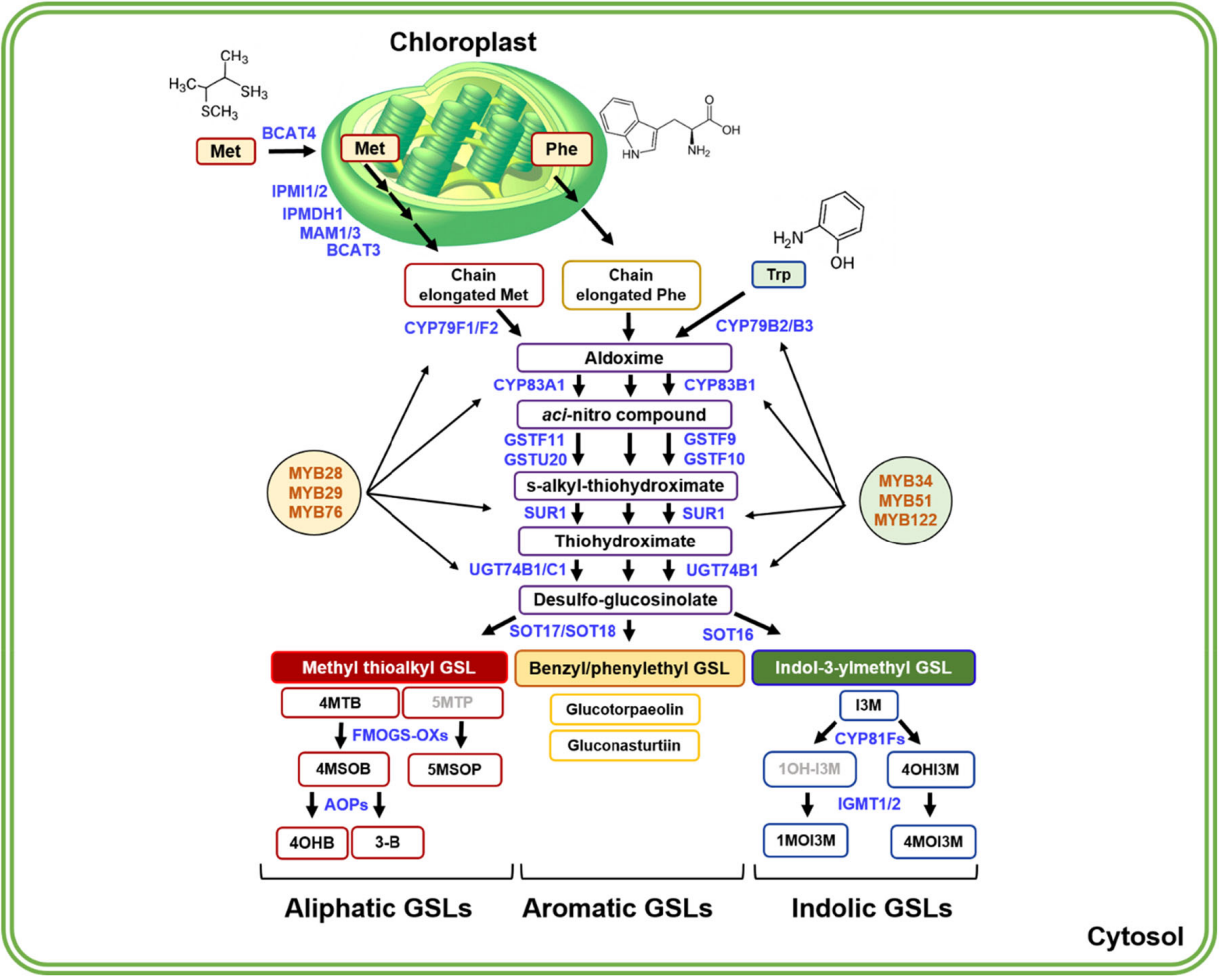
The schematic diagram showing aliphatic, aromatic, and indolic GSLs biosynthetic pathways in Arabidopsis plant cell. The biosynthesis of aliphatic, aromatic, and indolic GSL compounds generally requires three and two biosynthetic phases, respectively. The first phase ‘side‐chain elongation’ for the aliphatic GSL biosynthetic pathway takes place in the chloroplast where BCAT3, BCAT4, MAM12/3, IPMI1/2 and IMD1 play a role in the side‐chain elongation to the precursor amino acid, methionine (Met) to synthesise Aldoxime. The synthesised aldoxime from ‘side‐chain elongation’ then undergoes the second phase ‘core structure formation’ to generate desulfo‐GSL. In case of aromatic GSL biosynthesis, phenylalanine (Phe) in the chloroplast are processed to chain‐elongated Phe, and then further processed by ‘core structure formation’ which is shared with aliphatic GSLs. Then, desulfo‐GSLs enters the third phase ‘secondary modification’ phase for the production of a diversity of aliphatic and aromatic GSL compounds. In the case of indolic GSL biosynthesis, tryptophan (Trp) directly enters to the ‘core structure formation’ phase. Next, desulfo‐GSL undergoes the ‘secondary modification’ phase to generate diverse indolic GSL compounds like I3M, 4OHI3M, 1MOI3M and 4MOI3M. Major MYB TFs controlling each aliphatic and indolic GSL biosynthesis were indicated with red letters in the circle. Each catalytic step is conducted by blue‐coloured enzymes. Outest box with green two lines represents plant cell membrane and wall. Abbreviated GSLs: 4MTB, 4‐methylthiobutyl; 4MSOB, 4‐methylsulfinylbutyl; 4OHB, 4‐hydroxybutyl; 3‐B, 3‐butenyl; 5MTP, 5‐methylthiopentyl; 5MSOP, 5‐methylsulfinylpentyl; 2PE, 2‐phenylethyl; I3M, indol‐3‐ylmethyl; 1OHI3M, 1‐hydroxyindol‐3‐ylmethyl; 1MOI3M, 1‐methoxyindol‐3‐ylmethyl; 4OHI3M, 4‐hydroxyindol‐3‐ylmethyl; 4MOI3M, 4‐methoxyindol‐3‐ylmethyl.

While GSLs are synthesised at a basal level under physiological conditions, their production exponentially increases upon exposure to insect attack and wounding (Mewis et al. 2006). Over 130 GSLs reported in *Brassicaceae* family plants so far (Blažević et al. 2020), are synthesised from a handful of precursor amino acids. According to the precursor amino acids, GSLs are divided into aliphatic, indolic, and aromatic groups (Figure 1). For instance, aliphatic GSLs are derived from methionine, alanine, leucine, isoleucine, valine, or glutamate. Indolic GSLs are derived from tryptophan, while aromatic GSLs are from phenylalanine and tyrosine (Augustine and Bisht 2017). In general, the biosynthetic process of GSLs is made up of three phases: (i) side‐chain elongation of the precursor amino acids, (ii) core structure formation, and (iii) secondary modification like hydroxylation, methoxylation, oxidation, desaturation, or benzoylation at the side chain (Mitreiter and Gigolashvili 2021).

The ‘side‐chain elongation’ phase, unique to aliphatic GSLs, extends precursor amino acids through the activity of enzymes such as methylthioalkyl malate synthases (MAMs), branched‐chain aminotransferases (BCATs), and related dehydrogenases and isomerases, generating intermediates with variable chain lengths (Kroymann et al. 2001; Textor et al. 2004). In the ‘core structure formation’ phase, elongated or precursor amino acids are first converted into aldoximes by cytochrome P450s, including CYP79F1/F2 in the aliphatic pathway and CYP79B2/B3 in the indolic pathway. Subsequent reactions involving CYP83s, SUR1, UGT74 family members, and sulfotransferases lead to the production of desulfo‐glucosinolates (Mikkelsen et al. 2000; Douglas Grubb et al. 2004). Finally, during ‘secondary modification’ phase, specific enzymes diversify the core GSL structures. For example, FMO GS‐OXs, AOPs and GSL‐OH modify aliphatic GSLs, while CYP81F subfamily members and IGMTs contribute to indolic GSL diversification (Hansen et al. 2008; Li et al. 2008; Zhang et al. 2015). These sequential biosynthetic steps generate the structural diversity of GSLs observed across developmental stages and environmental conditions (Kliebenstein et al. 2006; Bednarek et al. 2011; Kissen et al. 2016; Bell 2019). Such diversity underlies the ecological functions of GSLs and makes them a focal point for studies on plant defence, evolution, and crop improvement.

Histones undergo various post‐translational modifications, including methylation, acetylation, phosphorylation and ubiquitination, which influence gene expression by altering chromatin structure or recruiting regulatory proteins (Allis and Jenuwein 2016). These modifications are mediated by three main groups of proteins: *writers* (enzymes that add marks), *erasers* (enzymes that remove marks) and *readers* (proteins that recognise and interpret the marks) (Ueda and Seki 2020). Histone writers and erasers are responsible for dynamically depositing or removing these epigenetic marks on histone tails or core domains. Among the major histone writers, Polycomb group (PcG) proteins are highly conserved across eukaryotes. They function through two key complexes: POLYCOMB REPRESSIVE COMPLEX 2 (PRC2), which catalysers H3K27me3, and PRC1, which mediates H2A ubiquitination (Schuettengruber et al. 2007; Kim and Sung 2014). In *Arabidopsis thaliana*, PRC2 components such as CURLY LEAF (CLF), SWINGER (SWN), MEDEA (MEA), EMBRYONIC FLOWER 2 (EMF2) and FERTILISATION INDEPENDENT ENDOSPERM (FIE) regulate developmental programmes by depositing H3K27me3 on thousands of target genes (Zhang et al. 2007; Lafos et al. 2011; Deleris et al. 2012). In addition, the homeodomain protein LIKE HETEROCHROMATIN PROTEIN 1 (LHP1) reinforces PRC2 function by binding to H3K27me3‐marked chromatin (Baile et al. 2021; Turck et al. 2007; Exner et al. 2009; Veluchamy et al. 2016).

Although PcG‐mediated repression is essential for plant development, recent studies have highlighted dynamic histone modifications during plant responses to wounding (Zhao et al. 2021). In *Arabidopsis*, wound‐induced transcriptional reprogramming is strongly associated with changes in histone acetylation. Genome‐wide analyses revealed that histone acetylation marks such as H3K9/14ac and H3K27ac increase at promoters of wound‐inducible genes, correlating with rapid transcriptional activation (Rymen et al. 2019). Besides histone acetylation, Histone methylation also contributes to wound responses (Rymen et al. 2019; Zhao et al. 2021). For example, H3K4me3, a mark associated with active transcription, is enriched at promoters of wound‐inducible genes, though it often appears after acetylation and transcription initiation, suggesting a reinforcing role. By contrast, the repressive mark H3K27me3 is not consistently removed at activated wound genes, indicating that transcription can still proceed in the presence of polycomb‐mediated repression. Furthermore, crosstalk between histone modifications and jasmonate signalling, mediated in part by histone deacetylases such as HDA6, underscores the complex balance between growth, defence, and regeneration (Vincent et al. 2022).

Accumulating evidence suggests that histone modifications play crucial roles not only in developmental gene regulation but also in defence responses in plants (Kinoshita and Seki 2014; Probst and Mittelsten Scheid 2015; Espinas et al. 2016; Lämke and Bäurle 2017; Ramirez‐Prado et al. 2018). However, whether epigenetic control directly regulates stress‐responsive secondary metabolism remains less understood. In this study, we performed time‐course transcriptomic, epigenomic, and metabolic analyses to uncover the molecular mechanisms underlying the regulation of glucosinolate (GSL) metabolism in *Arabidopsis* under stress. Measurement of GSL contents revealed a significant increase in GSL levels in the *lhp1‐4* mutant. Consistently, GSL pathway genes were strongly upregulated in *lhp1‐4*, whereas transcript levels returned to wild‐type levels in LHP1‐complemented plants. ChIP‐seq analysis further demonstrated that many GSL pathway genes are enriched with H3K27me3, while histone acetylation (H3ac) was particularly enriched at indolic GSL genes, suggesting a bivalent chromatin state that may enable rapid stress responses. Our study revealed that bivalent epigenetic histone modifications enable dynamic regulation of GSL genes, thereby mediating GSL metabolism in Arabidopsis exposed to wounding.

## 2 Results

### 2.1 Epigenetic Suppression of Glucosinolate Metabolism by LHP1‐Associated Polycomb Repression

**Figure 2:**
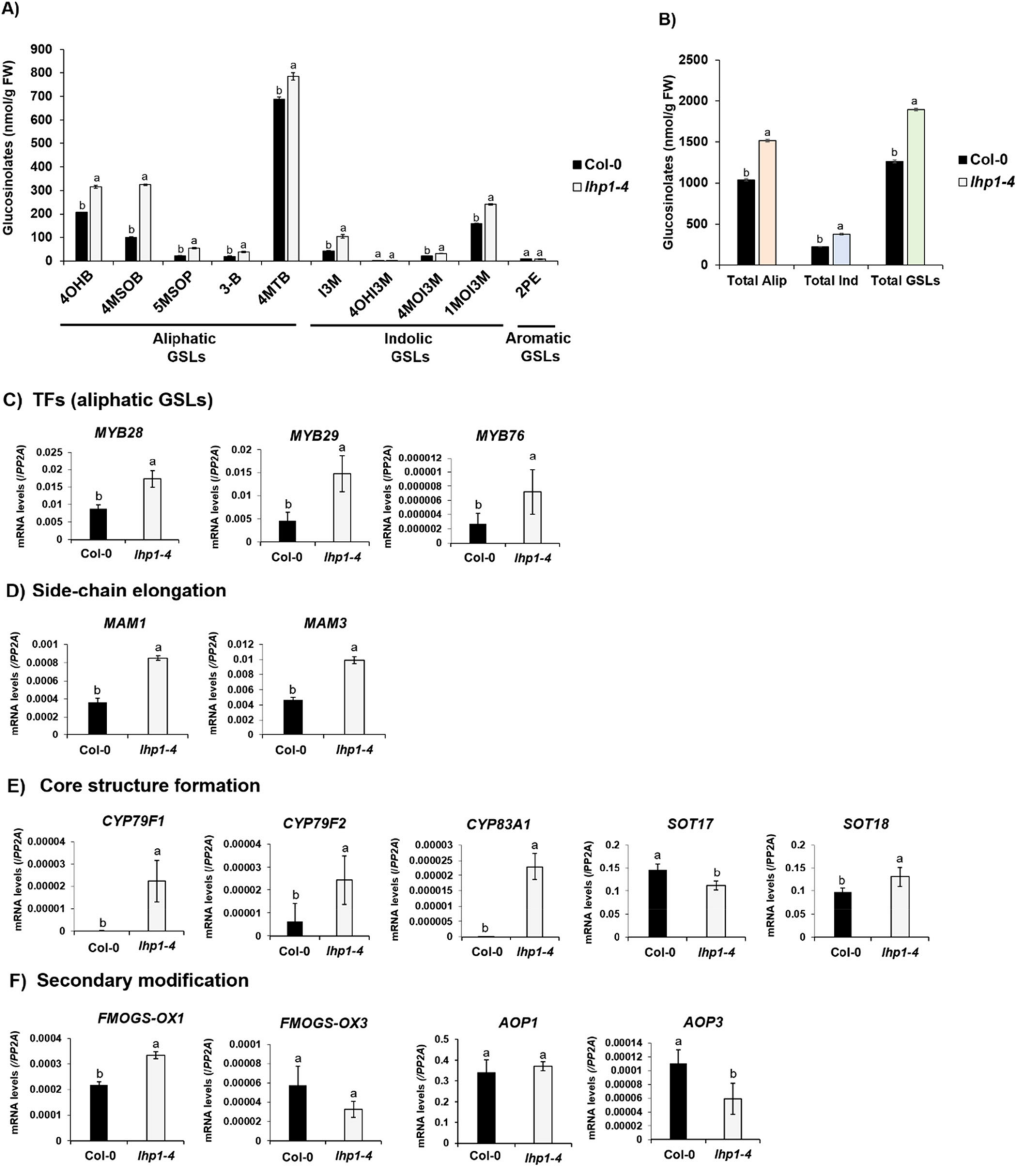
Increased amounts of GSLs in a polycomb mutant, *lhp1‐4*. (A) Detection of aliphatic, indolic and aromatic GSLs between Col‐0 and a polycomb mutant, *lhp1‐4*. Five aliphatic GSL compounds (4OHB, 4MSOB, 5MSOP, 3‐B and 4MTB), four indolic GSL compounds (I3M, 4OHI3M, 4MOI3M and 1MOI3M), and one aromatic GSL compound (2PE) were detected. (B) Comparison of total aliphatic (Alp) GSLs, total indolic (Ind) GSLs and total GSL amounts between Col‐0 and a polycomb mutant, *lhp1‐4*. The *lhp1‐4* mutants exhibited increased levels of aliphatic and indolic GSL compounds compared to those of Col‐0. (C) Result of qRT‐PCR analysis on three *MYB* TF (*MYB28, MYB29* and *MYB76*) genes involved in the aliphatic GSLs biosynthesis between Col‐0 and a polycomb mutant, *lhp1‐4*. (D) Result of qRT‐PCR on two ‘side‐chain elongation’ phase genes (*MAM1* and *MAM3*) involved in the aliphatic GSLs biosynthesis between Col‐0 and a polycomb mutant, *lhp1‐4*. (E) Result of qRT‐PCR on five ‘core structure formation’ phase genes (*CYP79F1, CYP79F2, CYP83A1, SOT17* and *SOT18*) involved in the aliphatic GSLs biosynthesis between Col‐0 and a polycomb mutant, *lhp1‐4*. (F) Result of qRT‐PCR on four ‘secondary modification’ phase genes (*FMO GS‐OX1, FMO GS‐OX3* and *AOP3*) involved in the aliphatic GSLs biosynthesis between Col‐0 and a polycomb mutant, *lhp1‐4*. Significance was statistically determined using one‐way analysis of variance (ANOVA) and Tukey's post‐hoc test (*p* < 0.05) and indicated with different letters above the line. [Color figure can be viewed at wileyonlinelibrary.com]

Epigenetic transcriptional reprogramming plays a crucial role in not only plant developmental programmes but also metabolic changes in plants (Ahmad et al. 2010; Nugroho et al. 2023). In particular, we aimed to test whether histone modifiers are involved in the control of the GSL biosynthesis pathway and selected a representative repressive complex, PRC2. LHP1 play an essential component of the PRC2 complex, which directly binds to H3K27me3 marks and stably suppresses target gene expression in cooperation with the core PRC2 complex components (Turck et al. 2007). Thus, we obtained the corresponding a loss‐of‐function mutant, *lhp1‐4*, and measured levels of GSLs between Col‐0 (wild‐type) and *lhp1‐4* using the Ultrahigh‐Performance Liquid Chromatography (UHPLC) method. In our detection condition, five aliphatic (4OHB, 4MSOB, 5MSOP, 3‐B and 4MTB), four indolic (I3M, 4OHI3M, 4MOI3M and 1MOI3M), and one aromatic GSL (2PE) compounds were identified in all genotypes, although levels of 2PE were minimal (Figure 2A). Among aliphatic GSL compounds detected, 4MTB was at the highest level followed by 4OHB and 4MSOB. The total amount of aliphatic GSL compounds was substantially higher than one of indolic GSL compounds (Figure 2B). It indicates that aliphatic GSL compounds are predominantly synthesised in Arabidopsis seedlings and relatively lower amounts of indolic GSLs.

We compared the endogenous levels of GSL compounds between Col‐0 and *lhp1‐4* mutant. Notably, the levels of all five aliphatic GSL compounds were significantly elevated in *lhp1‐4* mutant compared to those of Col‐0 (Figure 2A). It indicated that PRC2 complex is required for the suppression of aliphatic GSL production. Next, in case of indolic GSL compounds, three GSL compounds such as I3M, 4MOI3M and 1MOI3M significantly increased in *lhp1‐4* mutant compared to those of Col‐0 (Figure 2A). However, one aromatic GSL (2PE) compound did not exhibit a significant change between Col‐0 and *lhp1‐4* mutant (Figure 2A). Collectively, the *lhp1‐4* mutant had significantly higher levels of both aliphatic and indolic GSL compounds when compared to those of Col‐0 in our detection system (Figure 2B), indicating that that the PRC2 complex takes part in the process of GSL metabolism.

### 2.2 Upregulation of MYB28/29/76 in *lhp1‐4* Mutants Correlates With Enhanced Aliphatic Glucosinolate Production

Because amounts of GSL compounds were substantially elevated in the *lhp1‐4* mutant (Figure 2A,B), we next examined whether GSL biosynthetic pathway genes are transcriptionally regulated by LHP by performing quantitative reverse transcription PCR (qRT‐PCR) between Col‐0 and *lhp1‐4* mutant. The gene regulatory networks underlying the GSL biosynthetic pathway are mainly coordinated by several MYB TF genes (Hirai et al. 2007; Frerigmann and Gigolashvili 2014). For instance, MYB28, MYB29 and MYB76 are involved in aliphatic GSL biosynthesis, whereas MYB34, MYB51, MYB122 and OBP2 participate in the regulation of indolic GSL production. For aliphatic GSL, *MYB28, MYB29* and *MYB76* genes were significantly upregulated in the *lhp1‐4* mutant compared to those of Col‐0 (Figure 2C). It suggested that the increased expression of genes encoding the MYB TFs could lead to the increase of aliphatic GSL compounds in the *lhp1*‐*4* mutant relative to Col‐0. In case of indolic GSL pathway, expression of *MYB34* was moderately increased in the *lhp1‐4* mutants compared to those of Col‐0, whereas *MYB122* did not show significant change compared to those of Col‐0 (Supporting Information Figure S1A). Transcript level of *MYB51* was rather downregulated in *lhp1‐4* mutant compared to the level of Col‐0 (Supporting Information Figure S1A), in line with a previous study that showed the expression pattern of *MYB34* opposite to one of *MYB51* (Frerigmann et al. 2016). Thus, it is likely that even if MYB34 and MYB51 are specified for the regulation of indolic GSLs, their regulatory contribution to defence responses might be diverged, depending on the types of stress (i.e., wounding, pathogen, and herbivore), tissues, and developmental stages in Arabidopsis. This speculation needs further clarification.

### 2.3 Aliphatic GSL Pathway Genes are Broadly Upregulated in *lhp1‐4*, While Indolic Pathway Regulation Remains Complex

Upregulation of genes encoding TFs (*MYB28, MYB29* and *MYB76*) associated with the aliphatic GSL pathway (Figure 2C) led us to hypothesise that the regulatory networks mediated by these TFs coordinate transcriptional reprogramming underlying aliphatic production. To test it, we performed qRT‐PCR analysis between Col‐0 and *lhp1‐4* mutant with 11 genes randomly selected from total 23 aliphatic GSL biosynthetic genes comprising three different phases of aliphatic GSL biosynthesis (Figure 2D–F). It included two ‘side‐chain elongation’ phase genes (*MAM1* and *MAM3*), five ‘core structure formation’ phase genes (*CYP79F1*, *CYP79F2*, *CYP83A1*, *SOT17/ST5c* and *SOT18/ST5b*), and four ‘secondary modification’ phase genes (*FMOGS‐OX1*, *FMOGS‐OX3*, *AOP1* and *AOP3*). We identified prominent upregulation of genes belonging to the ‘side‐chain elongation’ phase (*MAM1* and *MAM3*) and the ‘core structure formation’ phase (*CYP79F1*, *CYP79F2*, *CYP83A1* and *SOT18*) in the *lhp1‐4* mutant compared to Col‐0, except for *SOT17* that did not show a significant change in the *lhp1‐4* mutant) Figure 2D–E). Among the genes involved in the ‘secondary modification,’ *FMOGS‐OX1* was only significantly upregulated in the *lhp1‐4* mutant, whereas *FMOGS‐OX3, AOP1* and *AOP3* genes did not show significant upregulations in the *lhp1‐4* mutant in comparison to those of Col‐0 (Figure 2F). Taken together, we concluded that the loss of functional LHP1, a component of PRC2 complex resulted in the substantial upregulation of aliphatic GSL pathway genes.

Next, we also conducted qRT‐PCR analysis between Col‐0 and *lhp1‐4* mutant with 11 genes randomly selected from total 12 indolic GSL pathway genes comprising two different phases of indolic GSL biosynthesis (Supporting Information Figure S1B–S1C). It included six genes involved in ‘core structure formation’ phase genes (*CYP79B2*, *CYP79B3*, *CYP83B1*, *GGP1*, *SUR1* and *SOT16*) and five genes associated with ‘secondary modification’ phase genes (*CYP81F2, CYP81F3, CYP81F4, IGMT1* and *IGMT2*). High portion of indolic GSL pathway genes were not substantially affected in the *lhp1‐4* mutant. For instance, five genes like *CYP79B3, SUR1, CYP81F4, IGMT1* and *IGMT2* were upregulated in the *lhp1‐4* mutant compared to those of Col‐0. Meanwhile, six other genes (*CYP79B2, CYP83B1, GGP1, SOT16*, *CYP81F2* and *CYP81F3*) remained largely unaffected or, in some cases, downregulated in the *lhp1‐4* mutant when compared to Col‐0 (Supporting Information Figure S1B and S1C). Regarding this observation, there might be a more complicated regulatory system in the indolic GSL pathway than that of aliphatic GSL pathway in *Arabidopsis*. Taken together, although the levels of indolic GSL compounds were elevated in *lhp1‐4* compared to Col‐0, the loss of functional PRC2 led to dynamic alterations in the expression of genes involved in the indolic GSL biosynthetic pathway. It might need further clarification.

### 2.4 Functional Complementation of *lhp1‐4* by *LHP1* Overexpression Restores GSL Levels and Gene Expression

**Figure 3:**
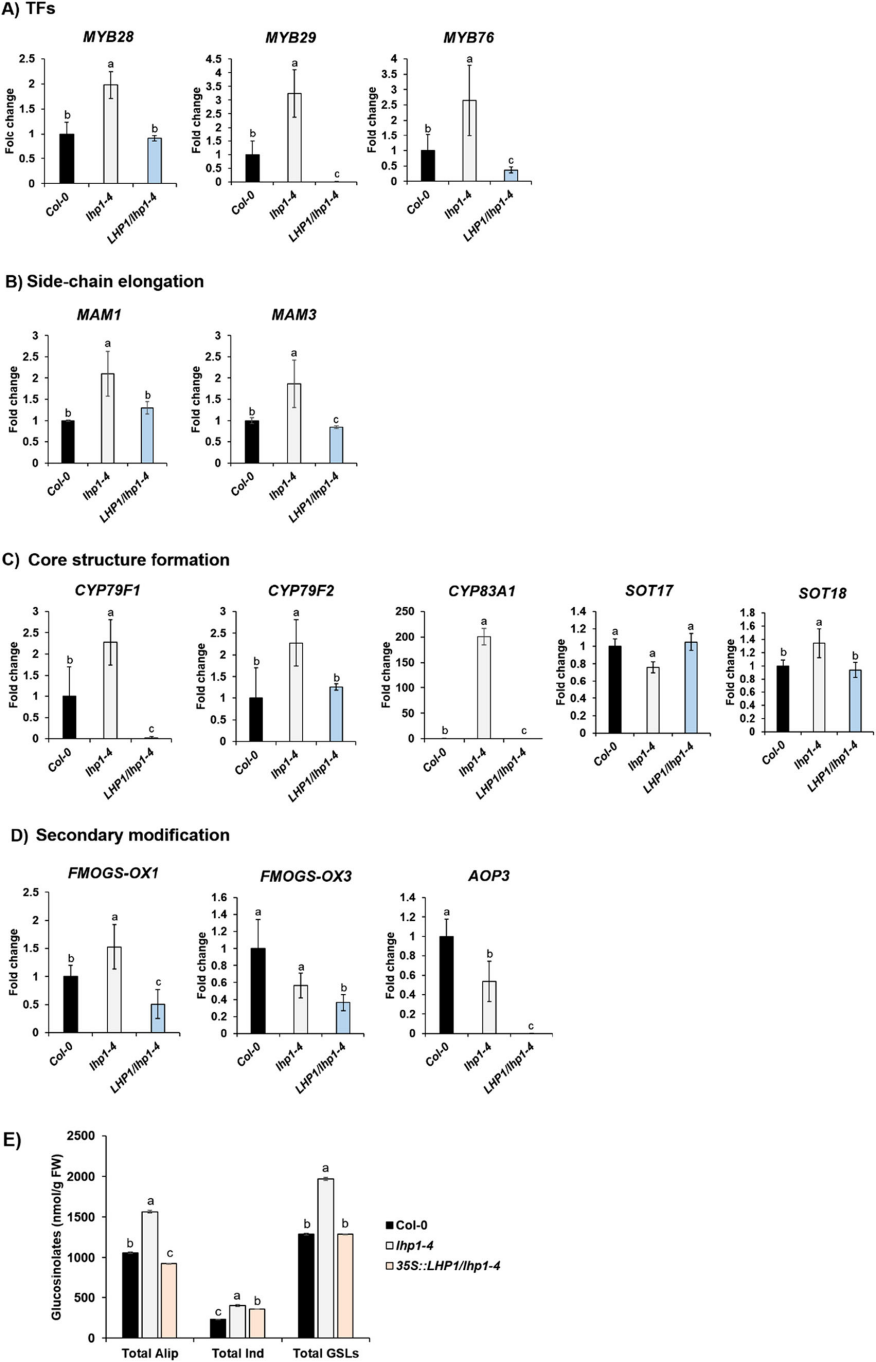
Result of qRT‐PCR analysis of genes involved in the aliphatic GSL pathway genes between Col‐0, *lhp1‐4* mutant, and *35S::LHP1/lhp1‐4* transgenic line. (A) Result of qRT‐PCR analysis of three MYB TF genes (*MYB28, MYB29* and *MYB76*) involved in the regulation of aliphatic GSL biosynthesis between Col‐0, *lhp1‐4* mutant, and *35S::LHP1/lhp1‐4* transgenic line. (B) Result of qRT‐PCR analysis of two ‘side‐chain elongation’ phase genes (*MAM1* and *MAM3*) between Col‐0, *lhp1‐4* mutant, and *35S::LHP1/lhp1‐4* transgenic line. (C) Result of qRT‐PCR analysis of five ‘core structure formation’ phase genes (*CYP79F1, CYP79F2, CYP83A1, SOT17* and *SOT18*) between Col‐0, *lhp1‐4* mutant, and *35S::LHP1/lhp1‐4* transgenic line. (D) Result of qRT‐PCR analysis of three ‘secondary modification’ phase genes (*FMOGS‐OX1, FMOGS‐OX3* and *AOP3*) between Col‐0, *lhp1‐4* mutant, and *35S::LHP1/lhp1‐4* transgenic line. (E) Measurement of GSL compounds between Col‐0, *lhp1‐4* mutant, and *35S::LHP1/lhp1‐4* transgenic line. While *lhp1‐4* mutants exhibited increased levels of aliphatic and indolic GSL compounds compared to those of Col‐0, *35S::LHP1/lhp1‐4* transgenic plant significantly restored the levels of aliphatic and indolic GSLs to the comparable levels of Col‐0 samples. Alip: total aliphatic GSL compounds, Ind: total indolic GSL compounds. Significance was statistically determined using one‐way analysis of variance (ANOVA) and Tukey's post‐hoc test (*p* < 0.05) and indicated with different letters above the line. [Color figure can be viewed at wileyonlinelibrary.com]

To confirm that loss of LHP1 function is cause of increased amounts of GSLs, we performed qRT‐PCR to measure transcript levels of GSL pathway genes between Col‐0 and the overexpressed transgenic line of *LHP1* in *the lhp1‐4* mutant background, *35S::LHP1/lhp1‐4* (Supporting Information Figure S2). As a result, most of alleviated genes in the *lhp1‐4* mutant were substantially suppressed in the *35S::LHP1/lhp1‐4* transgenic line (Figure 3A–D and Supporting Information Figure S3). These results confirmed that the biosynthesis of GSLs is suppressed by the epigenetic repressor PRC2 complex containing LHP1. Furthermore, we also measured amounts of GSL compounds between Col‐0 and the *35S::LHP1/lhp1‐4* transgenic line. The *35S::LHP1/lhp1‐4* transgenic line displayed significantly reduced levels of aliphatic and indolic GSL compounds compared to those of *lhp1‐4* mutant, comparable to those of Col‐0 (Figure 3E).

### 2.5 PRC2 Represses Aliphatic Glucosinolate Biosynthesis Through Direct H3K27me3 Deposition on MYB Regulators and Biosynthetic Genes

**Figure 4:**
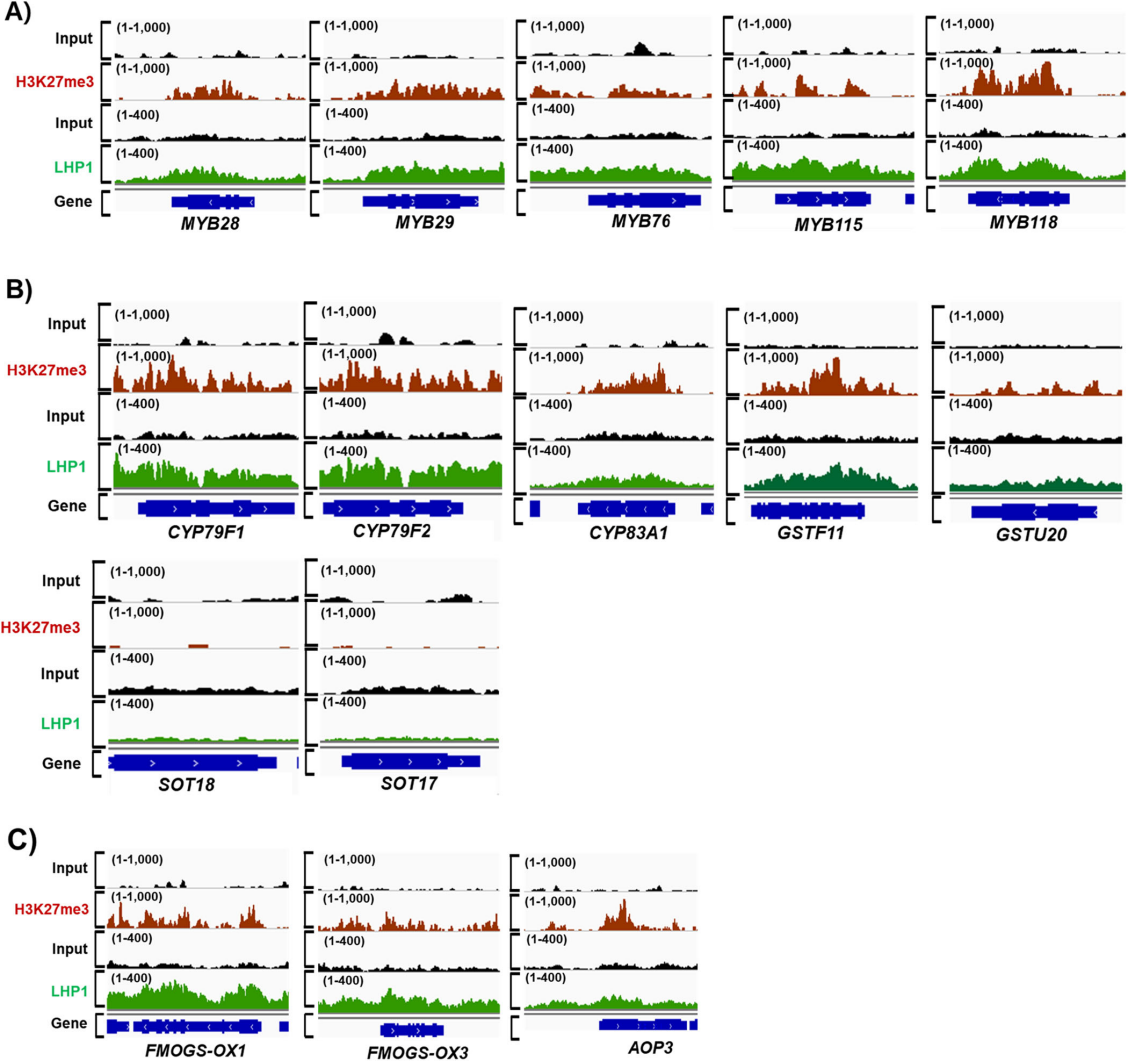
Genome browser view of H3K27me3 and LHP1 enrichment profile on aliphatic GSL pathway genes. (A) Aligned ChIP‐seq reads of H3K27me3 and LHP1 on five TF genes (*MYB28, MYB29, MYB76, MYB115* and *MYB118*) were presented with red and green colours, respectively. Aligned ChIP‐seq reads of Input DNA were presented with black colour. All TF genomic regions were shown to be heavily enriched with H3K27me3 and LHP1 when compared to those of input DNA. (B) Aligned ChIP‐seq reads of H3K27me3 and LHP1 on seven ‘core structure formation’ phase genes involved in the aliphatic GSL pathway were presented with red and green colours, respectively. Aligned ChIP‐seq reads of Input DNA were presented with black colour. Five genes (*CYP79F1, CYP79F2, CYP83A1, GSTF11* and *GSTU20*) were enriched with H3K27me3 and LHP1, whereas two genes like *SOT18* and *SOT17* were not enriched with both H3K27me3 and LHP1 in comparison to the level of input DNA. (C) Aligned ChIP‐seq reads of H3K27me3 and LHP1 on three ‘secondary modification’ phase genes involved in the aliphatic GSL pathway were presented with red and green colours, respectively. All three ‘secondary modification’ phase genes (*FMO GS‐OX1, FMO GS‐OX3* and *AOP3*) were enriched with H3K27me3 and LHP1 in comparison to the level of input DNA. Aligned ChIP‐seq reads of Input DNA were presented with black colour. (A~C) The *y*‐axis shows the number of normalised reads mapped to the annotated individual genes. ChIP‐seq data set showing H3K27me3 enrichment (red colour track) on GSL pathway genes were produced using anti‐H3K27me3 antibody (Upstate, USA, Cat. No: 07–449) in the 2‐week‐old seedlings of Col‐0. In addition, data set showing LHP1 enrichments (green colour track) on GSL pathway genes were generated with anti‐GFP antibody (Takara Biotech Clontech, Japan, Cat. No: 632592) using pLHP1::LHP1‐GFP transgenic line. Detailed information on the genome‐wide ChIP‐seq data set analysed in this study is provided in Supporting Information Table S3. [Color figure can be viewed at wileyonlinelibrary.com]

PRC2 complex catalysers the deposit of the repressive histone mark, H3K27me3 on target gene chromatin. Thus, we reasoned that PRC2 complex repress the transcription of the aliphatic and indolic GSL pathway genes by depositing H3K27me3 to the genomic loci of the MYB TF genes, triggering transcriptional signal cascades. To test this, we analysed an H3K27me3 ChIP‐seq profile to obtain the global landscape of H2K27me3 enrichment (Wang et al. 2016). Our analyses revealed that all five *MYB* TFs that function in the aliphatic GSL pathway showed strong enrichment of H3K27me3 (Figure 4A), indicating that LHP1 directly suppresses the expression of these MYB TFs via depositing H3K27me3. In addition to the MYB TF genes, among the seven ‘core structure formation’ genes, five genes (71%) (*CYP79F1, CYP79F2, CYP83A1, GSTF11* and *GSTU20*) were enriched with H3K27me3 (Figure 4B), confirming that PRC2 complex directly suppresses not only the expression of the MYB TF genes but also one of many ‘core structure formation’ genes by depositing H3K27me3. Meanwhile, *SOT17* and *SOT18* genic regions displayed little enrichment of H3K27me3 (Figure 4B), indicating they are unlikely to be directly regulated by PRC2‐mediated suppression.

Following ‘core structure formation’ phase, aliphatic GSL compounds undergo the ‘secondary modification’ on their side chains (Figure 1). Methylthioalkyl GSLs (e.g., 4MTB) is oxidised to methylsulfinylalkyl GSLs (e.g., 4MSOB), which is mediated by a subset of flavin monooxygenase enzymes, called FMO GS‐OX1 and FMO GS‐OX3 (Li et al. 2008). Methylsulfinylalkyl GSLs are then oxidised to alkenyl (e.g., 3‐B) or hydroxyalkyl GSLs (e.g., 4OHB), which is mediated by α–ketoglutarate‐dependent dioxygenases like AOP3 (Figure 1). We found that H3K27me3 was substantially enriched on the genic regions of all ‘secondary modification’ phase genes, *FMOGS‐OX1, FMOGS‐OX3* and *AOP3*, in aliphatic GSL pathway (Figure 4C). Even we observed that *FMOGS‐OX1* gene was upregulated in the *lhp1‐4* mutant compared to that of Col‐0, *FMOGS‐OX3* and *AOP3* genes were not substantially affected in the *lhp1‐4* compared to those of Col‐0. Thus, it needs further clarification on why these two genes were not influenced in the loss of functional PRC2 in *Arabidopsis*. Taken together, these data demonstrated that a majority of the ‘core function formation’ and some of ‘secondary modification’ phase genes are under the epigenetic repression mediated by the LHP1‐containing PRC2 complex in *Arabidopsis*.

### 2.6 PRC2‐Mediated H3K27me3 Represses TFs and Secondary Modification Genes in the Indolic GSL Pathway

**Figure 5:**
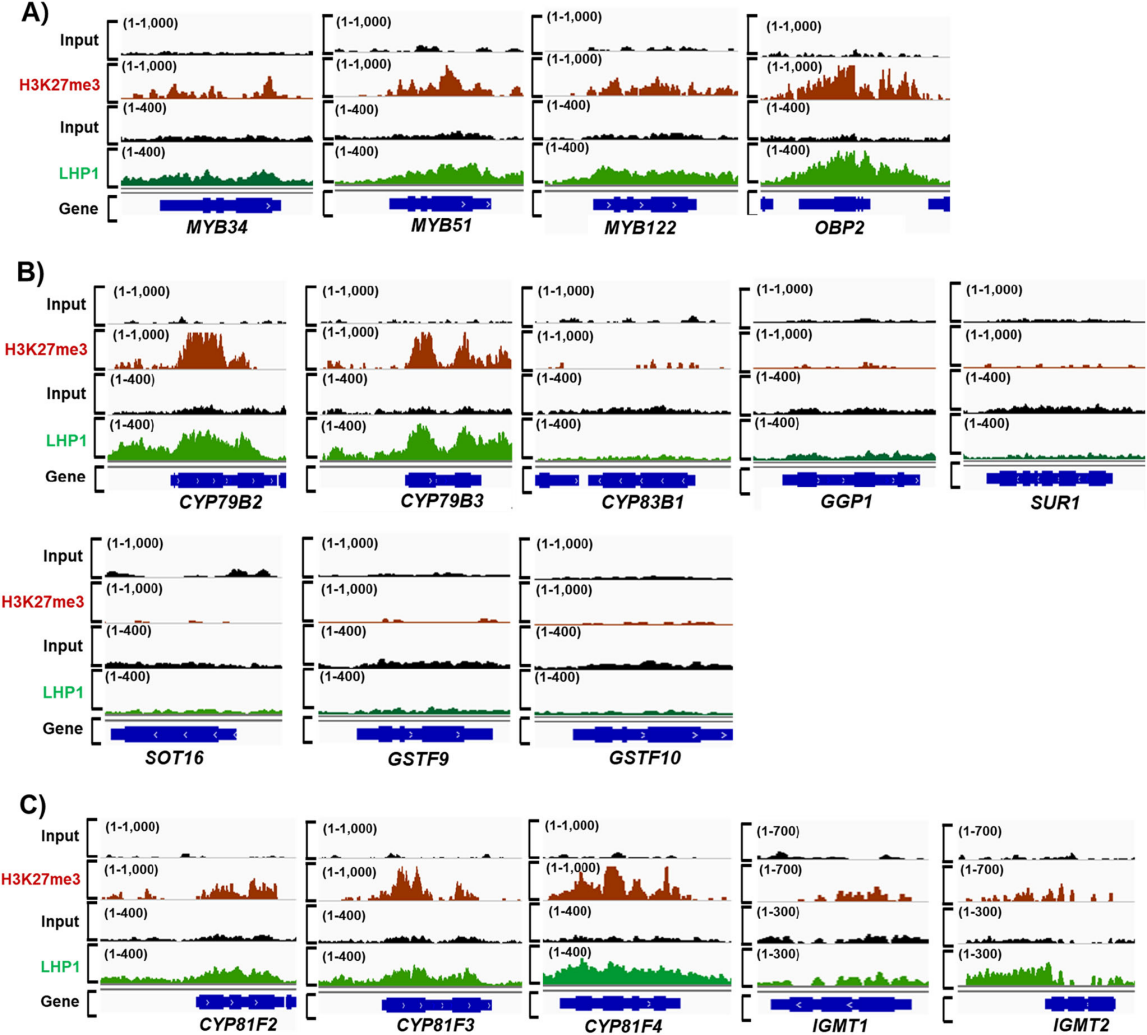
Genome browser view of H3K27me3 and LHP1 enrichment profiles on indolic GSL pathway genes. (A) Aligned ChIP‐seq reads of H3K27me3 and LHP1 on four TF genes (*MYB34, MYB51, MYB122* and *OBP2*) were presented with red and green colours, respectively. Aligned ChIP‐seq reads of Input DNA were presented with black colour. (B) Aligned ChIP‐seq reads of H3K27me3 and LHP1 on eight ‘core structure formation’ phase genes involved in the indolic GSL pathway were presented with red and green colours, respectively. Aligned ChIP‐seq reads of Input DNA were presented with black colour. Two genes (*CYP79B2 and CYP79B3*) were enriched with H3K27me3 and LHP1, whereas six genes (*CYP83B1, GGP1, SUR1, SOT16, GSTF9* and *GSTF10*) were not enriched with both H3K27me3 and LHP1 in comparison to the level of input DNA. (C) Aligned ChIP‐seq reads of H3K27me3 and LHP1 on five ‘secondary modification’ phase genes involved in the indolic GSL pathway were presented with red and green colours, respectively. All five ‘secondary modification’ phase genes (*CYP81F2, CYP81F3, CYP81F4, IGMT1* and *IGMT2*) were enriched with H3K27me3 and LHP1 in comparison to the level of input DNA. Aligned ChIP‐seq reads of Input DNA were presented with black colour. (A~C) The *y*‐axis shows the number of normalised reads mapped to the annotated individual genes. ChIP‐seq data set showing H3K27me3 enrichment (red colour track) on GSL pathway genes were produced using anti‐H3K27me3 antibody (Upstate, USA, Cat. No: 07–449) in the 2‐week‐old seedlings of Col‐0. In addition, data set showing LHP1 enrichments (green colour track) on GSL pathway genes were generated with anti‐GFP antibody (Takara Biotech Clontech, Japan, Cat. No: 632592) using pLHP1::LHP1‐GFP transgenic line. Detailed information on the genome‐wide ChIP‐seq data set used in this study is provided in Supporting Information Table S3. [Color figure can be viewed at wileyonlinelibrary.com]

Indolic GSL compounds undergo the ‘core structure formation’ and ‘secondary modification’ phase (Figure 1). Indol‐3‐ylmethyl GSL (i.e., I3M) is hydroxylated to 1‐hydroxyindole‐3‐ylmethyl GSL (i.e., 1OHI3M) or 4‐hydroxyindol‐3‐ylmethyl GSL (i.e., 4OHI3M) by the CYP81F2 ~ CYP81F4 enzymes (Pfalz et al. 2011). These hydroxyindole GSLs are further methylated to form methoxyindole GSLs, which are mediated by IGMT1 and IGMT2. Our H3K27me3 ChIP‐seq analysis showed that three *MYB* and *OBP2* TFs that function in the indolic GSL pathway enriched with H3K27me3 highly (Figure 5A), indicating that PRC2 directly suppresses the expression of these *MYB TF* genes via depositing H3K27me3. Among the eight ‘core structure formation’ genes in the indolic GSL pathway, only *CYP79B2* and *CYP79B3* were found to be substantially enriched with H3K27me3, whereas the genic regions of *CYP83B1, GGP1, SUR1, SOT16, GSTF9* and *GSTF10* genes had little H3K27me3 (Figure 5B). Next, we also examined H3K27me3 enrichment on genes involved in the ‘secondary modification’ in the indolic GSL pathway. We identified that all five genes (100%) such as *CYP81F2, CYP81F3, CYP81F4, IGMT1* and *IGMT2* were heavily enriched with H3K27me3 (Figure 5C). It indicated that ‘secondary modification’ phase genes in both aliphatic and indolic GSL pathways are heavily suppressed by the PRC2 complex in Arabidopsis. Taken together, H3K27me3 profiling confirmed that the expression of both aliphatic and indolic GSL pathway genes, especially ‘TFs’ and ‘secondary modification’ phase genes, are likely to be tightly controlled by PRC2‐mediated H3K27me3 (Figure 6A and Supporting Information Figure S4).

Figure 6Enrichment of repressive histone marks on TFs and metabolic genes belonging to three biosynthetic phases for GSL production. (A) Bar graph showing percentages of H3K27me3, LHP1, and H3K9me2 histone mark on 9 TFs and metabolic genes involved in the three biosynthetic phases (10 ‘side‐chain elongation’ genes, 17 ‘core structure formation’ genes, and 10 ‘secondary modification’ genes) for the aliphatic and indolic GSL production in Arabidopsis. All 9 TF groups and 10 ‘secondary modification’ phage genes are deposited with H3K27me3, whereas 10 ‘side‐chain elongation’ and 17 ‘core structure formation’ phage genes were lowly deposited with H3K27me3. (B) Normalised H3K27me3 enrichments between Col‐0 wild type and *clf−29; swn‐4* (*clf; swn*), a double knock‐out mutant of CLF and SWN were presented in genomic IGV browser. A repressive histone mark, H3K27me3 were enriched at nine TFs controlling biosynthesis of aliphatic and indolic GSLs in Col‐0 wild type (red colour). Meanwhile, a *clf; swn* mutant showed severe reductions of H3K27me3 (skyblue colour) compared to level of Col‐0. Tracks for input DNA were indicated with black colour. Read coverage normalised using the total number of mapped reads are indicated at the top right corner of each track in the bracket. (C) Result of ChIP‐qPCR analysis on H3K27me3 enrichment of 12 randomly selected aliphatic or indolic GSL pathway genes. Upper panel: A schematic diagram showing the genomic structure of each aliphatic GSL pathway gene and relative positions of amplicons (presented with PX, X is Arabic number) used in the ChIP‐qPCR assay. Transcription start site was indicated with arrow. Bottom panel: Result of ChIP‐qPCR analysis. (D) Normalised H3K27me3 enrichments of nine TF genes between Col‐0 and *lhp1‐4*, a knock‐out mutant of LHP1 were presented in genomic IGV browser. A repressive histone mark, H3K27me3 were enriched at nine TFs controlling biosynthesis of aliphatic and indolic GSLs in Col‐0 wild type (purple colour). Meanwhile, a *lhp1‐4* mutant showed severe reductions of H3K27me3 (dark brown colour) compared to level of Col‐0. Tracks for input DNA were indicated with black colour. Read coverage normalised using the total number of mapped reads are indicated at the top right corner of each track in the bracket. (B and D) Information on the public ChIP‐seq data set on enriched levels of H3K27me3 between Col‐0 and a double mutant, *clf; swn* or *lhp1‐4* mutant was described in the Supporting Information Table S3. [Color figure can be viewed at wileyonlinelibrary.com]

**Figure:**
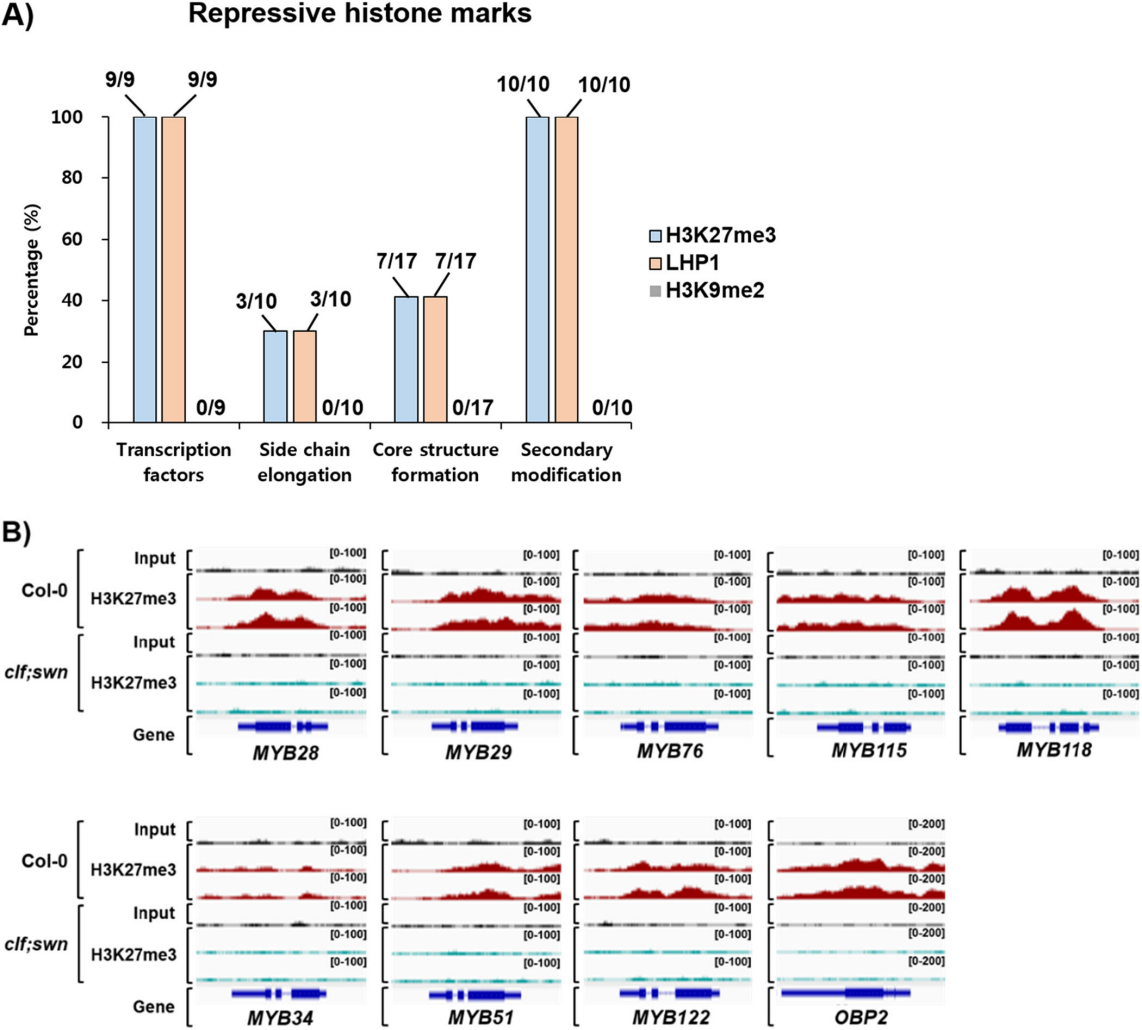

**Figure:**
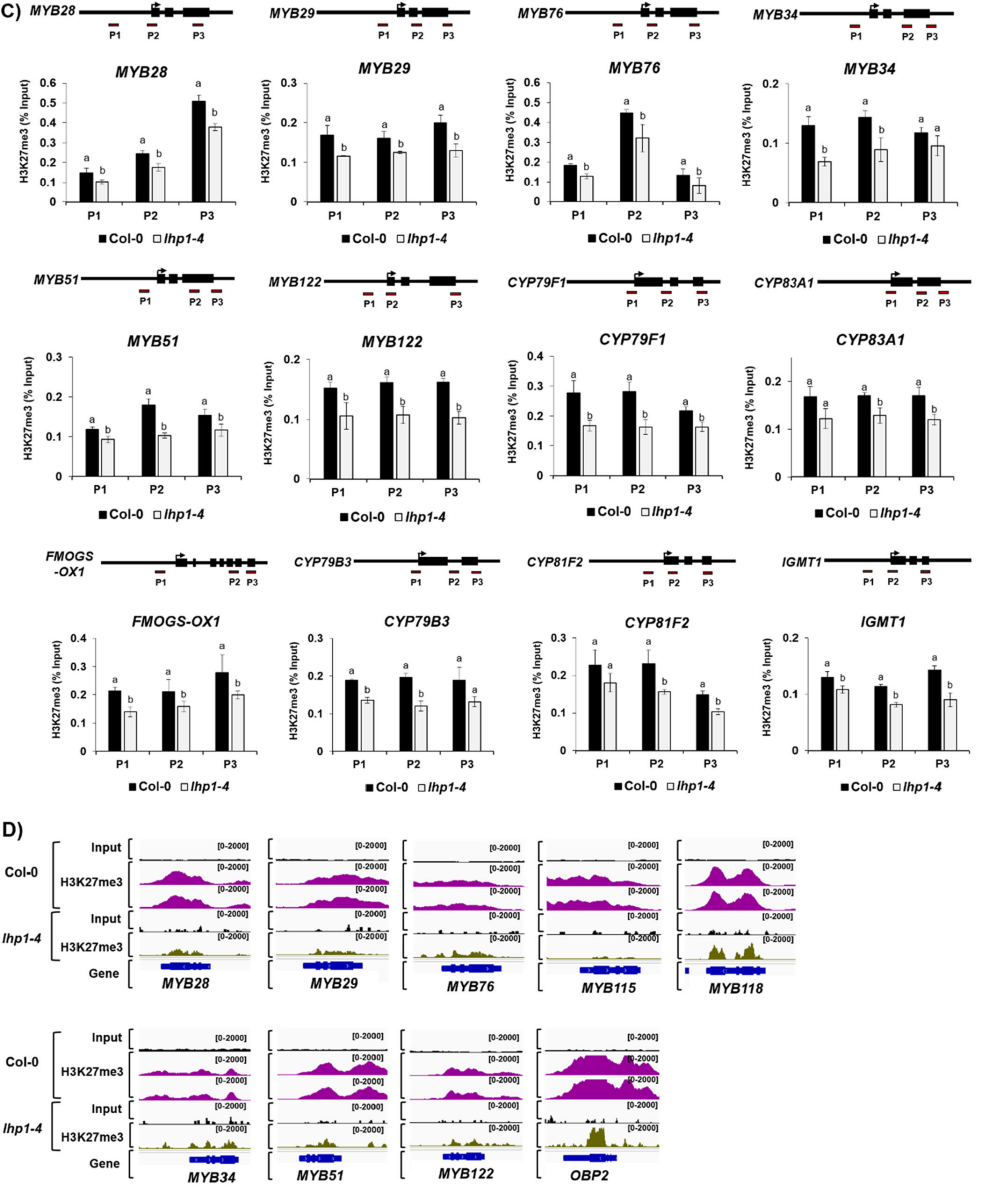

### 2.7 Stable Repression of GSL Biosynthetic Genes Requires PRC2‐Mediated H3K27me3 Deposition

To confirm that LHP1 is an essential component for the stable suppression of target GSL pathway genes, we also analysed a global DNA‐binding profile of LHP1 (Veluchamy et al. 2016; Turck et al. 2007; Baile et al. 2021). Most of the GSL pathway genes whose expression was elevated in the *lhp1‐4* mutant were enriched with LHP1 binding signals compared to the corresponding input signal intensity (Figures 4, 5, 6, and Supporting Information Figure S5). Thus, it is likely that the PRC2 complex‐mediated suppression participate in the epigenetic suppression of GSL pathway genes related to aliphatic and indolic GSL production.

PRC2 complex is required for deposition and stable maintenance of H3K27me3 to target genes (Turck et al. 2007; Laugesen et al. 2019). We postulated that loss of *CLF* and/or *SWN*, two major H3K27 methyltransferase genes in PRC2 complex might result in defects in the maintenance of H3K27me3 on PRC2‐targeted GSL pathway genes. Thus, we analysed the H3K27me3 occupancy between Col‐0 and *clf; swn* mutants background (Shu et al. 2019). GSL pathway genes enriched with H3K27me3 in Col‐0 exhibited severe depletion of H3K27me3 in the *clf; swn* mutant (Figure 6B and Supporting Information Figure S6).

Besides CLF and SWN, core components of PRC2 complex, we also tested whether another PRC2 component, LHP1 influences H3K27me3 enrichment on GSL pathway genes. ChIP‐qPCR analysis between Col‐0 and the *lhp1‐4* mutant was performed using H3K27me3 antibody (Figure 6C). The result showed that H3K27me3 enrichments at the tested genes were significantly reduced in *lhp1‐4* compared with those of Col‐0. Furthermore, we analysed a publicly available ChIP‐seq data set comparing H3K27me3 levels between Col‐0 and *lhp1‐4* (Figure 6D and Supporting Information Figure S7). Similar to the results shown in the *clf; swn* mutant relative to Col‐0, H3K27me3 enrichments at many GSL pathway genes were substantially reduced in the *lhp1‐4* mutant. These results confirmed that LHP1 is required for the proper enrichment of H3K27me3 on GSL pathway genes in Arabidopsis. Taken together, these results support our finding showing that PRC2 is required for the enrichment of H3K27em3 on GSL pathway genes.

### 2.8 GSL Pathway Genes are Regulated by Both Repressive and Active Histone Modifications

Epigenetic gene suppression is a coordinated action associated with not only H3K27me3 but also other histone methylations including H3K9me2 which is highly accumulated in the DNA methylated genomic contexts like transposable elements and repetitive sequences (Mathieu et al. 2005; Pikaard and Mittelsten Scheid 2014). To test whether H3K9me2 is involved in the suppression of GSL pathway genes, we analysed its global enrichment patterns (Zhao et al. 2022), identifying that none of the 46 GSL pathway genes showed enrichment with H3K9me2 (Figure 6A and Supporting Information Figure S8A ~ S8B). This suggests that H3K9me2‐mediated suppression does not act on the transcriptional regulation of GSL pathway genes.

Contrary to repressive H3K27me3 and H3K9me2 marks, some histone marks are correlated with gene activation (Ueda and Seki 2020). They include some methylation and acetylation at the certain residue of histone such as H3K4me3, H3K36me3 and H3ac. To check whether active histone modification(s) are involved in the transcription of GSL pathway genes, we analysed global profiles of multiple histone activation marks, H3 histone methylations (H3K4me3 and H3K36me3) and H3 acetylations (H3K9ac, H3K14ac, H3K23ac, and H3K36ac) (PRJNA305274 & PRJNA301697) (Figure 7A–G and Supporting Information Figures S9–S10). The H3K4me3 mark was most highly enriched in genes involved in the ‘Core structure formation’ phase (12/17, 71%), and to a lesser extent in those associated with ‘Side chain elongation’ (4/10, 40%) and ‘Transcription Factor (TF)’ genes (2/9, 22%) (Figure 7A and Supporting Information Figure S9). In contrast, the H3K4me3 mark was not detected in genes related to the ‘Secondary modification’ phase (0/10, 0%). H3K36me3 mark was only detected in the ‘Side chain elongation’ (4/10, 40%) and ‘Core structure formation’ phase genes (9/17, 53%) (Figure 7A and Supporting Information Figure S9). H3ac marks were detected across all gene groups, with enrichment observed in 22% (2/9) of ‘TF genes’, 40% (4/10) of ‘Side chain elongation’, 76% (13/17) of ‘Core structure formation’, and 40% (4/10) of ‘Secondary modification’ phase genes (Figure 7A and Supporting Information Figure S10). In the case of the H2Bub1 mark, it was detected at low levels only in the ‘Side chain elongation’ (3/10, 30%) and ‘Core structure formation’ (2/17, 12%) phase genes, with no detection in the ‘TF genes’ or ‘Secondary modification’ phase genes. (Figure 7A). It indicated that H2Bub1 is not be a major contributor in the activation of GSL pathway genes.

Figure 7Enrichment of active histone marks on genes involved in the aliphatic and indolic GSL biosynthesis. (A) A bar graph showing percentages of active histone mark enrichment like H3K4me3, H3K36me3, H3ac and H2Bub1 on 9 TFs, 10 ‘side‐chain elongation’ phase genes, 17 ‘core structure formation’ phase genes, and 10 ‘secondary modification’ phase genes involved in the aliphatic and indolic GSL biosynthesis of Arabidopsis. (B ~ D) Genomic browser view showing profile of H3ac marks on ‘side‐chain elongation’ phase genes (B), ‘core structure formation’ genes (C), and ‘secondary modification’ phase genes (D) involved in the aliphatic GSL biosynthesis. Read coverage normalised using a total number of mapped reads was indicated at the top left or right corner of each track in parenthesis. (E ~ F) Genomic browser view showing profile of H3ac marks on ‘core structure formation’ genes (E), and ‘secondary modification’ phase genes (F) involved in the indolic GSL biosynthesis. Read coverage normalised using a total number of mapped reads was indicated at the top left or right corner of each track in parenthesis. Compared to aliphatic GSL pathway genes (B ~ D), a higher portion of indolic GSL pathway genes are pre‐occupied with H3ac histone marks (E~F). (B~F) Significant accumulation of H3 acetylated histone marks on individual gene was indicated with skyblue colour box. (G) Mean value of RNA‐seq transcripts on each group containing 9 TFs, 10 ‘side‐chain elongation’ phase genes, 17 ‘core structure formation’ phase genes, and 8 ‘secondary modification’ phase genes. ‘Core structure formation’ and to a lesser degree, ‘side‐chain elongation’ phase genes are highly expressed among four groups involved in the GSL biosynthesis. This pattern resembles the percentage profile of active histone marks shown in A), suggesting that active histone mark enrichment contributes to the active expression of ‘core structure formation’ and ‘side‐chain elongation’ phase genes. (H) Normalised transcript reads of TFs, side‐chain elongation’, ‘core structure formation’ and ‘secondary modification’ phase genes involved in the aliphatic and indolic GSL biosynthesis. Particularly, ‘core structure formation’ and ‘secondary modification’ genes related to indolic GSL biosynthesis are more actively expressed than those of aliphatic GSL pathway genes. [Color figure can be viewed at wileyonlinelibrary.com]

**Figure:**
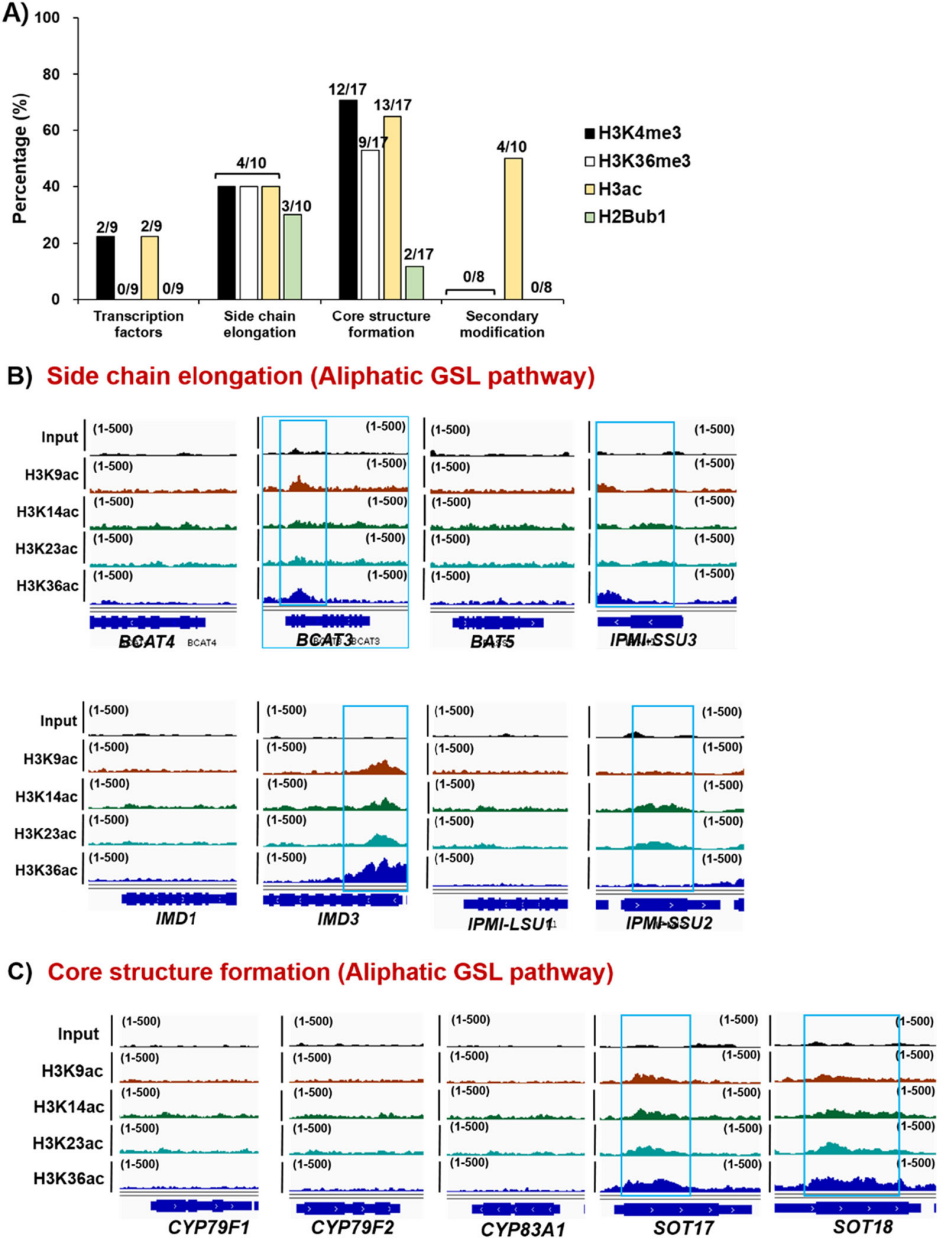

**Figure:**
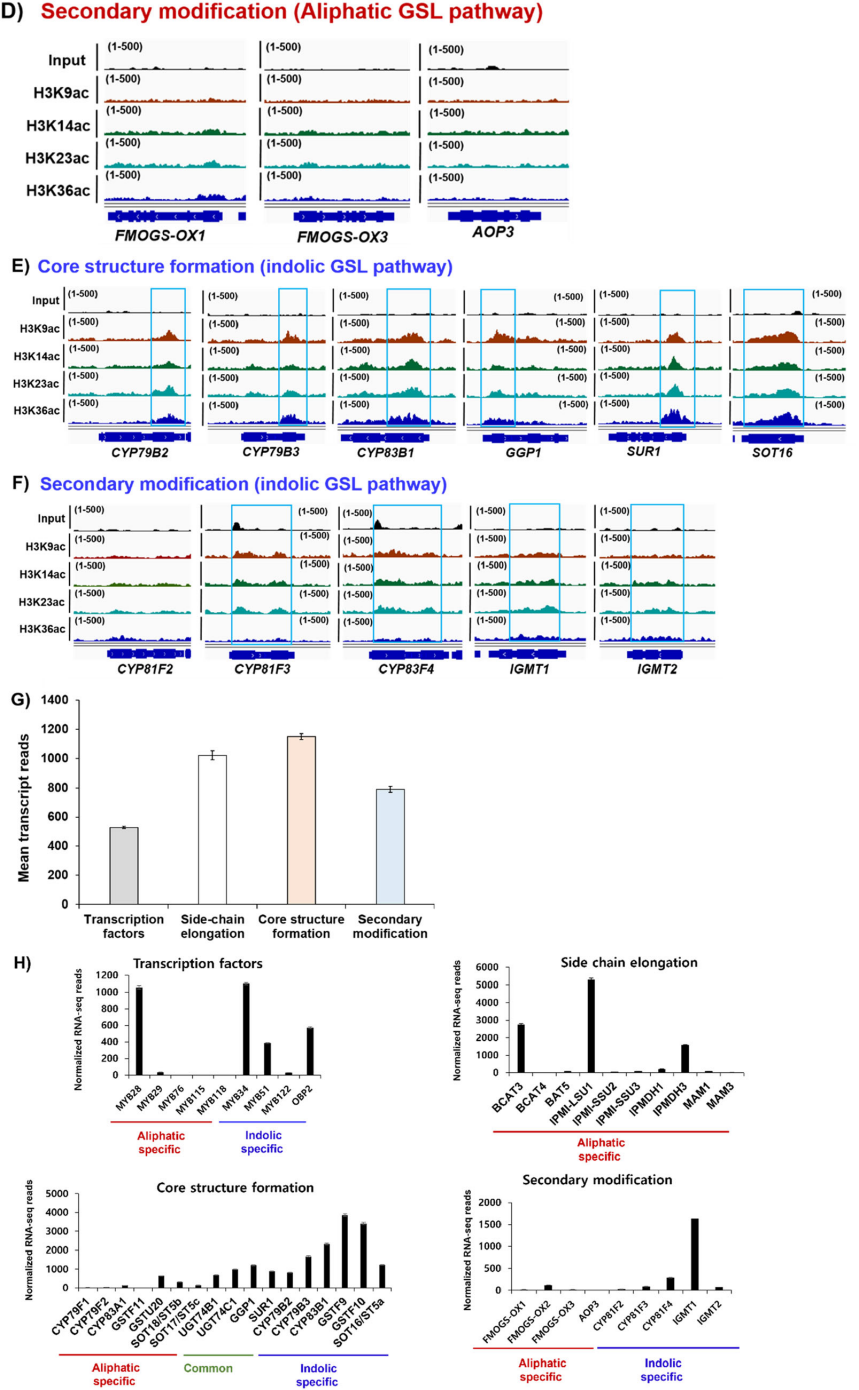

We noticed that the ‘core structure formation’ phase genes (76%, 13/17) were shown to have highest percentage of H3ac‐enriched genes when compared to other group of genes like ‘transcription factors’ (22%, 2/9) and ‘side‐chain elongation’ (40%, 4/10), and ‘secondary modification’ (40%, 4/10) (Supporting Information Figure S10). Interestingly, ‘core structure formation’ phase genes exhibited the low enrichment (41%, 7/17) of H3K27me3 among GSL pathway genes. These data indicated that ‘core structure formation’ phase genes are most highly enriched with active marks, H3ac and lowly occupied with a repressive mark, H3K27me3. Thus, we investigated whether an H3ac‐enriched and H3K27me3‐depleted chromatin context influences the expression of ‘core structure formation’ phase genes by analysing RNA‐seq data of Col‐0. As a result, the ‘core structure formation’ genes showed the highest mean transcript levels compared to genes from other phases, potentially reflecting the impact of their chromatin context (Figure 7G). Furthermore, when comparing H3ac enrichment between aliphatic and indolic GSL genes within the ‘core structure formation’ phase, a large proportion of indolic GSL genes exhibited significantly higher transcript levels than their aliphatic counterparts (Figure 7H). Collectively, these findings suggest that GSL pathway genes are regulated not only by repressive histone modifications but also by activating ones.

### 2.9 Bivalent Chromatin Marks are Highly Enriched in Indolic but not Aliphatic GSL Pathway Genes

**Figure 8:**
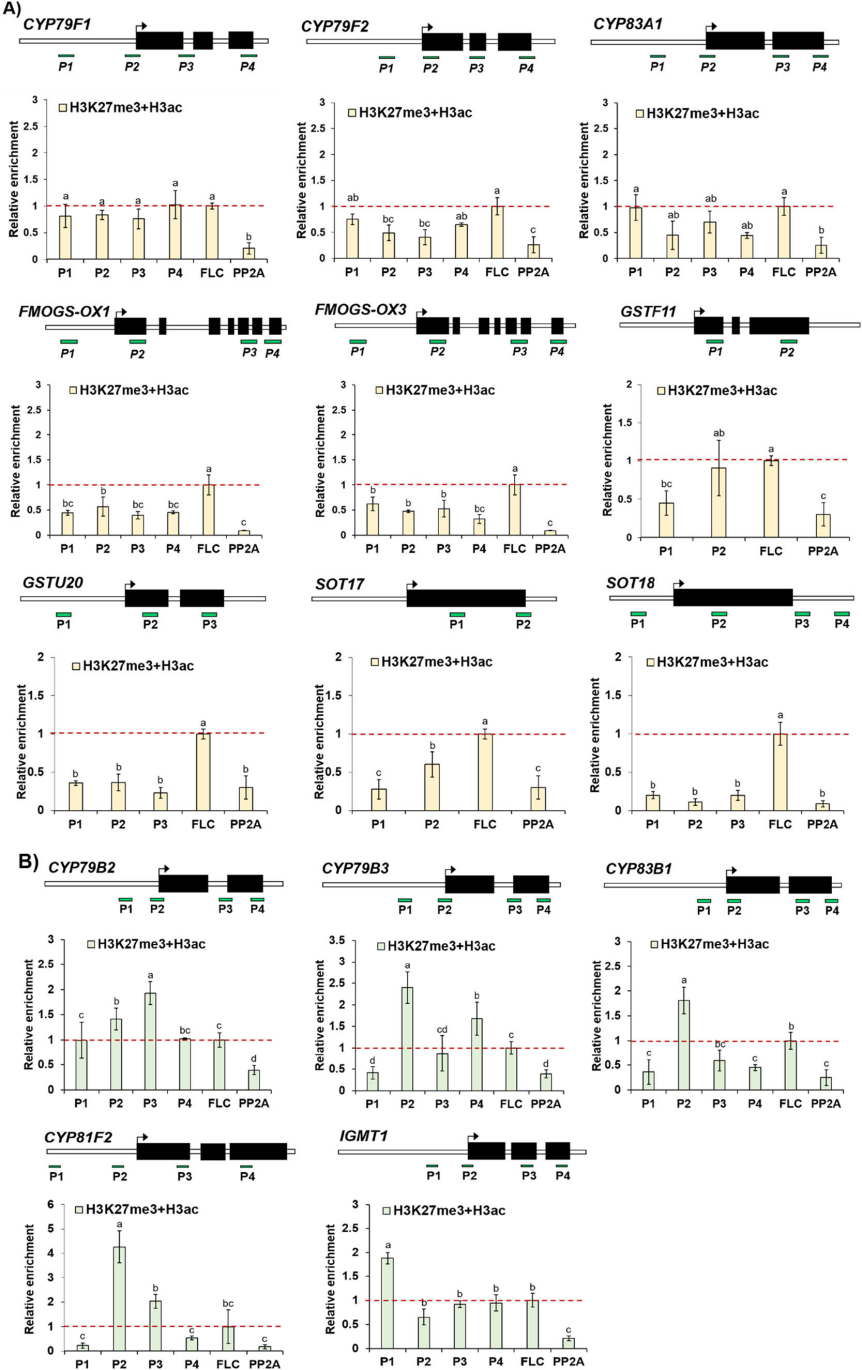
Results of sequential ChIP‐qPCR analysis on aliphatic and indolic GSL pathway genes, which was conducted by first ChIP using an anti‐H3K27me3 antibody followed by second ChIP using an anti‐H3ac antibody. (A) Results of sequential ChIP‐qPCR analysis using anti‐H3K27me3 and H3ac antibody on nine aliphatic GSL pathway genes. (B) Results of sequential ChIP‐qPCR analysis using anti‐H3K27me3 and H3ac antibody on five indolic GSL pathway genes. Upper panel: A schematic diagram showing the genomic structure of each aliphatic GSL pathway gene and relative positions of amplicons (presented with PX, X is Arabic number) used in the ChIP‐qPCR assay. Transcription start site was indicated with arrow. Bottom panel: Result of sequential ChIP‐qPCR analysis. The amplicon‐amplifying proximal promoter region of *FLC* (AT5G10140), a positive reference gene for bivalent histone marks and a negative reference gene *PP2A* (AT1G13320) were also used in the ChIP‐qPCR analysis for comparison of relative enrichment. Enrichment level of a positive reference gene, *FLC* was set as 1 and indicated with a horizontal dashed red line. (A~B) Each result represents the mean ± standard deviation (SD) of three independent biological replicates (*n* = 3). Different letters represent significant differences (*p* < 0.05) determined by one‐way analysis of variance (ANOVA) with Tukey's post‐hoc test. [Color figure can be viewed at wileyonlinelibrary.com]

Our epigenomic data set analyses suggested that compared to the aliphatic GSL pathway genes, indolic GSL pathway genes possess a relatively higher portion of bivalent histone marks. To investigate the presence of bivalent histone modifications, we conducted sequential ChIP analysis using anti‐H3K27me3 (repressive) and anti‐H3ac (active) mark. Total 14 selected GSL genes (9 aliphatic and 5 indolic GSL genes) were examined for bivalent state of two opposing histone marks, H3K27me3 and H3ac. The *FLOWERING LOCUS C* (*FLC*) which was used as a positive control gene because *FLC* was reported to possess these bivalent histone marks (Sung and Amasino 2004; Yu et al. 2011; He et al. 2003). As a result, we found that all tested nine aliphatic GSL genes did not show substantial enrichment of bivalent histone marks compared to that of *FLC* (Figure 8A). Meanwhile, tested five indolic GSL genes exhibited significant enrichments of bivalent histone marks on their genomic regions in comparison to that of *FLC* (Figure 8B). These results indicate that compared to aliphatic GSL genes, indolic GSL genes possess high portion of bivalent histone states on their chromatins, it may potentiate rapid induction upon wounding.

### 2.10 Indolic and Aliphatic GSL Genes Exhibit Distinct Temporal Expression Dynamics in Response to Wounding

**Figure 9:**
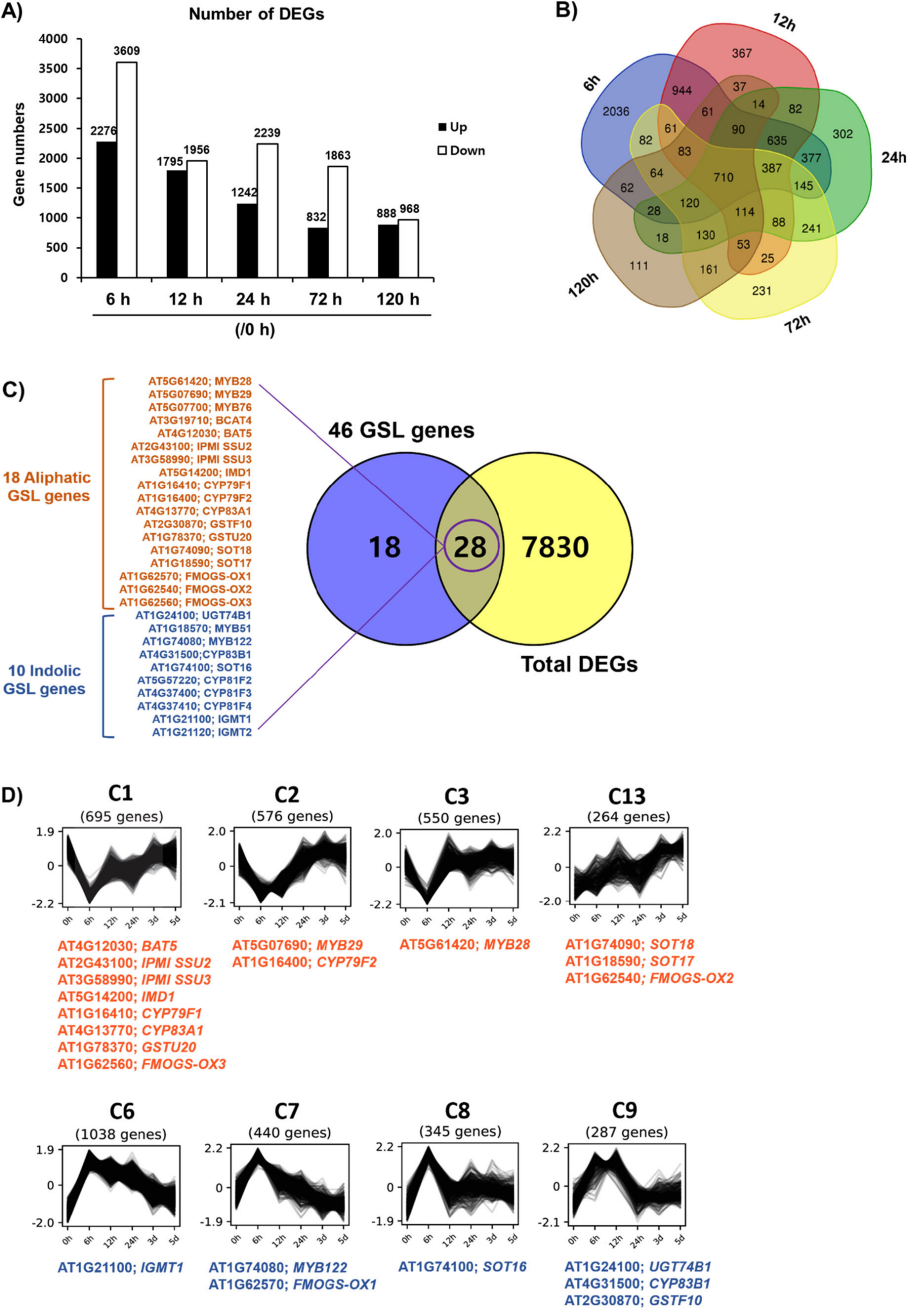
Transcriptome analysis identifying differentially expressed genes (DEGs) along the time course of wounding. (A) Bar graph showing numbers of differentially expressed genes (DEGs) in comparison of time course wounding samples (6, 12, 24, 72 and 120 h after wounding) to nontreated (0 h) sample. Number of DEGs was indicated above each bar. Among five tested time points, the most dramatic change was observed in the earliest time point, 6 h (2276 up‐ and 3609 downregulated genes). The DEGs were identified as genes with both an adjusted *p*‐value < 0.05 and an absolute log_2_ fold change ≥ 1. (B) Venn diagram showing overlapping and uniquely expressed genes between each time point sample. Total 2036, 367, 302, 231 and 111 genes were uniquely expressed at 6 h, 12 h, 24 h, 72 h and 120 h time points, respectively. (C) Venn diagram showing overlapping between 46 GSL pathway genes and total 7,858 DEGs found in the time‐course RNA‐seq analysis of this study. Total 28 GSL pathway genes including 18 aliphatic (orange colour letters) and 10 indolic GSL pathway genes (blue colour letters) were differentially expressed along wounding time course. (D) Among 20 hierarchical clustered groups (C1~C3, and C13) clusters which exhibited a slow induction upon wounding contained most of the DEGs involved in the aliphatic GSL pathway genes. Meanwhile, C6~C9 clusters which displayed a rapid induction upon wounding contained most of the indolic GSL pathway genes. DEGs involved in the aliphatic GSL biosynthesis are indicated with orange colour letters under each group, whereas DEGs involved in the indolic GSL biosynthesis are indicated with blue colour letters under each group. [Color figure can be viewed at wileyonlinelibrary.com]

Our data so far demonstrated that aliphatic and indolic GSL pathway genes are subjected to unique and bivalent histone modifications, which could lead to dynamic transcriptional changes. In line with that, we hypothesised that aliphatic and indolic GSL pathway genes could be under distinct transcriptional coordination. To test it, we performed time‐course RNA‐sequencing (RNA‐seq) analyses in Col‐0 at 6 h, 12 h, 24 h, 72 h and 120 h after wounding treatment. Multi‐dimensional scaling (MDS) plot analysis of RNA‐seq samples exhibited close clustering of the biological replicates within each sample group, indicating a high reproducibility of our RNA‐seq samples (Supporting Information Figure S11). We performed differentially expressed genes (DEG) analysis in each wounded time point sample, relative to the untreated 0 h control, revealing thousands of differentially expressed genes (DEGs) (Figure 9A–B). Among the five time points, the earliest time point, 6 h, displayed the most dramatic transcriptional change (2276 DEGs upregulated and 3609 DEGs downregulated). A larger portion (25.9%, 2,036/7,858) of DEGs at 6 h was uniquely identified compared to the other time points; 4.6% at 12 h, 3.8% at 24 h, 2.9% at 72 h, and 1.4% at 120 h. These data indicated that transcriptomic change upon wounding took place in a very rapid manner, within 6 h, and gradually relieved a later time points.

Our clustering analyses of 7,858 DEGs identified 20 clusters based on their expression patterns which exhibited distinct expression profiles (Supporting Information Figure S12 and Table S1). Among total 46 GSL pathway genes, 28 genes (61%) were found in the DEGs along the time‐course RNA‐seq (Figure 9C). Interestingly, seven indolic GSL and 14 aliphatic GSL pathway genes fell into distinctly different clusters (Figure 9D and Supporting Information Figure S12). For instance, 47% (7/15) of indolic GSL pathway genes fell into the C6, C7, C8 or C9 cluster which displayed a rapid increase early (6 h or 12 h) and then a gradual decrease at a later time point (Figure 9D). Meanwhile, 42% (14/33) of aliphatic GSL pathway genes were found at the C1, C2, C3 or C13 cluster which showed a transient reduction at 6 h and increased in a later time point (Supporting Information Figure S12). These results suggest that indolic GSL pathway genes are early inducible while aliphatic GSL pathway gene are later inducible (Figure 10A and Supporting Information Figure S13). To validate this observation, we also performed qRT‐PCR analysis on three aliphatic GSL pathway genes (e.g. *MYB28, CYP83A1* and *BCAT4*) and three indolic GSL pathway genes (*MYB34, CYP79B2* and *SOT16*) along six wounding time points. Expression patterns of these GSL genes along wound time course mimicked the result of RNA‐seq (Supporting Information Figure S14).

Figure 10Two‐phase induction between indolic and aliphatic GSLs along wounding time course (0, 6, 12, 24, 72 and 120 h). (A) A graph showing normalised transcript patterns of 28 GSL pathway genes involved in the aliphatic GSL biosynthesis (red), indolic GSL biosynthesis (green), and commonly involved genes (blue) along wounding time course. (B) Amounts of aliphatic GSLs, and indolic GSLs, and total GSLs (combining aliphatic and indolic GSLs) in a non‐wounded (Non) or wounded (Wound) samples along eight different time points after wounding treatment. Levels of aliphatic GSLs (orange colour bars), indolic GSLs (blue colour bars), and total GSLs (green colour bars) of wounded (Wound) samples at each time point were compared to those of unwounded (Non) samples (black colour bars). Variation percentage was calculated by dividing the level of GSLs of wounded samples (*n* = 3) by the level of GSLs of unwounded samples (*n* = 3) at each time point. Different letters represent significant differences (*p* < 0.05) determined by one‐way analysis of variance (ANOVA) with Tukey's post‐hoc test. (C) Amounts of five different aliphatic GSL compounds like 3‐B, 4MTB, 4OHB, 4MSOB 5MSOP along eight different time points (0, 1, 6, 12, 24, 72, 120 and 168 h) without wounding (Non, black colour bars) or with wounding (Wound, orange colour bars) treatment. (D) Amounts of four different indolic GSL compounds like I3M, 4OHI3M, 4MOI3M and 1MOI3M along eight different time points (0, 1, 6, 12, 24, 72, 120 and 168 h) without wounding (Non, black colour bars) or with wounding (Wound, green colour bars) treatment. Significance was statistically determined using one‐way analysis of variance (ANOVA) and Tukey's post‐hoc test (*p* < 0.05) and indicated with different letters above the line. (C~D) Abbreviated GSLs: 4MTB, 4‐methylthiobutyl; 4MSOB, 4‐methylsulfinylbutyl; 4OHB, 4‐hydroxybutyl; 3‐B, 3‐butenyl; 5MTP, 5‐methylthiopentyl; 5MSOP, 5‐methylsulfinylpentyl; 2PE, 2‐phenylethyl; I3M, indol‐3‐ylmethyl; 1OHI3M, 1‐hydroxyindol‐3‐ylmethyl; 1MOI3M, 1‐methoxyindol‐3‐ylmethyl; 4OHI3M, 4‐hydroxyindol‐3‐ylmethyl; 4MOI3M, 4‐methoxyindol‐3‐ylmethyl. E) A schematic model on the involvement of histone modification contexts between aliphatic and indolic GSLs pathway genes upon wounding. H3K27me3, a repressive histone mark is enriched by CLF/SWN‐containing PRC2 complex and its associated protein, LHP1 at both aliphatic and indolic GSL pathway genes. While active histone marks like H3ac are highly pre‐occupied in the indolic GSL pathway genes. Meanwhile, aliphatic GSL pathway genes were heavily accumulated with H3K27me3 and less occupied with active histone marks. Upon wounding, bivalent indolic GSL pathway genes (containing both H3ac and H3K27me3) results in the rapid induction of indolic GSL pathway genes upon wounding. In contrast, H3ac‐depleted aliphatic GSL pathway genes exhibit slow kinetics of gene induction related to the aliphatic GSL biosynthesis upon wounding in Arabidopsis. These distinct histone contexts lead to a dual mode of GSLs induction in *Arabidopsis*, with early induction of indolic GSLs and delayed induction of aliphatic GSLs. [Color figure can be viewed at wileyonlinelibrary.com]

**Figure:**
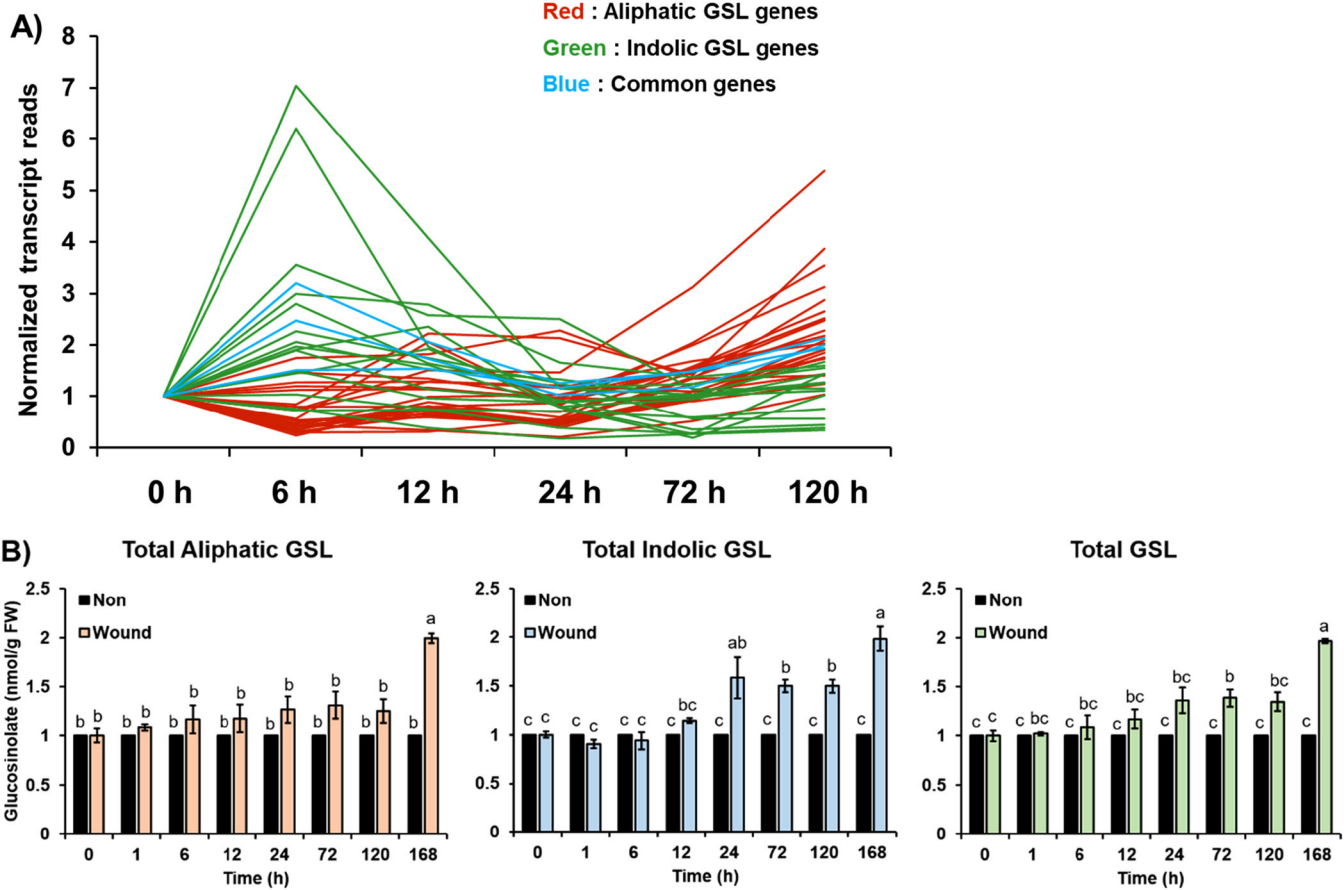

**Figure:**
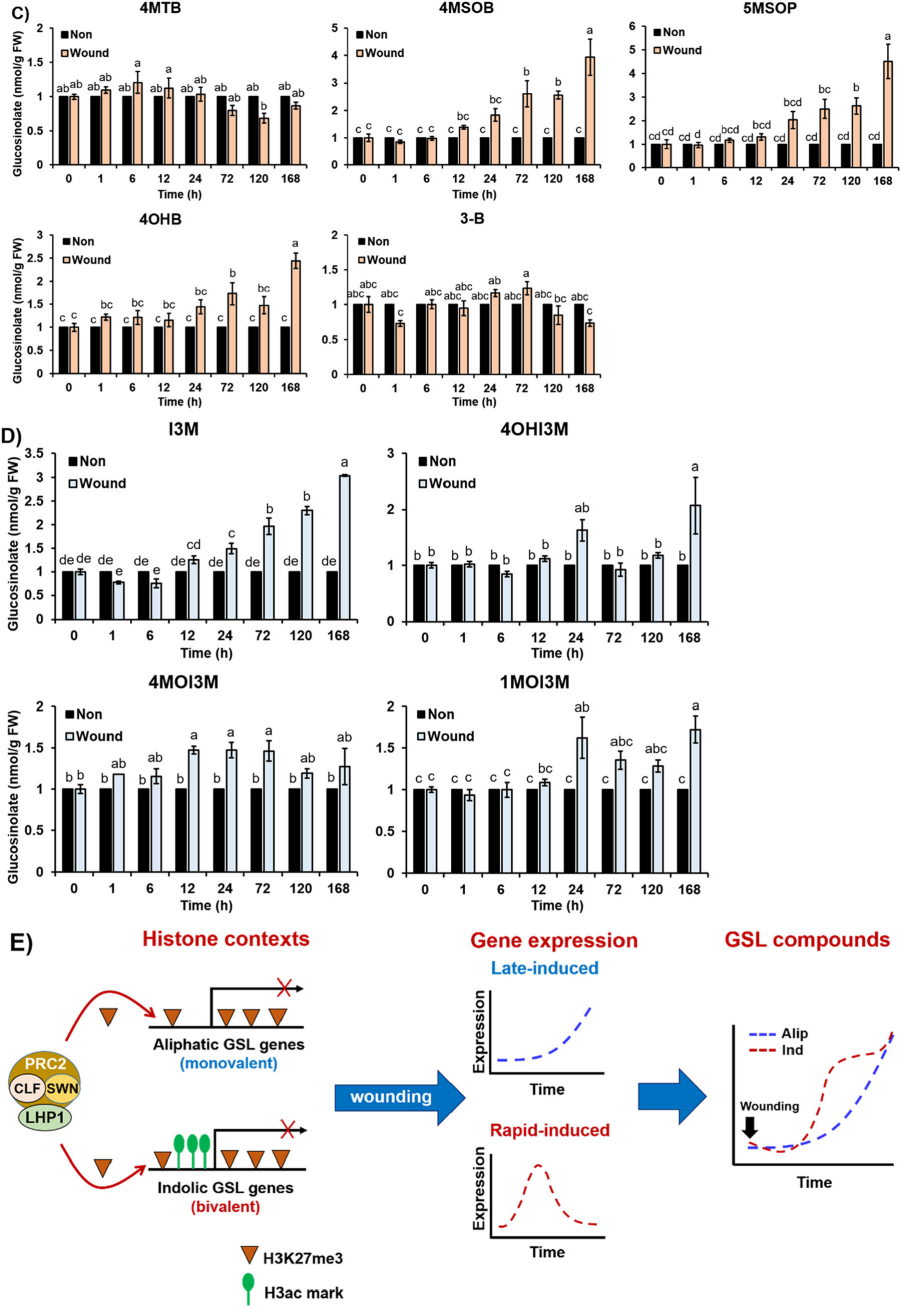

Next, we wondered the different expression patterns between aliphatic and indolic GSL pathway genes resulted in different GSL production between aliphatic and indolic GSLs. To examine this, we quantified levels of GSLs along eight different (0, 1, 6, 12, 24, 72, 120 and 168 h) wounding time points between non‐wounded time point samples and treated time point samples. Because metabolite accumulation generally occurs later than transcriptional changes due to factors such as enzyme stability, metabolic flux, and storage or degradation processes (Gibon et al. 2006; Howell et al. 2009), we included an additional 168‐h time point in the metabolite profiling. As a result, compared to the non‐wounded time point samples, wounded samples exhibited increased production of both total aliphatic and indolic GSLs (Figure 10B). In particular, levels of indolic GSL compounds were earlier induced (from 12 h after wounding) than those of aliphatic GSLs (168 h after wounding), exhibiting similar pattern shown in the time point RNA‐seq data set. Among five aliphatic GSL compounds, three aliphatic GSLs like 4MSOB, 5MSOP, and 4OHB exhibited the delayed induction patterns, whereas other two aliphatic GSL compounds like 4MTB and 3‐B did not show any significant changes between non and wound samples along wounding time points (Figure 10C). In the case of indolic GSL compounds, the four individual indolic GSLs (I3M, 4OHI3M, 4MOI3M, and 1MOI3M) showed slightly different induction patterns but were commonly induced early, from around 6 to 24 h after wounding, compared to the aliphatic GSL compounds (Figure 10D).

Based on these findings, we propose a schematic model illustrating the dynamic transcriptional behaviour of indolic and aliphatic GSL biosynthetic pathways in response to wounding (Figure 10E). The rapid induction of indolic GSL pathway genes suggests that their bivalent chromatin configuration, marked by both H3K27me3 and H3ac may facilitate swift transcriptional activation and early production of indolic GSLs. In contrast, many aliphatic GSL pathway genes lack active histone marks such as H3ac. The lower enrichment of H3ac likely delays the transition from a repressive chromatin state (enriched with H3K27me3) to an active state, resulting in slower and more gradual activation of aliphatic GSL genes and delayed accumulation of aliphatic GSLs. Together, our results demonstrate that the distinct histone modification landscapes between aliphatic and indolic GSL genes contribute to their differential transcriptional timing, ultimately producing temporally separated peaks of GSL accumulation in *Arabidopsis* upon wounding.

## 3 Discussion

A previous study demonstrated that overexpression of *MYB34* upregulates ‘core structure formation’ genes such as *CYP79B2, CYP79B3, CYP83B1, SUR1* and *SOT16* in the indolic GSL pathway (Celenza et al. 2005). Thus, we expected similar gene activation in *lhp1‐4*, where *MYB34* was upregulated (Supporting Information Figure S1A). While *CYP79B3* and *SUR1* were indeed upregulated, *CYP79B2, CYP83B1, GGP1* and *SOT16* were either unaffected or downregulated (Supporting Information Figure S1B–S1C). This expression pattern mirrored that of *MYB51* in *lhp1‐4* (Supporting Information Figure S1A). Since *MYB51* is the most dominantly expressed *MYB* gene in the indolic GSL pathway, we postulated that downregulation of *MYB51* may override the effect of *MYB34* upregulation in *lhp1‐4* mutant, resulting in reduced expression of downstream GSL biosynthetic genes like *CYP79B2, CYP83B1, GGP1* and *SOT16*. Functional diversity between MYB34 and MYB51 needs further investigation.

In Figure 3, it was shown that overexpression of *LHP1* resulted in stronger repression of several targets (*MYB29, MYB76, CYP79F1, FMOGS* and *AOP3*) than in the Col‐0 wild type. Several, not mutually exclusive, explanations might be possible on this observation. First, because the *LHP1* transgene is driven by the constitutive CaMV35S promoter, LHP1 is ectopically and broadly overexpressed, likely extending its repressive activity to tissues or developmental stages in which LHP1 is normally limited. Consequently, these targets may be suppressed more broadly and more strongly than under normal wild‐type condition. In other words, constitutive overexpression from CaMV35S promoter can suppress target expression even in cell types, cell‐cycle phases, or developmental stages where endogenous LHP1 levels are typically low, thus resulting in the hyper‐repression of those genes. Second, prior studies indicate that LHP1 interacts not only with PRC2 components but also with the components of PRC1 complex, which deposits H2Aub1 on chromatin (Xu and Shen 2008; Chen et al. 2010). Overexpressed LHP1 might therefore drive excessive PRC1‐dependent H2Aub1 deposition and/or chromatin remodelling, further reinforcing repression of these target genes. Further assays such as ChIP–seq for H2Aub1 and ATAC‐seq might provide more understanding on this observation.

MYC2, a key transcription factor in jasmonic acid (JA) signalling, also regulates GSL pathway genes. We investigated whether *MYC2* expression was affected in PRC2 mutants and could explain altered expression of GSL pathway genes (Supporting Information Figure S15). However, transcript levels of *MYC2* was unchanged in both *lhp1‐4* mutant and *35S::GFP‐LHP1/lhp1‐4* line compared to those of Col‐0, suggesting that MYC2 and JA signalling are not under direct control of PRC2 in *Arabidopsis*.

Compared to the high proportion of GSL genes marked by H3K27me3 (63%, 29 of 46 genes), fewer were marked by activating modifications: H3K4me3 (39%, 18 of 46 genes) and H3ac (46%, 21 of 46 genes) (Figures 6, 7, and Supporting Information Figure S9). This suggests that under non‐stressed conditions, GSL metabolism is largely suppressed at the transcriptional level. However, it is still not revealed which epigenetic activator(s) is involved in the transcriptional activation upon wounding. It might be an important area of future research. In addition, it remains unclear how these aliphatic and indolic GSL pathway genes are transcriptionally modulated in response to other environmental stresses like salinity, drought, and insect attack and so on.

By integrating transcriptome, epigenome, metabolome, and transgenic approaches, we demonstrate that GSL pathway genes are suppressed by the repressive histone mark, H3K27me3 under non‐stressed conditions. Upon wounding stress, indolic GSL pathway genes, which are pre‐marked by active chromatin states (e.g., H3K4me3 and H3ac), are rapidly induced, followed by a delayed induction of aliphatic GSL genes. We propose that this two‐phase induction reinforces the plant defence system, offering a strategic advantage against wounding and herbivory. As climate change increases the prevalence of biotic stress, our findings offer valuable epigenetic insights into GSL pathway regulation and provide a molecular foundation for enhancing crop resilience through breeding strategies.

## 4 Experimental Procedures

### 4.1 Plant Materials and Growth Conditions

Wild type (Col‐0) and the mutant seeds were sterilised and placed on the 0.5x Murashige and Skoog (MS) agar medium. After cold‐stratification of sterilised seeds at 4°C for 2−3 days in the dark condition, seeds were transferred to 22°C growth chamber and grown under white fluorescent light (100 μmol/m^2^/s) in the long day (16 h light: 8 h dark) condition for 2 weeks before further experiments including HPLC, RNA‐seq and qRT‐PCR analyses. In this study, all of the mutants and transgenic plants were derived from the Col‐0 ecotype background. The *lhp1‐4* (referred also as *tfl2‐2)* mutant was previously described (Larsson et al. 1998). The *35S::LHP1‐GFP/lhp1‐4* transgenic plants were previously described (Kotake et al. 2003). All mutants and transgenic plants analysed in this study were generated in the Col‐0 ecotype background, ensuring that no other wild‐type accessions (e.g., Ler‐0 or Ws‐2) were used.

### 4.2 Extraction and Analysis of GSLs

Arabidopsis seedlings grown for 2 weeks in the long day condition (16 h light/8 h dark) were used for GSLs extraction. GSLs were extracted as desulfo‐GSLs (DS‐GSLs) as described previously (Han et al. 2019). In short, about 100 mg of fresh samples were ground in liquid nitrogen and incubated with 70% MeOH at 70°C immediately for 25 min to inactivate the myrosinase enzyme activity. Subsequently, the methanol extract was reacted sephadex with 11.25 units of sulfatase (Sigma‐Aldrich, USA) for 14 h at 37°C in a polypropylene column (Thermo Scientific, USA); 0.5 mg/ml of sinigrin (Sigma‐Aldrich, USA) was used as an internal standard. Each DS‐GSL was analysed with ultrahigh‐performance liquid chromatography (3000 UHPLC System, Thermo Scientific, USA). The DS‐GSL was separated on a C18 reverse phase column (Zorbax XDB‐C18, 4.6 × 250 mm, 5 μm particle size, Agilent, USA) with a water and acetonitrile gradient system. Samples with 20 μL volume were injected and maintained at 1.0 mL min^‐1^ flow rate. Individual peaks were identified using standard compounds (Phytoplan, Germany), and sinigrin was used for relative quantification (Brown et al. 2003). The samples were analysed independently with three replicates and presented in nmol g^‐1^ on a fresh weight (FW) basis.

### 4.3 Analysis of GSLs by LC‐DAD‐ESI‐MS (Liquid Chromatography–Diode Array Detection–Electrospray Ionisation–Mass Spectrometry)

About 400 mg of fresh samples were ground in liquid nitrogen and used for LC‐DAD‐ESI‐MS analysis. Contents of DS‐GSLs were analysed using the Accela ultrahigh‐performance liquid chromatography system (Thermo Scientific, USA) combined with an ion trap mass spectrometer (LTQ Velos Pro, Thermo Scientific, USA). The samples (25 μL) were separated in C18 reverse phase column (Zorbax XDB‐C18, 4.6 × 250 mm, 5μm particle size, Agilent, USA) with water and acetonitrile mobile phase, and determined in negative ion mode ([M‐H]‐). Mass‐spectrometry (MS) operating conditions were conducted as shown follows: capillary temperature (275°C), capillary voltage (5 kV), source heater temperature (250°C), sneath gas flow (35 arb), auxiliary gas flow (5arb), and spectra scanning range (m.z 100 ~ 1500). Particularly, the molecular mass ions of DS‐GATN was identified based on the previous report (DS‐GATN m/z 310, 356, 621) (Petersen et al. 2002; Brown et al. 2003; Kusznierewicz et al. 2013).

### 4.4 Time Course Wounding Treatment

For both RNA‐seq and HPLC‐based metabolite analyses using wounded samples, we cut twice the tips of the cotyledons of 2‐week‐old seedlings and then collected the entire seedlings along different wounding time points. For transcriptome analysis, seedlings were harvested at 0 h (untreated control), and at 6, 12, 24, 72 and 120 h after wounding. Approximately 100 mg of each sample was used for RNA extraction. For time course GSL measurement of non‐wounded and wounded samples, approximately 200 mg of seedling samples were collected at 0, 1, 6, 12, 24, 72, 120 and 168 h after wounding treatment.

### 4.5 RNA Extraction and qRT‐PCR Analysis

Arabidopsis seedlings grown for 2 weeks in the long day condition (16 h light/8 h dark) were collected and immediately frozen in liquid nitrogen for RNA extraction. Total RNAs were extracted using the RNeasy Plant Mini Kit (QIAGEN, Valencia, CA). To eliminate genomic DNA contamination, extracted total RNAs were treated with DNase 1 (New England Biolabs, USA). Around 2μg of total RNAs were used for cDNA synthesis using EasyScript reverse transcriptase (TransGen Biotech, China). qRT‐PCR analysis was performed using BioFACT 2x Real‐Time PCR Master mix (BioFACT, Republic of Korea) on a LineGene 9600 Plus Real‐Time PCR system (BioER, China) according to the manufacturer's instructions. Information on primers used in the qRT‐PCR analysis is listed in Supporting Information Table S2.

### 4.6 RNA‐Seq Library Preparation and Sequencing

For each time point sample, three biological replicates were collected and directly frozen in liquid nitrogen before total RNA extraction. Total RNAs were isolated using the RNeasy Plant Mini Kit (QIAGEN, Valencia, CA) according to the manufacturer's instructions. The quantity and quality of RNAs were checked with a Nano‐400 (Allsheng Instrument Co., China). Preparation of RNA‐seq libraries was conducted using the TruSeq Stranded mRNA LT Sample Prep Kit according the manufacturer's instruction (Illumina Inc., USA). Paired‐end sequencing of the RNA‐seq libraries was performed using an Illumina NovaSeq. 6000 platform (Macrogen Co., Republic of Korea).

### 4.7 RNA‐Seq Data Analyses

The FastQC programme (www.bioinformatics.babraham. ac. uk/projects/fastqc/) was used to evaluate the quality of FASTQ raw reads. Low qualities of raw reads were trimmed out using Trimmomatic programme before alignment to the reference genome (Bolger et al. 2014). Trimmed reads of each sample were aligned to the Arabidopsis TAIR10 genome using STAR aligner with default parameters (Dobin et al. 2013). Following alignment, downstream analyses were performed in R (ver. 3.6.1) packages including featureCounts and edgeR. Briefly, aligned reads were quantified with featureCounts (Liao et al. 2014) and differentially expressed genes (DEGs) were identified with edgeR (Robinson et al. 2009). A GitHub repository containing a script used for RNA‐seq analysis is available at https://github.com/kdhjeje1/RNA-seq-analysis-using-STAR-aligner. Venn diagram analysis was carried out using VENNY (https://bioinfogp.cnb.csic.es/tools/venny/), while Gene Ontology (GO) enrichment analysis was performed with ShinyGO (ver. 0.77; http://bioinformatics.sdstate.edu/go/). Hierarchical clustering was conducted using a Python‐based in‐house script. Mapping results were visualised with the Integrative Genomics Viewer (IGV) (Thorvaldsdottir et al. 2013).

### 4.8 ChIP‐Seq Data Analysis

Publicly available ChIP‐seq data were downloaded from the EBI‐EMBL website. The raw FASTQ files were trimmed and quality‐filtered. Filtered reads were aligned to the *Arabidopsis thaliana* TAIR10 reference genome by using Bowtie2 (Langmead and Salzberg 2012). Peak callings were performed by using MACS2 (Zhang et al. 2008). Aligned reads were converted into bigwig files for visualisation using the IGV genome browser (Robinson et al. 2011). A GitHub repository containing a script used for ChIP‐seq analysis is available at https://github.com/kdhjeje1/ChIP-seq-analysis-for-mapping-and-bigwig-file-generation-of-epigenetic-mark‐. Detailed information on ChIP‐seq data used in this study is described in Supporting Information Table S3.

### 4.9 ChIP‐qPCR Analysis

Two‐week‐old seedlings were cross‐linked with 1% formaldehyde solution under vacuum for 25 min, then terminated by addition of 0.125 M glycine. Cross‐linked seedlings were dried and then frozen in liquid nitrogen. ChIP‐qPCR analysis using H3K27me3 between Col‐0 and *lhp1‐4* mutant was performed as previously described (Kim and Sung 2017). For sequential ChIP assay was conducted with 10 micrograms of monoclonal antibody against H3K27me3 histone mark (ab6002, Abcam, United Kingdom) and anti‐H3ac histone mark (06‐599, Merck, Germany) were used. Aliquots of the immuno‐precipitated and eluted input DNAs were used for qPCR analysis. Quantitative PCR analysis was performed using BioFACT 2x Real‐Time PCR Master mix (BioFACT, Republic of Korea) in a LineGene 9600 Plus Real‐Time PCR system (BioER, China). Individual PCR reaction was conducted as follows: total 45 cycles of denaturation at 95°C for 15 s, annealing at 60°C for 25 s, and extension at 72°C for 35 s. Information on the primers used in the ChIP‐qPCR analysis was shown in the Supporting Information Table S2.

### 4.10 Statistical Analysis

All statistical analyses in this study were conducted using SAS software (version 9.4; SAS Institute Inc., Cary, NC, USA). Statistical differences were evaluated by one‐way analysis of variance (ANOVA), and values of *p* < 0.05 were considered significant. Significant differences were denoted by different alphabetical letters. Data are presented as means ± standard deviation (SD) of three biological replicates.

## Conflicts of Interest

The authors declare no conflicts of interest.

## Supporting information

(fv) SUPPORTING INFORMATION dd.

Supp Table S1.

## Data Availability

The data that support the findings of this study are openly available in NCBI Gene Expression Omnibus at https://www.ncbi.nlm.nih.gov/geo/query/acc.cgi?acc=GSE239725, reference number GSE239725. RNA‐seq raw files generated in this study were deposited in the NCBI Gene Expression Omnibus with GEO accession number (GSE239725). The custom scripts employed for RNA‐seq and ChIP‐seq analyses in this study are available in public GitHub repositories: RNA‐seq analysis scripts at https://github.com/kdhjeje1/RNA-seq-analysis-using-STAR-aligner and ChIP‐seq analysis scripts at https://github.com/kdhjeje1/ChIP-seq-analysis-for-mapping-and-bigwig-file-generation-of-epigenetic-mark‐.
