# Supplementary material for: Bivalent Histone Modifications Orchestrate Temporal Regulation of Glucosinolate Biosynthesis During Wound‐Induced Stress Responses in Arabidopsis
Source: Plant Cell Environ. 2025 Oct 8;49(1):450–73. doi: 10.1111/pce.70232 (PMC12675981; doi:10.1111/pce.70232)
Supplement: Supplementary file 1 — (fv) SUPPORTING INFORMATION dd. [file PCE-49-450-s001.docx]

**SUPPORTING INFORMATION**

**Supplementary Fig. S1**

**
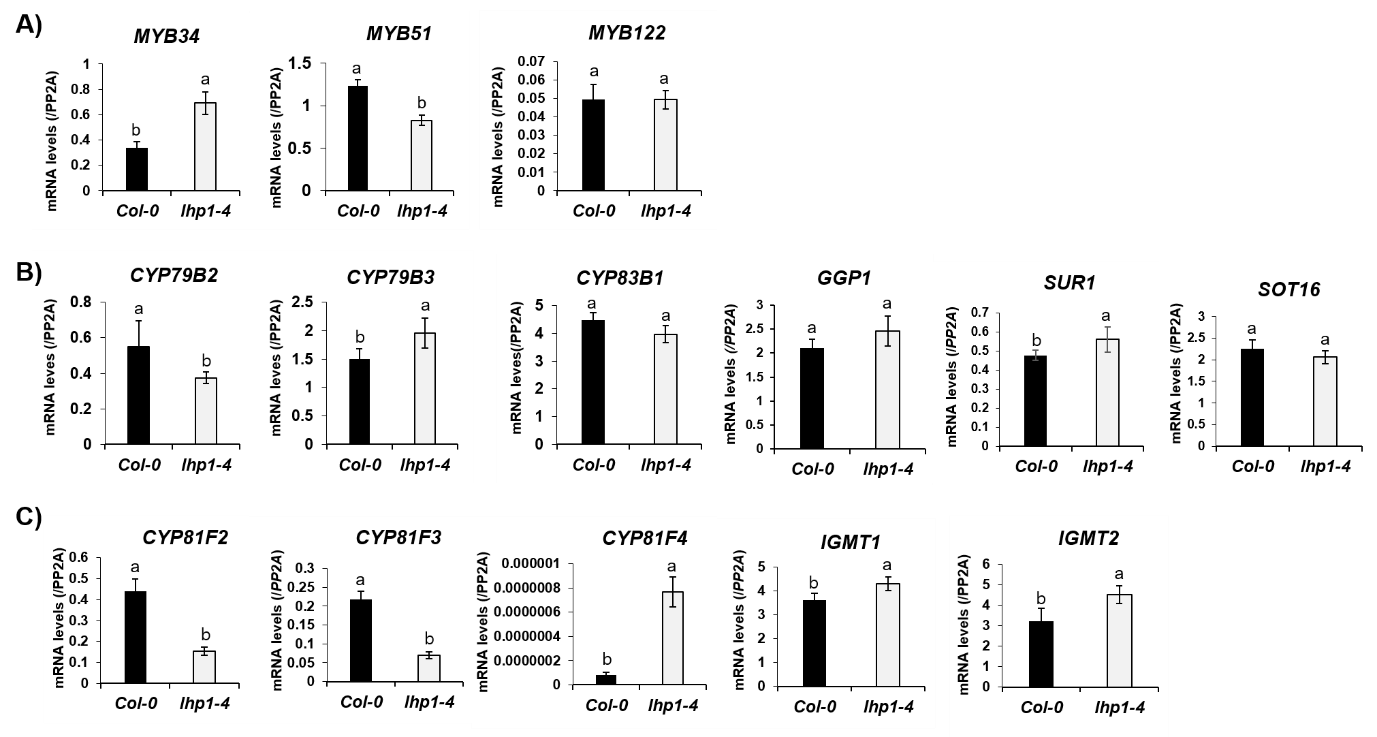
**

**Supplementary Fig. S1. Comparison of transcript levels of genes related to indolic GSL pathway genes between Col-0 and the *lhp1-4* mutant. A)** Result of qRT-PCR on four TF (*MYB34, MYB51*, and *MYB122*) genes between Col-0 and the *lhp1-4* mutant. **B)** Result of qRT-PCR on six ‘core structure formation’ phase genes (*CYP79B2, CYP79B3, CYP83B1, GGP1, SUR1*, and *SOT16*) between Col-0 and the *lhp1-4* mutant. **C)** Result of qRT-PCR on five ‘secondary modification’ phase genes (*CYP81F2, CYP81F3, CYP81F4, IGMT1,* and *IGMT2*) between Col-0 and the *lhp1-4* mutant. Significance was statistically determined using one-way analysis of variance (ANOVA) and Tukey’s post-hoc test (*p* < 0.05) and indicated with different letters above bars.

**Supplementary Fig. S2**

**
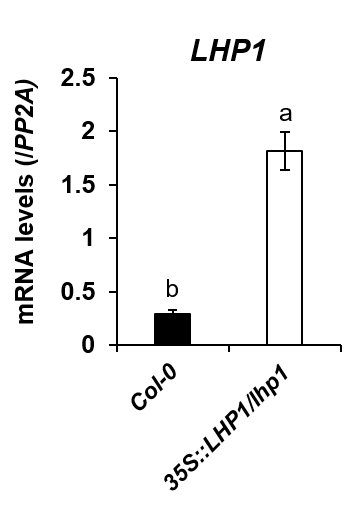
**

**Supplementary Figure S2.** Transcript levels of *LHP1* between Col-0 and *35S::LHP1/lhp1-4* transgenic plants. Significance was statistically determined using one-way analysis of variance (ANOVA) and Tukey’s post-hoc test (*p* < 0.05) and indicated with different letters above the line.

**Supplementary Fig. S3**

**
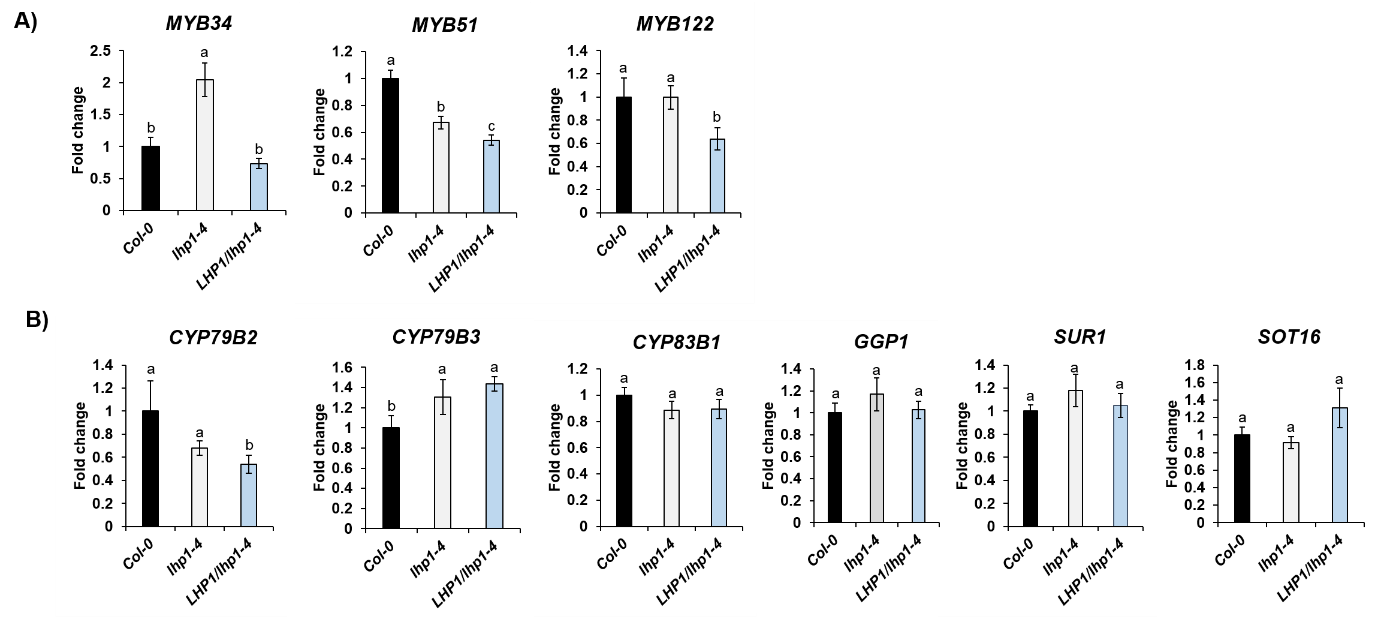
**

**
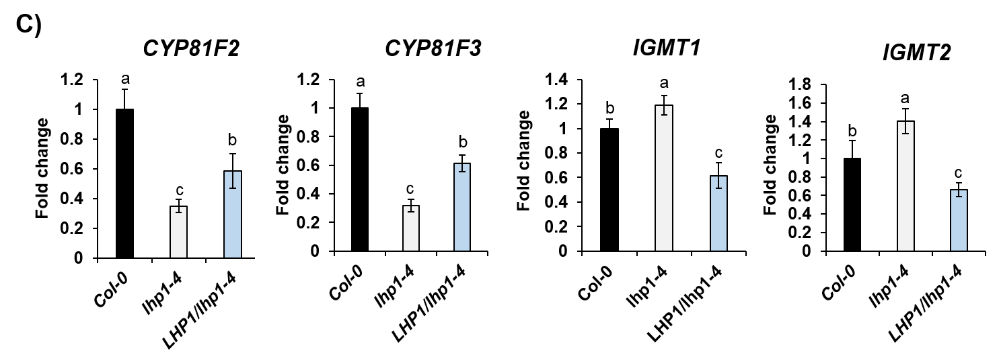
**

**Supplementary Fig. S3. Comparison of transcript levels of genes related to indolic GSL pathway genes between Col-0, *lhp1-4* mutant, and *35S::LHP1/lhp1-4* transgenic line. A)** Relative transcript levels of three TF (*MYB34, MYB51*, and *MYB122*) genes between Col-0, *lhp1-4* mutant, and *35S::LHP1-GFP/lhp1-4* transgenic line. **B)** Relative transcript levels of six ‘core structure formation’ phase genes (*CYP79B2, CYP79B3, CYP83B1, GGP1, SUR1,* and *SOT16*) between Col-0, *lhp1-4* mutant, and *35S::LHP1-GFP/lhp1-4* transgenic line. **C)** Relative transcript levels of on four ‘secondary modification’ phase genes (*FMO GS-OX1*, *FMO GS-OX3*, and *AOP3*) between Col-0, *lhp1-4* mutant, and *35S::LHP1-GFP/lhp1-4* transgenic line. **A)~C)** Significance was statistically determined using one-way analysis of variance (ANOVA) and Tukey’s post-hoc test (*p* < 0.05) and indicated with different letters above bars.

**Supplementary Fig. S4.**


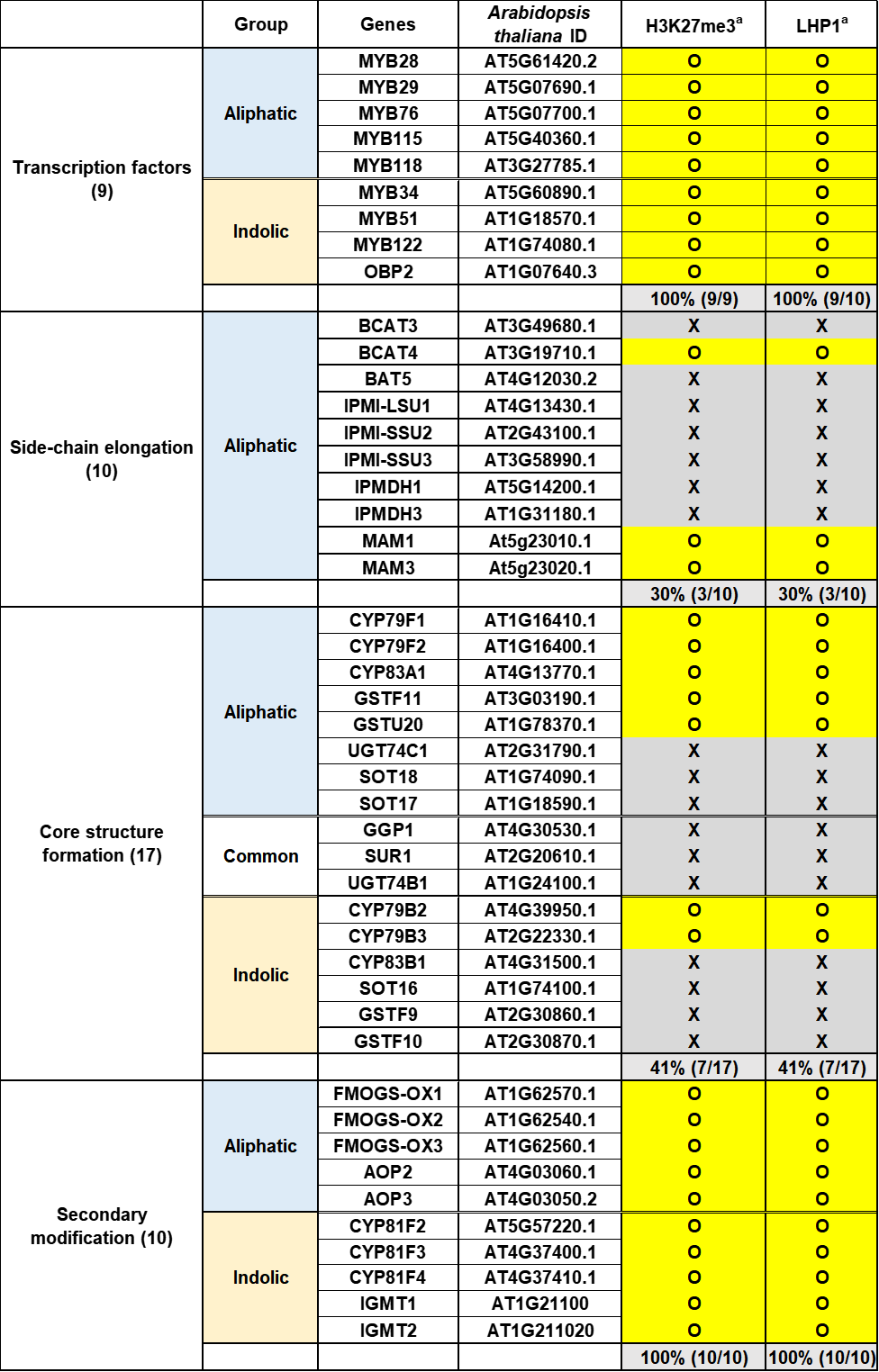


**Supplementary Figure S4.** A table showing the enrichment of H3K27me3 and LHP1 on 9 TFs, 10 ‘side-chain elongation’ phase genes, 17 ‘core structure formation’ phase genes, and 10 ‘secondary modification’ phase genes involved in the aliphatic and indolic GSL biosynthesis of Arabidopsis. Enrichment of H3K27me3 and LHP1 is indicated with “O” symbol and yellow box. Meanwhile, no enrichment of H3K27me3 and LHP1 is indicated with “-” symbol and gray box. Informations on the ChIP-seq results analyzing enriched levels of H3K27me3, LHP1, and H3K9me2 were described in the Supplementary Table S3.

**Supplementary Fig. S5**

**
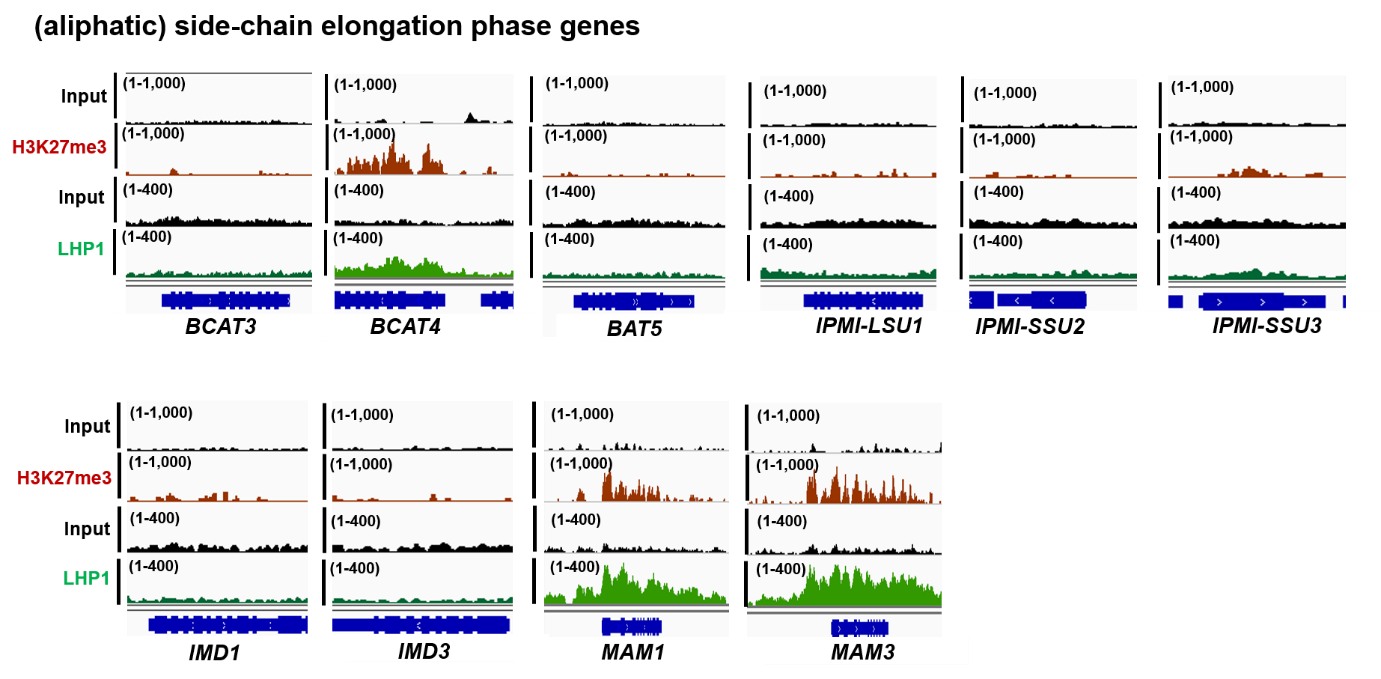
**

**Supplementary Fig. S5. Genome browser view of H3K27me3 and LHP1 enrichment profile on ‘side-chain elongation’ phase genes in aliphatic GSL pathway.** Aligned ChIP-seq reads of H3K27me3 and LHP1 on ten ‘side-chain elongation’ phase genes involved in the aliphatic GSL pathway were presented with red and green colors, respectively. Aligned ChIP-seq reads of Input DNA were presented with black color. Only three genes (*BCAT4, MAM1*, and *MAM3*) were enriched with H3K27me3 and LHP1, whereas seven genes like *BCAT3, BAT5, IPMI-LSU1, IPMI-SSU2, IPMI-SSU3, IMD1, and IMD3*) were not enriched with both H3K27me3 and LHP1 in comparison to the level of input DNA. It indicates that ‘side-chain elongation’ phase genes are not tightly controlled by PRC2 suppressive complex. Read coverage normalized using a total number of mapped reads is indicated at the top right corner of each track in parenthesis.

**Supplementary Fig. S6**

**
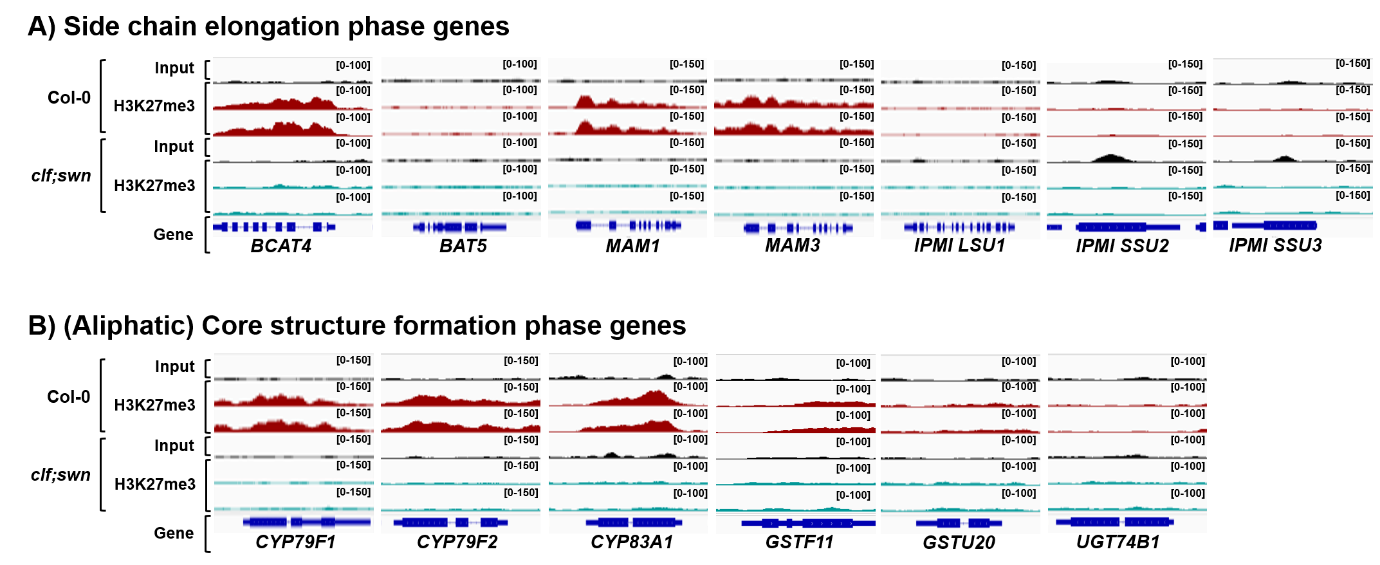
**


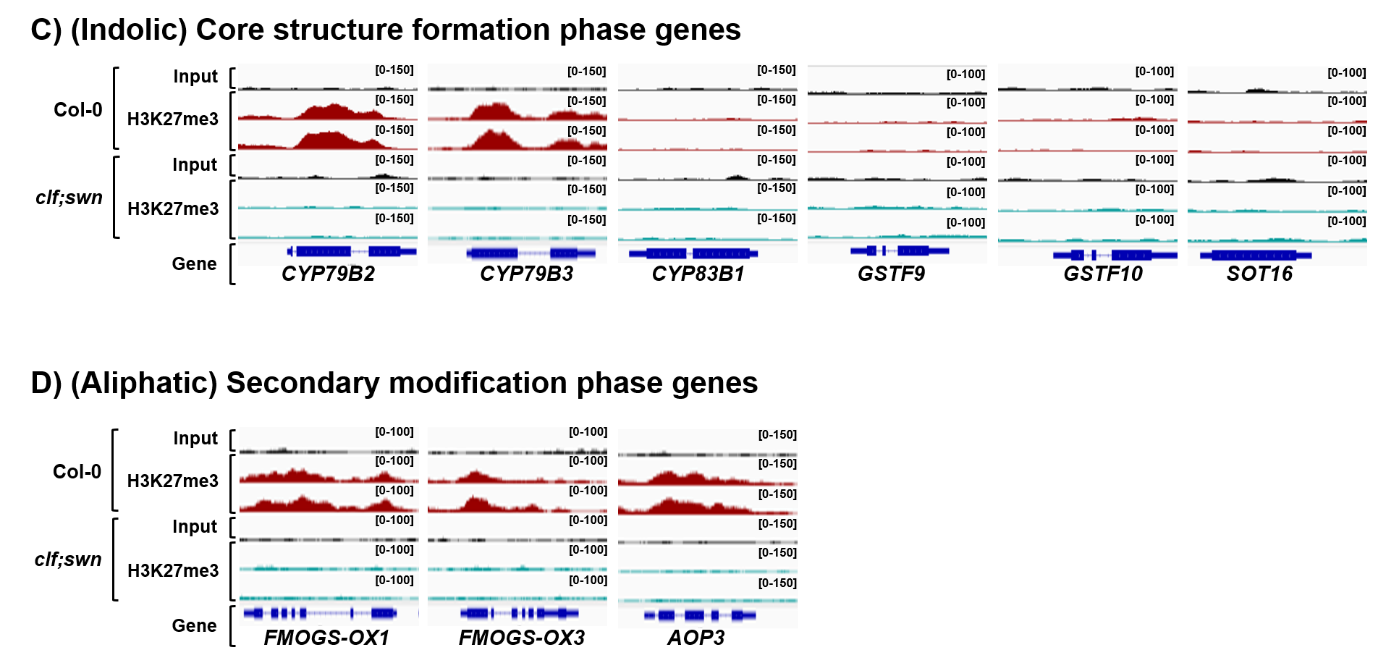


**
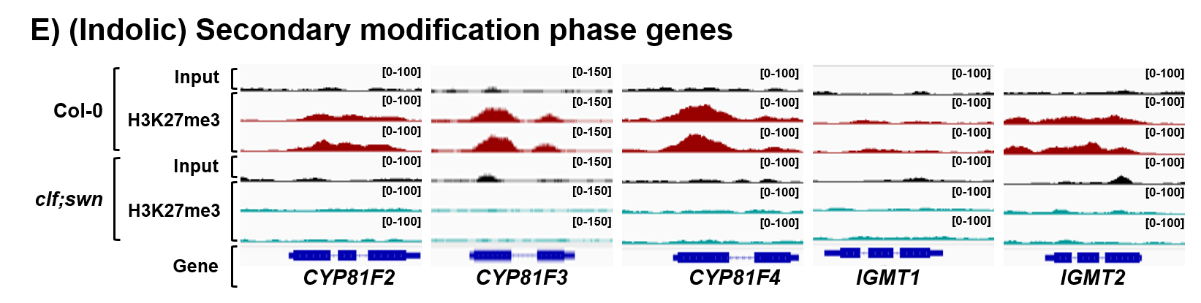
**

**Supplementary Fig. S6. Comparison of enriched levels of H3K27me3 on genes involved in the aliphatic and indolic GSL biosynthesis between Col-0 and the *clf-29;swn-4 (clf;swn)* double mutant. A)** The enriched levels of H3K27me3 on seven ‘side-chain elongation’ phase genes in the aliphatic GSL pathway between Col-0 and the *clf;swn* mutant. **B)** The enriched levels of H3K27me3 on six ‘core structure formation’ phase genes in the aliphatic GSL pathway Col-0 and the *clf;swn* background. **C)** The enriched levels of H3K27me3 on six ‘core structure formation’ phase genes in the indolic GSL pathway between Col-0 and the *clf;swn* mutant. **D)** The enriched levels of H3K27me3 on three ‘secondary modification’ phase genes in the aliphatic GSL pathway between Col-0 and the *clf;swn* mutant. **E)** The enriched levels of H3K27me3 on five ‘secondary modification’ phase genes in the indolic GSL pathway between Col-0 and the *clf;swn* mutant. **A)~E)** Read coverage normalized using a total number of mapped reads are indicated at the top right corner of each track in the bracket. Information on the public available genome-wide H3K27me3 dataset between Col-0 and the *clf;swn* mutant were shown in the Supplementary Table S3.

**Supplementary Fig. S7**

**
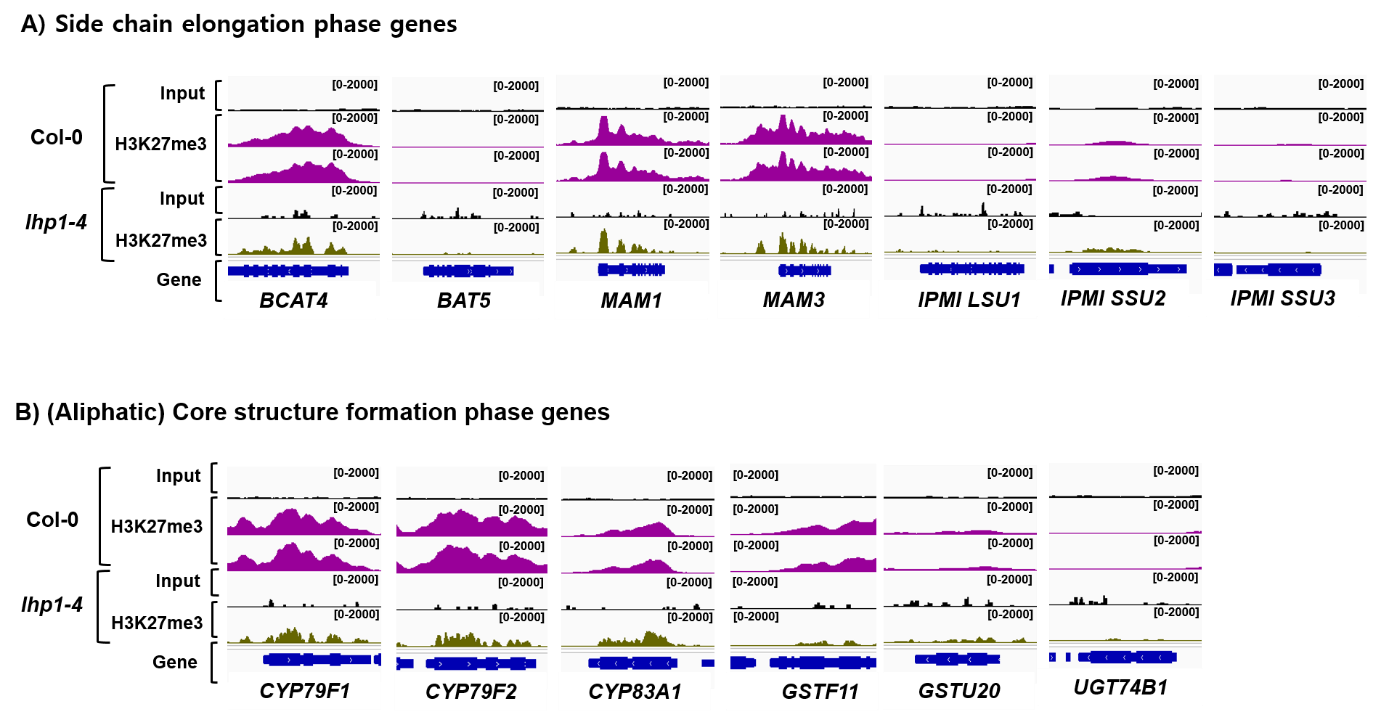
**

**
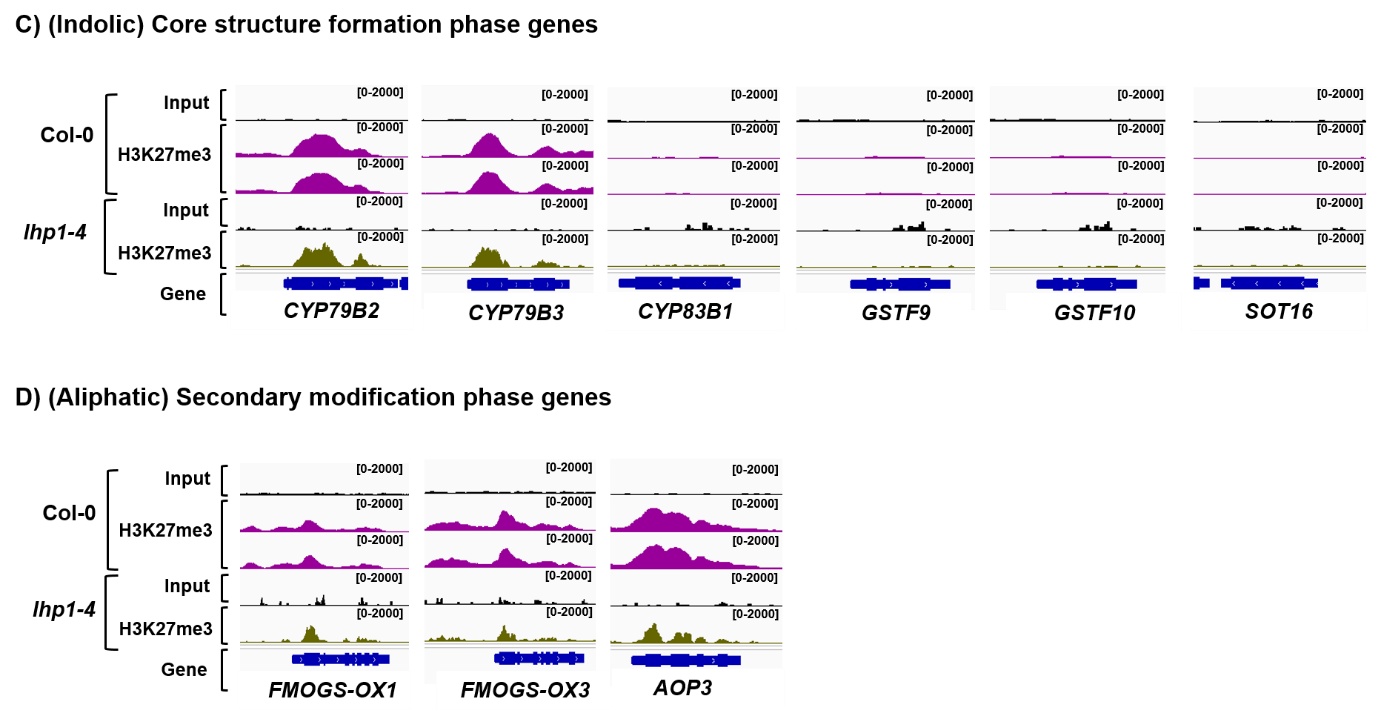
**

**
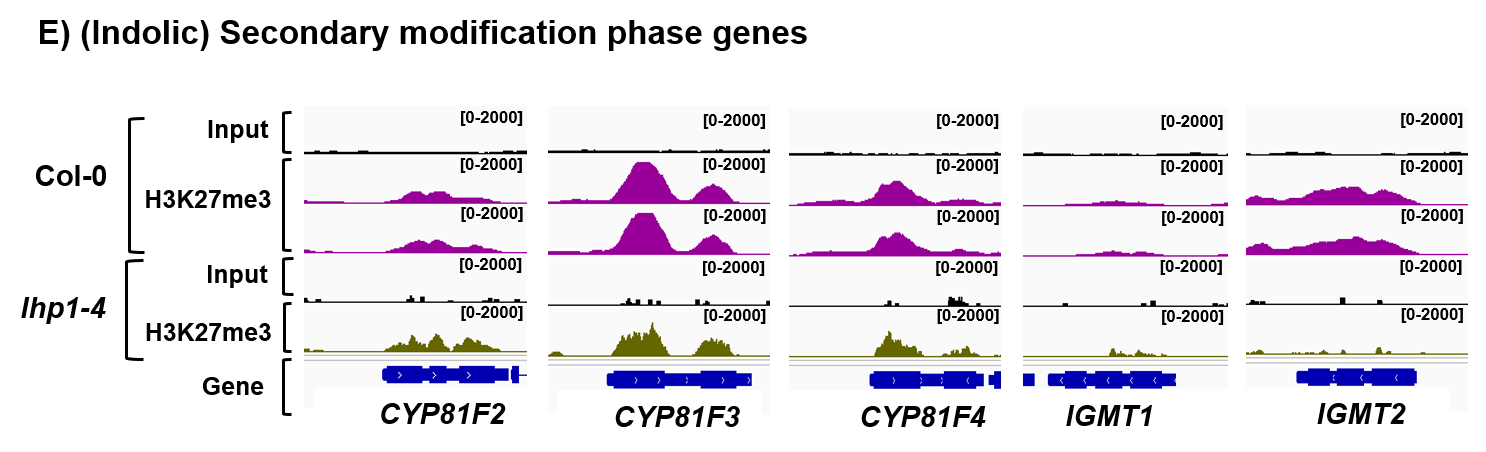
**

**Supplementary Fig. S7.** Illustration of genomic IGV browser showing normalized H3K27me3 enrichments of aliphatic GSL pathway genes (**A, B**, and **D**) and indolic GSl pathway genes (**C** and **E**) between Col-0 and *lhp1-4*, a knock-out mutant of LHP1 were presented in. A repressive histone mark, H3K27me3 were enriched at many aliphatic and indolic GSL pathway genes in Col-0 wild type (purple color). Meanwhile, a *lhp1-4* mutant showed severe reductions of H3K27me3 (dark brown color) compared to levels of Col-0. Tracks for input DNA were indicated with black color. Read coverage normalized using the total number of mapped reads are indicated at the top right corner of each track in the bracket. Information on the public ChIP-seq dataset on enriched levels of H3K27me3 between Col-0 and *lhp1-4,* a knock-out mutant of LHP1 was described in the Supplementary Table S3.

**Supplementary Fig. S8**

**
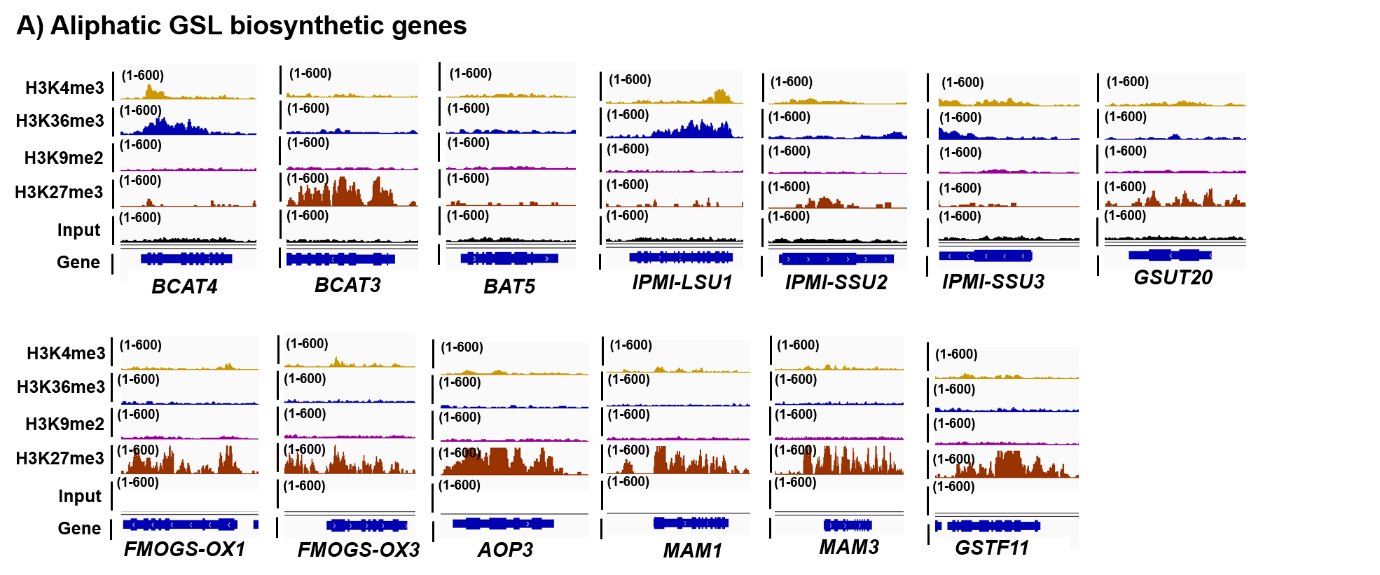
**

**
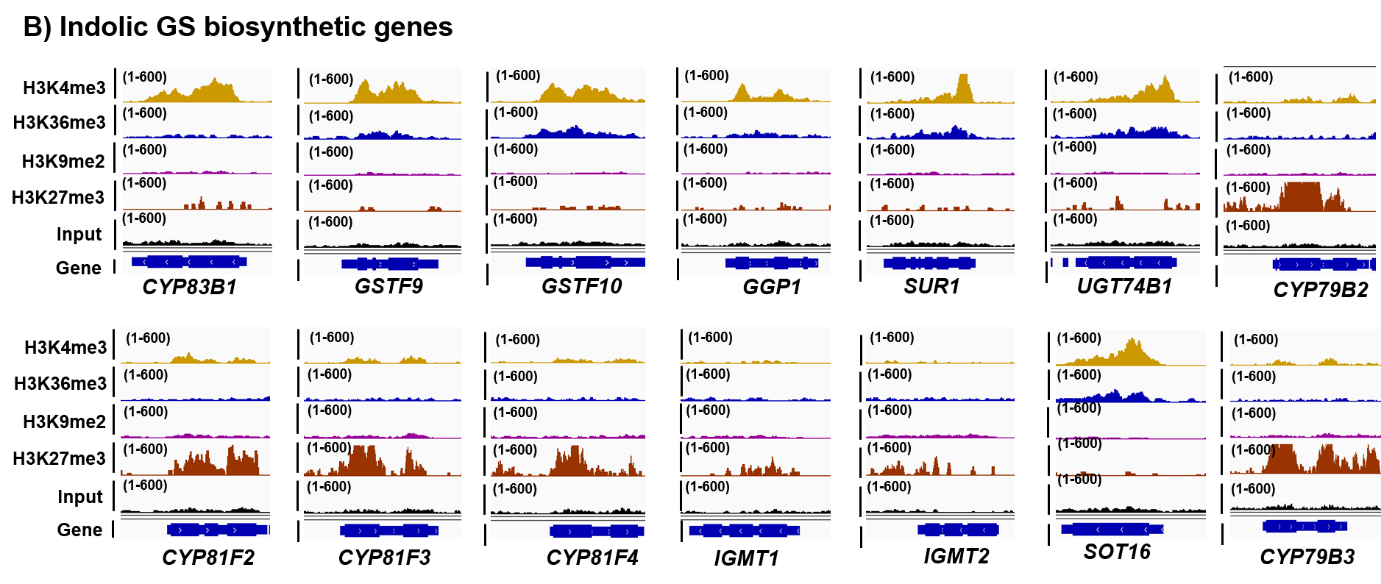
**

**Supplementary Fig. S8. Histone methylation mark profiles on TFs, ‘side-chain elongation’, ‘core structure formation’, and ‘secondary modification’ phase genes involved in the aliphatic (A) and indolic GSL (B) biosynthesis. A)** Profile of four histone methylation marks on genes involved in the aliphatic GSL pathway. Read coverage normalized using a total number of mapped reads is indicated at the top right corner of each track in parenthesis. **B)** Profile of four histone methylation marks on genes involved in the indolic GSL pathway. Read coverage normalized using a total number of mapped reads is indicated at the top left corner of each track in parenthesis.

**Supplementary Fig. S9.**

**
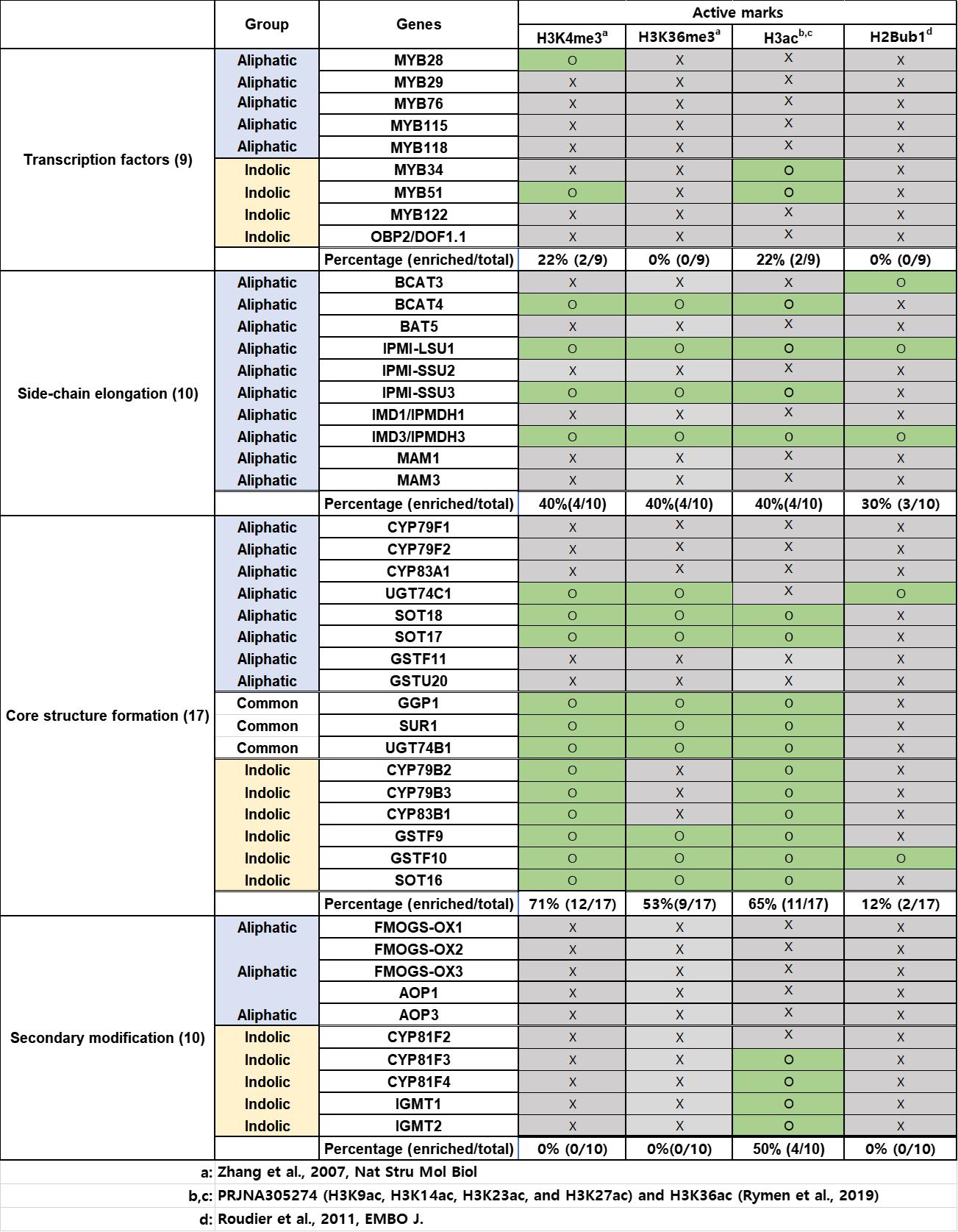
**

**Supplementary Fig. S9.** Enrichment of active histone methylation marks (H3K4me3 and H3K36me3) and H3 acetylation marks (combining H3K9ac, H3K14ac, H3K23ac, H3K27ac, and H3K36ac) on TF genes and three different biosynthetic phase genes. A table showing the status of H3K4me3, H3K36me3, H3ac, and H2Bub1 enrichment on 9 TFs, 10 ‘side-chain elongation’ phase genes, 17 ‘core structure formation’ phase genes, and 10 ‘secondary modification’ phase genes involved in the aliphatic and indolic GSL biosynthesis of Arabidopsis. Enrichment of H3K4me3, H3K36me3, H3ac, and H2Bub1 is indicated with “O” symbol in a green box. Meanwhile, no enrichment of H3K4me3, H3K36me3, H3ac, and H2Bub1 is indicated with “x” symbol in a gray box. Aliphatic and indolic GSL pathway group was respectively indicated with blue and orange boxes in the second column. Notably, H3ac histone marks were highly enriched in the indolic GSL pathway genes compared to those of aliphatic GSL pathway genes. Lowercases on the bottom of the table indicate the references used for the analysis of active histone mark enrichment of GSL pathway genes.

**Supplementary Fig. S10**

**
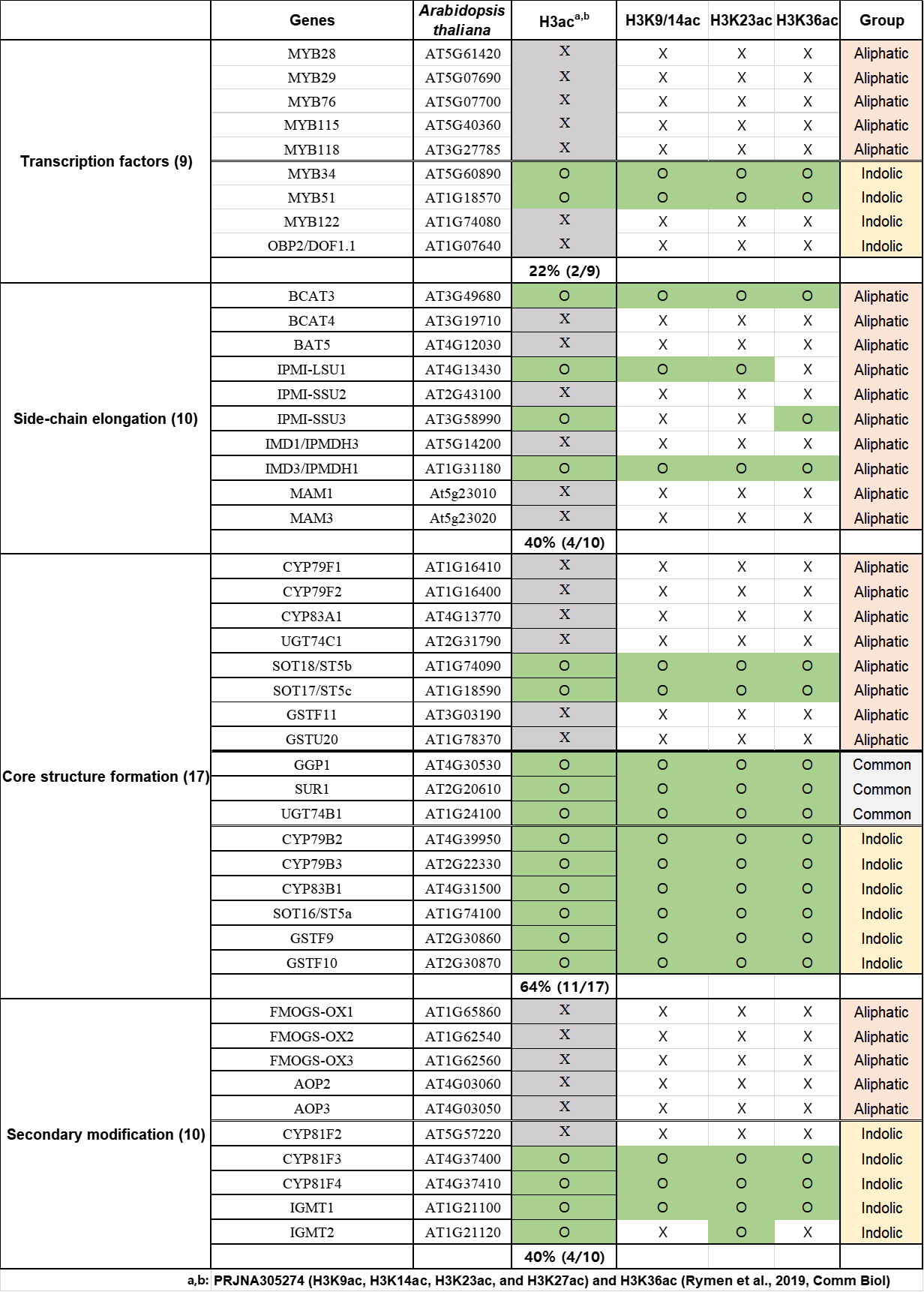
**

**Supplementary Fig. S10.** A table showing the status of different H3ac such as H3K9/14ac, H3K23ac, and H3K36ac enrichment on 9 TFs, 10 ‘side-chain elongation’ phase genes, 17 ‘core structure formation’ phase genes, and 10 ‘secondary modification’ phase genes involved in the aliphatic and indolic GSL biosynthesis of Arabidopsis. Enrichment of H3K9/14ac, H3K23ac, and H3K36ac on individual gene is indicated with “O” symbol in a green box. Meanwhile, no enrichment of H3ac marks is indicated with “x” symbol. Lowercases on the bottom of the table indicate the references and public ChIP-seq dataset (PRJNA305274) used for the analysis of active histone mark enrichment of GSL pathway genes.

**Supplementary Fig. S11**

**
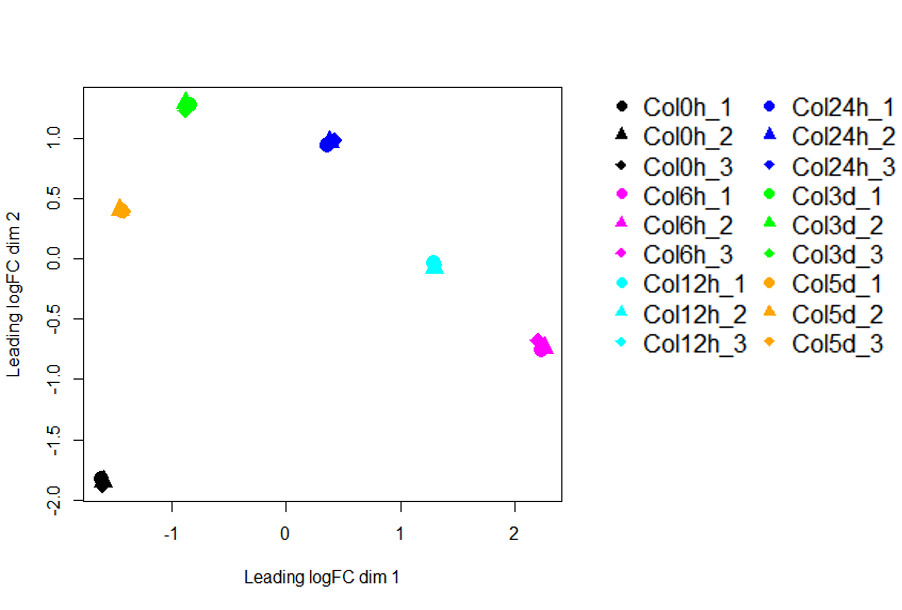
**

**Supplementary Fig. S11.** Multidimensional scaling (MDS) plot showing the close clustering of 18 RNA-seq samples according to same time point samples. Different time point samples are indicated with different colors: black, 0h (untreated); pink, 6h after wounding; sky blue, 12 h after wounding; blue, 24 h after wounding; green, 72 h(3 days) after wounding; orange, 120 h (5 days) after wounding.

**Supplementary Fig. S12**

**
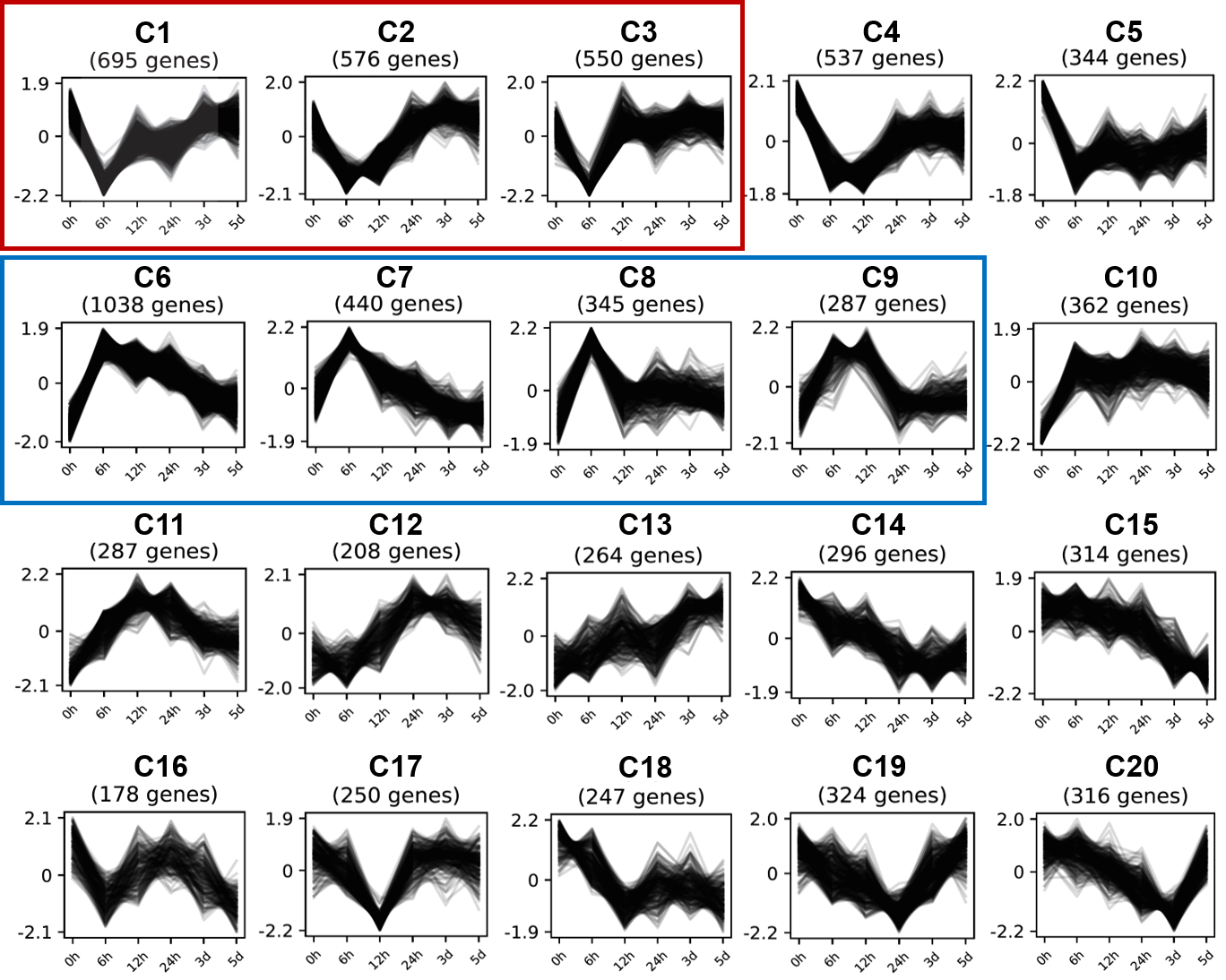
**

**Supplementary Fig. S12. Hierarchical clustering analysis of differentially expressed genes (DEGs) along wounding time course.** A total of 20 clusters (C1~C20) are generated based on transcriptional patterns of DEGs during the wounding time course (0 h, 6 h, 12 h, 24 h, 72 h, and 120 h after wounding). While aliphatic GSL pathway genes were highly enriched in the C1~C3 clusters (indicated with red line box), indolic GSL pathway genes were substantially enriched in the C1~C9 clusters (indicated with blue line box).

**Supplementary Fig. S13**

**
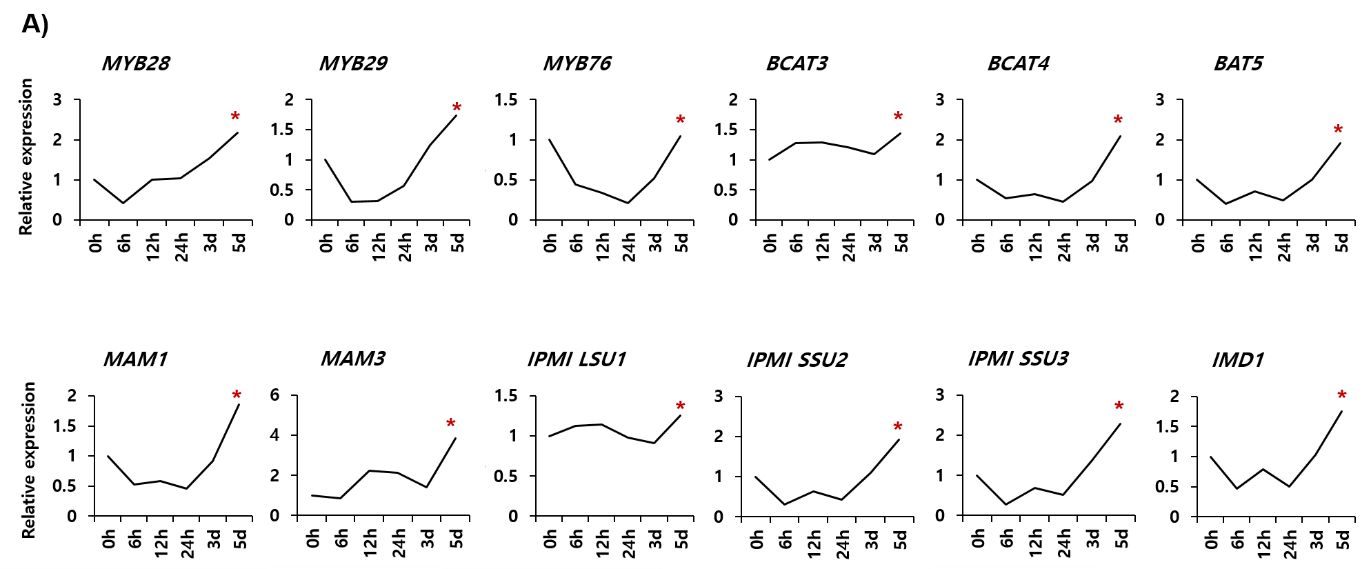
**

**
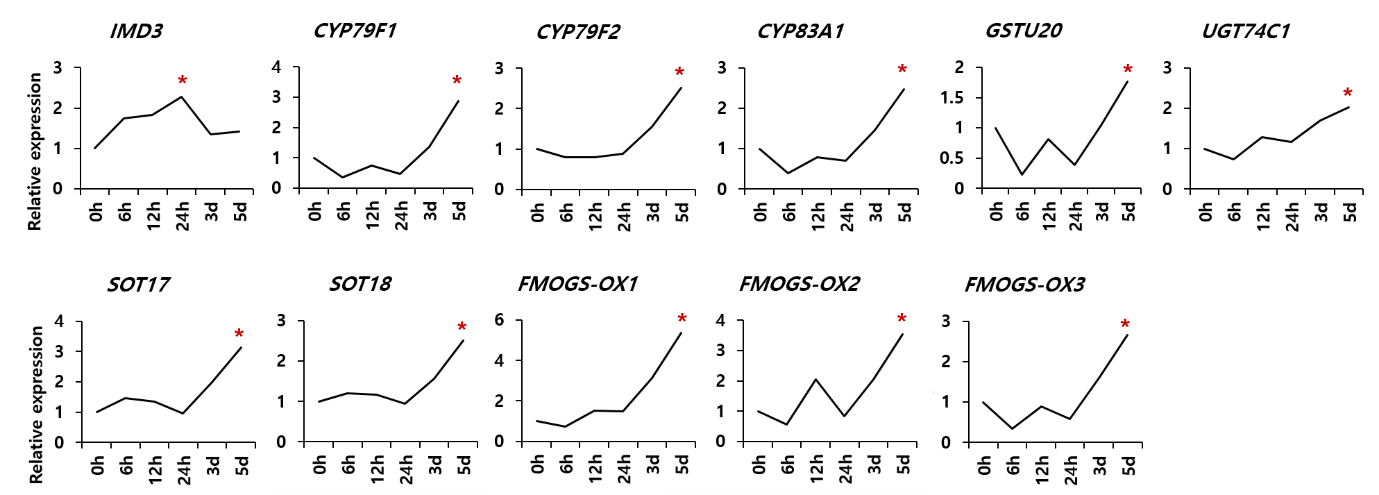
**

**
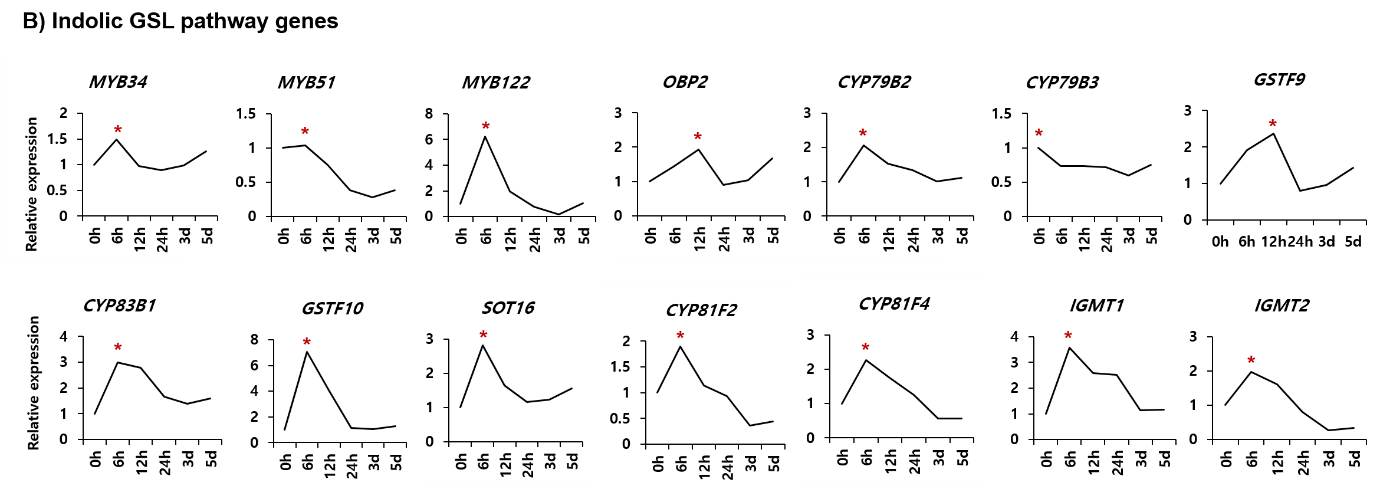
**

**Supplementary Fig. S13. Transcription patterns of genes involved in the aliphatic (A) and indolic (B) GSL biosynthetic process along six different time course after wounding of Arabidopsis A)** Transcript patterns of normalized RNA-seq reads of 23 aliphatic GSL pathway genes along six different time course after wounding treatment. **B)** Transcript patterns of normalized RNA-seq reads of 14 indolic GSL pathway genes along six different time course after wounding treatment. **A)~B)** Highest peak along six expression time points after wounding was indicated with red color asterisk.

**Supplementary Fig. S14**


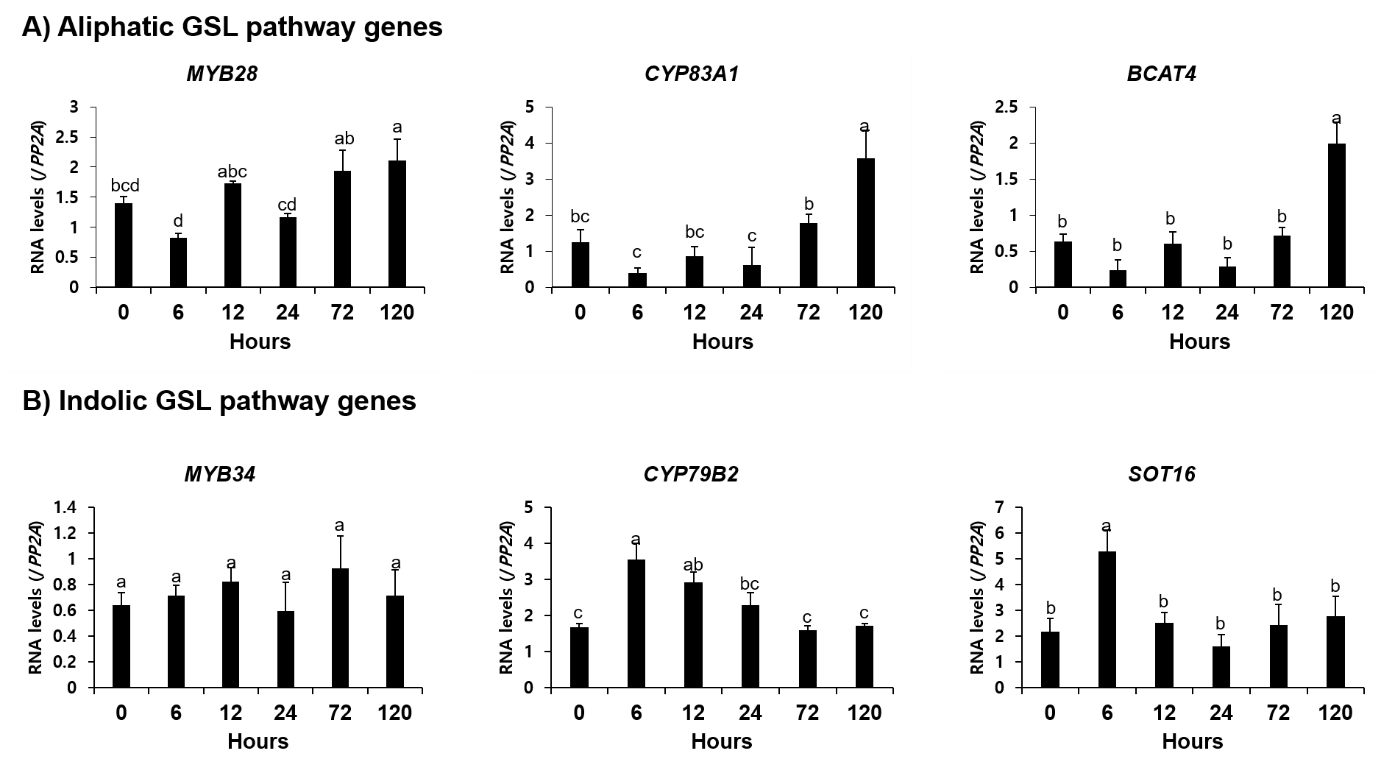


**Supplementary Fig. S14. Result of qRT-PCR analysis of several aliphatic (A) and indolic (B) GSL pathway genes along six different time points (0h, 6h, 12h, 24h, 72h, and 120 h) after wounding. A)** Result of qRT-PCR analysis of three aliphatic GSL pathway genes along six different time points (0h, 6h, 12h, 24h, 72h, and 120 h) after wounding. **B)** Result of qRT-PCR analysis of three indolic GSL pathway genes along six different time points (0h, 6h, 12h, 24h, 72h, and 120 h) after wounding. A)~B) The transcript level of each gene was normalized with relative expression of a reference gene, *PP2A* (*AT1G13320*) (*n* = 3). Significance was statistically determined using one-way analysis of variance (ANOVA) and Tukey’s post-hoc test (*p* < 0.05) and indicated with different letters above the line.

**Supplementary Fig. S15**

**
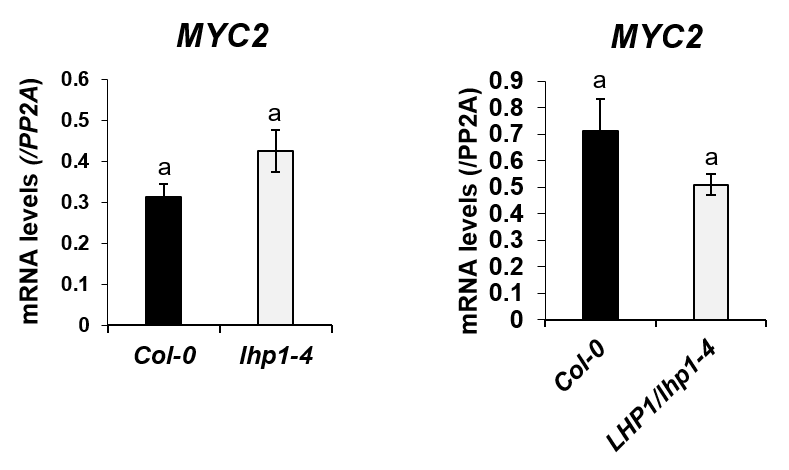
**

**Supplementary Fig. S15. Expression of *MYC2* between Col-0 and the *lhp1-4* mutant (left) and between Col-0 and *35S::LHP1/lhp1-4* transgenic line (right).** The transcript level of *MYC2* was normalized by one of a reference gene, *PP2A* (*AT1G13320*) (*n* = 3). Significance was statistically determined using one-way analysis of variance (ANOVA) and Tukey’s post-hoc test (*p* < 0.05) and indicated with different letters above the line.

**Supplementary Table S1.** List of genes belonging to the C1~C20 clusters based on the transcriptional patterns along time course after wounding.

[*separately uploaded*]

**Supplementary Table S2**. Primers used in this study.

| **For qRT-PCR** |  |
| --- | --- |
| **Primer name** | **Primer sequence (5'-3')** |
| MYB28_F | CATTCCCCAAAAAGCTGGGTTG |
| MYB28_R | CACGGGATCAATACCCTGTTCCAT |
| MYB29_F | TTCCCCAAAAAGCTGGACTAAAACG |
| MYB29_R | TCTGTTCTTTTGGGCAAATGTCTCG |
| MYB76_F | CTAACCCTAATCCAGTTGAGCCCATG |
| MYB76_R | CGATGGAAGAAGCTCTAGCTGCAACT |
| MAM1_F | GCCCGACAGCTCGCTAAACTCCGAGTAG |
| MAM1_R | CAGCGCCTCCCAAGTCGCCTCAATG |
| MAM3_F | AGCTAAACTCCGAGTAGACATCATGGAAGTTGG |
| MAM3_R | CTTCGCATACTTCAGTGCCTCCCAAGTTGC |
| CYP79F1_F | CATAATCGACGAGAGGGTCCAGTTG |
| CYP79F1_R | CCGGGTTCTTTAACATTTCCCCAAG |
| CYP79F2_F | CGAAATCAAAGCTCAATGCGTCGAA |
| CYP79F2_R | TAATGAGCGCTTGGGTGAATCCTG |
| CYP83A1_F | GGTTGTCAATGAGACGCTTGATCC |
| CYP83A1_R | TCCCGCCACTACAATATCCAAGATG |
| SOT17_F | GGACACGTTTGTGTCGATGTGG |
| SOT17_R | CACATAAGGCAATGGATCAGCTCTC |
| SOT18_F | GGCTGGAGACCGAAAGAGCCC |
| SOT18_R | CGTTTCAGGAGAGGGTTGGAGG |
| FMOGS-OX1_F | CCCTCGCAAGACAAGATGATGGAAG |
| FMOGS-OX1_R | GACCATTCTCTGGAATCCACGCTC |
| FMOGS-OX3_F | CTATGACGCCGTCGTGGTTTGCA |
| FMOGS-OX3_R | GTCCCTACTAATATCGGCACCACTCGC |
| AOP3_F | TGATGCGGAGTTGGGCTTACCTA |
| AOP3_R | AATGCTGCTGCGTATCTCGTCTTC |
| MYB34_F | GTGGATGGCGTACTCTCCCTGAA |
| MYB34_R | TTGCGGCCCACTTGTTACCCTTA |
| MYB51_F | CCCGAAAAAGCTGGACTCAAGAGAT |
| MYB51_R | GGTACCGGAGGTTATGCCCTTGT |
| MYB122_F | CGCCGTGTTGTAGAGCAGAAGG |
| MYB122_R | TCGTCTTGGCTAAACTCTCCACGTT |
| OBP2_F | GGCTGCCGGAGGAATAACAAGAAGG |
| OBP2_R | GCGGCTAAGTTCAAACCAATACCTCCG |
| CYP79B2_F | CTCAAACTCTTCGGATCTCACTACCAC |
| CYP79B2_R | CATTCCAATGATCGGCCATCCTGTG |
| CYP79B3_F | CGCTGATGAAATCAAACCAACCA |
| CYP79B3_R | CCTGCGACGGTCGTATCAGAGA |
| CYP83B1_F | CTTGACCCTAACCGCCCTAAACAAG |
| CYP83B1_R | TCAGTTCCCGGCACAACAATATCC |
| GGP1_F | CTGGAGAAATACGATGGCTTCGTTATCAGCGGAAG |
| GGP1_R | GTTCTGGTCCCTTCTTTGCTCTTCCGACTG |
| SUR1_F | GCTCCGGCAAAGGCAATTCTTAC |
| SUR1_R | AGCCAACGATTCGAACACGATCT |
| SOT16_F | GGGACAAACTCGTGAGGGTTTC |
| SOT16_R | CCCAATACGGTGGACACTGGTG |
| CYP81F2_F | GCATGGTCACAGGGAGACGCTACTA |
| CYP81F2_R | GCCGTGTCCGAACACTTTAAGAATC |
| CYP81F3_F | GGAAGCCATGGATGAGATCTTGCAG |
| CYP81F3_R | AAGCCTCGGGATGATTTAGCAAGC |
| CYP81F4_F | GCGAGGCATCCTGGAGAATACTTG |
| CYP81F4_R | GCGAGGCATCCTGGAGAATACTTG |
| IGMT1_F | GAAAGGGCGAGAGGGTCTACAGAG |
| IGMT1_R | GCTTGCTGAATCTCTCATCTGTACCC |
| IGMT2_F | ATCGAGCTGAGCCAATTTGCAGG |
| IGMT2_R | CGCGATTGTGAATCCAGTCTGGT |
| LHP1_F | GGAGGTGATGAGGAAGATGAAGAAGG |
| LHP1_R | GCAATAGACTGGAGATTCTCTAAAGGCTCC |
| MYC2_F | CGACGGCGGAGCTGGAGATTTAT |
| MYC2_R | GATTCGGGTTTTCGGTTATTGTGC |
| BCAT4_F | TGCTCAAGGGCAATGTTGTATCG |
| BCAT4_R | TCCCCTGTTTTGAATCCGGTCT |
| TGG1_F | CGTTAGATGTCCCGGCGGAAATTCGTC |
| TGG1_R | GCCCGCTCAGTTGCATCTTTGCTCTC |
| TGG2_F | TACCACCCAAGAGGGATGTTAAACG |
| TGG2_R | GCCTTGCGGAGAAAACATAGATGAC |
| TGG4_F | GGGGAGTGGACGAGAATGGGATAAC |
| TGG4_R | CTCCGAATCTTTGGAATAGAAGCTCAGCGTAG |
| TGG5_F | GAATGTTCAAGCATACCGTCTCTCGATAGCATG |
| TGG5_R | AGAAGCCTCCATATTCGTCTTCTAAAGTCTGG |
| PP2A_F | TATCGGATGACGATTCTTCGTGCAG |
| PP2A_R | GCTTGGTCGACTATCGGAATGAGAG |

| **For ChIP-qPCR** |  |
| --- | --- |
| **Primer name** | **Primer sequence (5'-3')** |
| MYB28_F1 | CTGACTGGAACCAGCGGATCTTAGGG |
| MYB28_R1 | GCTGGAGTGAAAATGTTAAGAGCTACGATAGC |
| MYB28_F2 | CTCTGTTCCTATATCTTGAGTTTTGTGAGAGG |
| MYB28_R2 | GTCGCGCCAGCCTCCCT |
| MYB28_F3 | GTTGCGGCTAAGGCCACTTCC |
| MYB28_R3 | CGGGGTACAACGATGATGGGGA |
| MYB29_F1 | CATGGAGGAGTAGTAGCTTGCAATGTCT |
| MYB29_R1 | TGTCGTCGAGTTTAAGGCAATAGACTGATAGATC |
| MYB29_F2 | GCGATGGGCTAACTATTTGAAACCTGA |
| MYB29_R2 | GCAAATGTCTCGCTATGACTGACCAC |
| MYB29_F3 | GAAGAAGCTGACAATACTGGAGGAGGA |
| MYB29_R3 | CCGGTCAACTCTAGTCCGGTAAGC |
| MYB76_F1 | GGATAGAGACTAGAGGGACAGCAAGGC |
| MYB76_R1 | CACCATTTACATCTTTTTACGACCGTTGTTC |
| MYB76_F2 | CATCTCTTATATCCACGACCACGGTG |
| MYB76_R2 | CCACACCGTTTCAGCCCTAAACTCG |
| MYB76_F3 | GATCTCCTTATGTCCGATTTCCCATCA |
| MYB76_R3 | GCCTAATCATAATCTTGGGCATGTCAAC |
| MYB34_F1 | CAGTGAGTACAATCTTCAGGTGTCTTCTAGT |
| MYB34_R1 | ACAAAGGATCATTAAGGCTCCATGTTACC |
| MYB34_F2 | GGCGGGACGAACTGACAACG |
| MYB34_R2 | ACGCGGTTAAGAAGCCTAGCGG |
| MYB34_F3 | GCAATGGAGTTCATTGATTCCTGGAACG |
| MYB34_R3 | CTACGTGACGATTACACGAATCTATCACAGC |
| MYB51_F1 | CTTTGTGGTAAGAAAACAGAGCAACTAGATC |
| MYB51_R1 | CTGAGAGGTGAAACAAAGTGTGAAATGG |
| MYB51_F2 | GTGACCATGATCTTGATAATGACAAGGCG |
| MYB51_R2 | GCCTCCACTTCCGATAATCTCAGAC |
| MYB51_F3 | CGAGGTTTCTTCACGAGGATGAAAACG |
| MYB51_R3 | CCATAGACATCATATTGACATGGTCAC |
| MYB122_F1 | CGTACAAGAACCTCCATCTCAATTTATTTTCCACTGAC |
| MYB122_R1 | GATTTGGCATGGCTCGTGTGTAGCCG |
| MYB122_F2 | CCATCACAAGCCCACAACAACAACAACTAC |
| MYB122_R2 | GAGTCCATGCTCCTTTCTTCAACCC |
| MYB122_F3 | CGTGACGCCGATCAATGAAGTTGATG |
| MYB122_R3 | CGGTCGAAACACACTACACACACAAAC |
| CYP79F1_F1 | CAAACCCTACGTGAGCATCACATGC |
| CYP79F1_R1 | GACCCAGTAGAGTGATTGATGCCATG |
| CYP79F1_F2 | CACCAGACGAAATCAAAGCTCAATGCG |
| CYP79F1_R2 | GTCCACTCCATGTTATTTGCCGGA |
| CYP79F1_F3 | CATCAAGATTTTGGACCGTTAAGCCTCG |
| CYP79F1_R3 | CGACTAAGCGTTGAAGAAGATAACAGAGAAAGA |
| CYP83A1_F1 | AGGAGCTTTGCTGTTCAATGTAACACTCA |
| CYP83A1_R1 | TCTGAAGCTGAAGGAGGTTTCCGA |
| CYP83A1_F2 | GTAGTGGCGGGAACAGATACTGCA |
| CYP83A1_R2 | GAATCACTGGTTCGATCCTTAGGGTTTC |
| CYP83A1_F3 | ACTGGTCTTGCTATGCACAAGTCGC |
| CYP83A1_R3 | TGTGAAGAGAAAGATAGAGAGACGATTGCC |
| FMOGS-OX1_F1 | GTATTATGTTTGAGAACAATATCTGTTATTTTTGGGGAA |
| FMOGS-OX1_R1 | ACCCCTCCAAATTGGATTAAGTATGGAAAC |
| FMOGS-OX1_F2 | AAGCATGTCTTTCTACCCGCG |
| FMOGS-OX1_R2 | GCAGCCACCCATTTGCTTTGAATTTC |
| FMOGS-OX1_F3 | TGTGAGTACCTCAACTGGATCG |
| FMOGS-OX1_R3 | CATGATTCGAGGAAATAAGAAGGATGAGAACT |
| CYP79B3_F1 | GGGGTACTATCGTCATTTCACACGTACC |
| CYP79B3_R1 | GTATCCATTTGCAAGGAAGAAGGAGAAGGAG |
| CYP79B3_F2 | GTGTAGCATAAAGCATGTTGACCACG |
| CYP79B3_R2 | GTCTGGCGCCGCCATTACAAGTTCC |
| CYP79B3_F3 | GATGCTAGCGAGGCTTTTGCAAGGG |
| CYP79B3_R3 | CCGACAATCTCAATTCTCCGACCAACACC |
| CYP81F2_F1 | GCCTAGTGACCAATACTAACATCCACCT |
| CYP81F2_R1 | GAGTGAAAATGGTGGATGGTCTATGTG |
| CYP81F2_F2 | CGGTGACCATTGGCGTAATCTCCG |
| CYP81F2_R2 | GGTCTCCGTAGTAGCGTCTCCCTG |
| CYP81F2_F3 | GGAGAAGAGAGATTGGTCGATGAACCTG |
| CYP81F2_R3 | GGTCTCTATGGATGGCCCAAGCG |
| IGMT1_F1 | ACTCATTCGTCCTAATATACGTGTCGCTGT |
| IGMT1_R1 | TGAGACTCACTTGATTTTATCGCTTGATAGATACA |
| IGMT1_F2 | GATCACGTAGAGTGCCTTGAAGGAGA |
| IGMT1_R2 | CGAGGGCGGCTTTGAGAACC |
| IGMT1_F3 | GGAAATCACTACCTGAAAACGGTAAAGTGG |
| IGMT1_R3 | AATGGGTGAAGCCAGAAGCTGCA |

| **For Sequential ChIP-qPCR** |  |
| --- | --- |
| **Primer name** | **Primer sequence (5'-3')** |
| CYP79F1_F1 | CATGATCCAGTTTGCTGCTAAGCTAAGAG |
| CYP79F1_R1 | ACACAGTGGAGCCGTGGAGTG |
| CYP79F1_F2 | CAAACCCTACGTGAGCATCACATGC |
| CYP79F1_R2 | GACCCAGTAGAGTGATTGATGCCATG |
| CYP79F1_F3 | CACCAGACGAAATCAAAGCTCAATGCG |
| CYP79F1_R3 | GTCCACTCCATGTTATTTGCCGGA |
| CYP79F1_F4 | CATCAAGATTTTGGACCGTTAAGCCTCG |
| CYP79F1_R4 | CGACTAAGCGTTGAAGAAGATAACAGAGAAAGA |
| CYP79F2_F1 | TCGAAATGTAAGCTCTAAAATGGTACTCTTAACTGA |
| CYP79F2_R1 | CTCTTGGGTAGGTAACCACGTTTAGATG |
| CYP79F2_F2 | GGTGTCGCCAGCTTCCTCCT |
| CYP79F2_R2 | CTGCCAAATCTGCGTCTCGCTCTC |
| CYP79F2_F3 | ACATGGAGTGGACACTTGGGGA |
| CYP79F2_R3 | GACGGGCAACATGAGGTGGG |
| CYP79F2_F4 | AGATTTCGGACCGTTAAGCCTCGA |
| CYP79F2_R4 | AGCGTTTTCGGGCAATGGATAGTCT |
| CYP83A1_F1 | ACCCTTCGGATTCAAATCTGTGTTGATG |
| CYP83A1_R1 | GACGAACCCAAGACTTATATCAATCACAATCC |
| CYP83A1_F2 | AGGAGCTTTGCTGTTCAATGTAACACTCA |
| CYP83A1_R2 | TCTGAAGCTGAAGGAGGTTTCCGA |
| CYP83A1_F3 | GTAGTGGCGGGAACAGATACTGCA |
| CYP83A1_R3 | GAATCACTGGTTCGATCCTTAGGGTTTC |
| CYP83A1_F4 | ACTGGTCTTGCTATGCACAAGTCGC |
| CYP83A1_R4 | TGTGAAGAGAAAGATAGAGAGACGATTGCC |
| FMOGS-OX1_F1 | GTATTATGTTTGAGAACAATATCTGTTATTTTTGGGGAA |
| FMOGS-OX1_R1 | ACCCCTCCAAATTGGATTAAGTATGGAAAC |
| FMOGS-OX1_F2 | TTAACGGGAAATGGAGTGTCCGG |
| FMOGS-OX1_R2 | GACCCAGAGACTAAGAACAAAGAAACCAAG |
| FMOGS-OX1_F3 | AAGCATGTCTTTCTACCCGCGCTAG |
| FMOGS-OX1_R3 | GCAGCCACCCATTTGCTTTGAATTTC |
| FMOGS-OX1_F4 | TGTGAGTACCTCAACTGGATCGCG |
| FMOGS-OX1_R4 | CATGATTCGAGGAAATAAGAAGGATGAGAACT |
| FMOGS-OX3_F1 | CGTAGTGCGAGTAAGTGATAACGGTAAGTC |
| FMOGS-OX3_R1 | GCAGGAATCTTGGTGGAGATGGG |
| FMOGS-OX3_F2 | CGAGTCTCTCCGAACCAATGTCC |
| FMOGS-OX3_R2 | CAACGCGAACCACCTCGGTC |
| FMOGS-OX3_F3 | CGCTTGCCCCTGGACTTGCT |
| FMOGS-OX3_R3 | CGTCCTGACAAGACTGCTGCCA |
| FMOGS-OX3_F4 | GAGAGGTCAAGAAGTTGACGGCG |
| FMOGS-OX3_R4 | GTCGGCACTATTGGCGCAGATCAT |
| GSTF11_F1 | TGGGCAGATAAAAGCAGCTAATCCACA |
| GSTF11_R1 | GCTGGAACTTGACCAAACGGCTAATACA |
| GSTF11_F2 | CCAAGTCTGGTAAGCCATGCGAC |
| GSTF11_R2 | GACGTAACCAAACCACTCAAACTGGT |
| GSTU20_F1 | AGTGCAGTTTGGCGATTAAAACCTCC |
| GSTU20_R1 | TCGGCAAGAAACACTAAACCCGTGG |
| GSTU20_F2 | TCTCGAACAAGAGCCCTTTACTCCT |
| GSTU20_R2 | CTTGTCCACGAAATCAGCCCAGA |
| GSTU20_F3 | GCGGTGCATGGAGAAAGAGAGTG |
| GSTU20_R3 | CACACATTCACTAAGGCTCTCTCGC |
| SOT17_F1 | CCTCTTCTCAAACGTAATCCACACGAG |
| SOT17_R1 | CGTGTCCTTTGGGTCTCTCCAT |
| SOT17_F2 | CGGAGATGGTGGCTCGTATTGATG |
| SOT17_R2 | GCAGTTCACCACATATTCCTCCAATGG |
| SOT18_F1 | AACTCTCTGTCTCTGAGTGCGTTTTCT |
| SOT18_R1 | GGCGGTTAGGGTTTCTGATTCCATT |
| SOT18_F2 | TCTTCCCTGAAGTTGATGTTCTCAAAGAC |
| SOT18_R2 | TCAAGATTGCTGACAGGTCCAAGC |
| SOT18_F3 | GAGAAAGACAGAGAGGATCGTCCTG |
| SOT18_R3 | AAGATCAGTCATTGCTCGGAACAAGAG |
| CYP79B2_F1 | CACAAGTATAAACTCGCCAGAACTCCTC |
| CYP79B2_R1 | GTTGGTGACCTGCCAAAATTCCTCGTG |
| CYP79B2_F2 | CCACGCATAAGGGTATTATAGTCATTTCAC |
| CYP79B2_R2 | GTGTATAGAGAAGAAGAGGACTCAAAG |
| CYP79B2_F3 | GTCTCTTTTGATTACTTTGGTCAAAGACAG |
| CYP79B2_R3 | GCCGCCATTACAAGCTCCTATTC |
| CYP79B2_F4 | CTAGGAACGGCGTTGACCACGATGATG |
| CYP79B2_R4 | CTAAGGTCACCGACCATAACCAACG |
| CYP79B3_F1 | ACTTGGCTATAACTACAAAAAGCTGAAGGGAC |
| CYP79B3_R1 | ACAGAGCCATAATAGTAGCTTTAACTGATTGAACTTACA |
| CYP79B3_F2 | GGGGTACTATCGTCATTTCACACGTACC |
| CYP79B3_R2 | GTATCCATTTGCAAGGAAGAAGGAGAAGGAG |
| CYP79B3_F3 | GTGTAGCATAAAGCATGTTGACCACG |
| CYP79B3_R3 | GTCTGGCGCCGCCATTACAAGTTCC |
| CYP79B3_F4 | GATGCTAGCGAGGCTTTTGCAAGGG |
| CYP79B3_R4 | CCGACAATCTCAATTCTCCGACCAACACC |
| CYP83B1_F1 | AAATCAAGAATGATGTCTAAATGAACAAAAGTTTATGAGA |
| CYP83B1_R1 | TTGGGTTACACAATAATACTGAAAGCGAGATCG |
| CYP83B1_F2 | GCCGGTTTAGTAGCGGCTGCA |
| CYP83B1_R2 | GGCGACGGCCACCGATTTTC |
| CYP83B1_F3 | AGCTCAAGACGAAGTGAGGAGTGTG |
| CYP83B1_R3 | GGTCTTGGCCGGAATATCATAGCCA |
| CYP83B1_F4 | GAGATACCTTTCGCTAACCTTCTCTACAA |
| CYP83B1_R4 | ACCATTGGTCACGCCATATCTACCAG |
| CYP81F2_F1 | CAACGCAAGAGAAATGGTCAAAAATACGG |
| CYP81F2_R1 | CCACGCACGATGCTTCACTCTAAATTAAC |
| CYP81F2_F2 | GCCTAGTGACCAATACTAACATCCACCT |
| CYP81F2_R2 | GAGTGAAAATGGTGGATGGTCTATGTG |
| CYP81F2_F3 | CGGTGACCATTGGCGTAATCTCCG |
| CYP81F2_R3 | GGTCTCCGTAGTAGCGTCTCCCTG |
| CYP81F2_F4 | GGAGAAGAGAGATTGGTCGATGAACCTG |
| CYP81F2_R4 | GGTCTCTATGGATGGCCCAAGCG |
| IGMT1_F1 | ACTCATTCGTCCTAATATACGTGTCGCTGT |
| IGMT1_R1 | TGAGACTCACTTGATTTTATCGCTTGATAGATACA |
| IGMT1_F2 | GATCACGTAGAGTGCCTTGAAGGAGA |
| IGMT1_R2 | CGAGGGCGGCTTTGAGAACC |
| IGMT1_F3 | CCAGACCGGATTCACAATCGCG |
| IGMT1_R3 | AGAAGGTGCTTGTGCCAAGGCA |
| IGMT1_F4 | GGAAATCACTACCTGAAAACGGTAAAGTGG |
| IGMT1_R4 | AATGGGTGAAGCCAGAAGCTGCA |
| FLC_F | GGCACAAAGCCCTCTCGGAGA |
| FLC_R | ATCGCCGGAGGAGAAGCTGTAGA |
| PP2A_F | GATTATCTTCCACTTACAGCTCTTATTGAGAGTCAG |
| PP2A_R | TAAAGATCGGATGTTGTCAAGTGTATCTAAAGAAGG |

**Supplementary Table S3.** List of public ChIP-seq dataset analyzed in this study.

| **Type** | **samples** | **SRR #** |
| --- | --- | --- |
| **ChIP-seq** | H3K4me3 IP | SRR970144 |
|  | H3K9me2 IP | SRR3087128 |
|  | H3K27me3 IP | SRR1931604 |
|  | H3K36me3 IP | SRR3087130 |
|  | Input | SRR970148 |
|  | H3K9ac IP | SRR2977195 |
|  | H3K14ac IP | SRR2979528 |
|  | H3K23ac IP | SRR2979547 |
|  | H3K36ac IP | SRR2932293 |
|  | Input | SRR2932294 |
|  | Col-0 Input | SRR6453479 |
|  | Col-0 H3K27me3 IP rep1 | SRR6453477 |
|  | Col-0 H3K27me3 IP rep2 | SRR6453478 |
|  | *clf-29;swn-4* Input | SRR6453488 |
|  | *clf-29;swn-4* H3K27me3 IP rep1 | SRR6453486 |
|  | *clf-29;swn-4* H3K27me3 IP rep2 | SRR6453487 |
|  | Input | SRR3087683 |
|  | LHP1-GFP | SRR3087684 |
|  | H3K27me3 Col-0 input | SRR11095968 |
|  | H3K27me3 Col-0 IP rep 1 | SRR11095969 |
|  | H3K27me3 Col-0 IP rep 2 | SRR11095970 |
|  | H3K27me3 *lhp1-4* (*tfl2-2*) input | SRR1931609 |
|  | H3K27me3 *lhp1-4* (*tfl2-2*) IP | SRR1931608 |

-END-
